# Deciphering the metabolic response of *M* *ycobacterium tuberculosis* to nitrogen stress

**DOI:** 10.1111/mmi.13091

**Published:** 2015-07-17

**Authors:** Kerstin J. Williams, Victoria A. Jenkins, Geraint R. Barton, William A. Bryant, Nitya Krishnan, Brian D. Robertson

**Affiliations:** ^1^ MRC Centre for Molecular Bacteriology and InfectionDepartment of MedicineImperial College LondonLondonSW7 2AZUK; ^2^ Centre for Integrative Systems Biology and BioinformaticsImperial College LondonLondonSW7 2AZUK; ^3^ Present address: Department of Microbial and Cellular SciencesUniversity of SurreyGuildfordGU2 7XHUK; ^4^ Present address: Department of ParasitologyLiverpool School of Tropical MedicineLiverpoolL3 5QAUK

## Abstract

A key component to the success of *M* *ycobacterium tuberculosis* as a pathogen is the ability to sense and adapt metabolically to the diverse range of conditions encountered *in vivo*, such as oxygen tension, environmental pH and nutrient availability. Although nitrogen is an essential nutrient for every organism, little is known about the genes and pathways responsible for nitrogen assimilation in *M* *. tuberculosis*. In this study we have used transcriptomics and chromatin immunoprecipitation and high‐throughput sequencing to address this. In response to nitrogen starvation, a total of 185 genes were significantly differentially expressed (96 up‐regulated and 89 down regulated; 5% genome) highlighting several significant areas of metabolic change during nitrogen limitation such as nitrate/nitrite metabolism, aspartate metabolism and changes in cell wall biosynthesis. We identify GlnR as a regulator involved in the nitrogen response, controlling the expression of at least 33 genes in response to nitrogen limitation. We identify a consensus GlnR binding site and relate its location to known transcriptional start sites. We also show that the GlnR response regulator plays a very different role in *M* *. tuberculosis* to that in non‐pathogenic mycobacteria, controlling genes involved in nitric oxide detoxification and intracellular survival instead of genes involved in nitrogen scavenging.

## Introduction

Nitrogen is a key component of all biological macromolecules, found in information‐encoding polymers such as DNA and RNA, and structural molecules such as proteins and components of the cell envelope. As such nitrogen assimilation is essential for life, and organisms such as bacteria have evolved a variety of mechanisms to obtain nitrogen from their surroundings and subsequently assimilate it into cellular macromolecules. Historically, the bulk of work published on nitrogen metabolism in bacteria has concentrated on model organisms such as *Escherichia coli* (see Reitzer, 2003; Leigh and Dodsworth, 2007 for reviews), but recently there have also been a number of reports on nitrogen metabolism in Actinomycetes, including the saprophyte *Mycobacterium smegmatis* (Amon *et al*., 2008; Khan *et al*., 2008; Harper *et al*., 2010; Behrends *et al*., 2012; Jenkins *et al*., 2012; 2013; Williams *et al*., 2013), and the obligate human pathogen *Mycobacterium tuberculosis* (Read *et al*., 2007; Carroll *et al*., 2008; Malm *et al*., 2009; Tan *et al*., 2010; Akhtar *et al*., 2013; Gouzy *et al*., 2013; Williams *et al*., 2013). However, there has been no work published on the global *M. tuberculosis* nitrogen stress response and its regulation, a gap that is addressed here.

Ammonium is the preferred nitrogen source for many bacteria, but there is recent evidence that asparagine (converted to ammonium for assimilation) may be the preferred nitrogen source in *M. tuberculosis* (Gouzy *et al*., 2014). *M. tuberculosis* can also use alternative nitrogen sources for growth such as urea (Lin *et al*., 2012) and aspartate (Gouzy *et al*., 2013). Once inside the cell (either directly or indirectly), ammonium is assimilated into L‐glutamate and L‐glutamine, the two major biosynthetic nitrogen donors. This is either via the low ammonium affinity glutamate dehydrogenase (GDH) enzyme, when nitrogen is plentiful, or by the energy‐requiring, high‐affinity glutamine synthetase/glutamate synthase (glutamine:2‐oxoglutarate aminotransferase; GS/GOGAT) enzymes when nitrogen is scarce (for reviews see Harper *et al*., 2008; Amon *et al*., 2010). *M. tuberculosis* only appears to encode the high affinity GS/GOGAT system, unlike *M. smegmatis*, which encodes both (Amon *et al*., 2009). Key nitrogen control enzymes undergo post‐translational modifications in response to nitrogen limitation. The GlnK (PII) signalling protein is adenylylated on a conserved tyrosine residue by GlnD, in response to nitrogen limitation (Williams *et al*., 2013). This modification is associated with the dissociation of the PII protein from the AmtB porin channel (Javelle and Merrick, 2005; Radchenko *et al*., 2010), permitting increased ammonium influx (Gruswitz *et al*., 2007). Glutamine synthetase undergoes a post‐translational de‐adenylylation by GlnE during nitrogen limitation, making it fully active (Carroll *et al*., 2008) and ensuring maximal glutamine and glutamate synthesis during nitrogen austerity. However, many gaps remain in our knowledge of nitrogen metabolism and regulation in mycobacteria, for example, the signal indicating nitrogen cellular status is unknown. We have shown that in *M. smegmatis*, the intracellular ratio of 2‐oxoglutarate : glutamine increases during nitrogen limitation and then decreases when nitrogen is available, indicating that this may be the intracellular signal (Behrends *et al*., 2012). However, how this signal is detected and then manifested in transcriptional and post‐translational responses remains unclear. What, if any, role PII proteins play in the control of the nitrogen response in mycobacteria control is also unknown. In *E. coli*, PII‐UMP controls the response‐regulator NtrC (Pioszak *et al*., 2000), but PII‐AMP in mycobacteria does not mediate the transcriptional response to nitrogen limitation (Williams *et al*., 2013).

The transcriptional response to nitrogen limitation in enteric bacteria is mediated by the two‐component system NtrBC, which activates expression of over 100 genes (Zimmer *et al*., 2000; Reitzer, 2003), but this system is missing from the Actinomycetes. The equivalent function in *Corynebacterium glutamicum* is performed by the TetR‐type response‐regulator AmtR, which controls transcription of over 33 genes (Beckers *et al*., 2005; Burkovski, 2007), while in *Streptomyces*, the OmpR‐type response‐regulator GlnR controls nitrogen metabolism (Fink *et al*., 2002), regulating at least 50 nitrogen response genes in *Streptomyces coelicolor* and at least 44 genes in *Streptomyces venezuelae* (Tiffert *et al*., 2008; 2011; Pullan *et al*., 2011). *M. tuberculosis* contains a GlnR homolog (Rv0818) with 61% identity to that from *S. coelicolor* (Tiffert *et al*., 2011), but the evidence for an AmtR homologue is weak, with Rv3160c only 28% identical to AmtR from *C. glutamicum* (Harper *et al*., 2008), and to date, no role has been reported for AmtR in mycobacteria. We have shown that GlnR is the main nitrogen response regulator in *M. smegmatis* and described the complete GlnR nitrogen‐response regulon. We demonstrated that GlnR regulates the expression of more than 100 genes during nitrogen limitation (Jenkins *et al*., 2013), many of which are involved in nitrogen metabolism and nitrogen scavenging, and used the Active Modules algorithm, AMBIENT (Bryant *et al*., 2013), to identify key metabolic reactions and pathways altered in response to nitrogen stress (Williams *et al*., 2013). We also demonstrated that the aspartate D48 residue is essential for the GlnR‐mediated transcriptional response to nitrogen limitation in *M. smegmatis* (Jenkins *et al*., 2012); this residue was recently shown to be critical for stabilization of the GlnR homodimers required for its function (Lin *et al*., 2014).

The aim of this study was to extend our global nitrogen stress analyses to the obligate human pathogen *M. tuberculosis*, which has to deal with a more restricted set of environmental variables in terms of nitrogen sources, and to delineate the GlnR regulon. We combined genome‐wide expression profiling, comparing a *glnR* mutant to the wild‐type strain during nitrogen limited growth, global analysis of GlnR–DNA interactions by chromatin immunoprecipitation and high‐throughput sequencing (ChIP‐seq), and transcriptomics over nitrogen run‐out. We identified key changes in the metabolic network in response to nitrogen limitation, showing that the main nitrogen metabolism‐related response is the production of ammonium both through the reduction of nitrite and from aspartate; however, *M. tuberculosis* does not appear to scavenge ammonium or other nitrogen sources (e.g. urea) from the environment. We found changes in general metabolism such as an increased methylcitrate–isocitrate lysase cycle and changes in the cell wall phthiocerol dimycoceroserate (PDIM) and peptidoglycan make up. We show that GlnR is a global regulator in mycobacteria, controlling the expression of at least 67 genes in response to nitrogen stress, although intriguingly only the minority of GlnR controlled genes are directly involved in nitrogen metabolism. We identified 35 GlnR‐binding sites, 22 of which controlled the differential gene expression of at least 19 genes in nitrogen limitation. This includes divergently transcribed genes, and both up‐ and down‐regulated genes, showing GlnR functions as an activator and repressor of transcription. Using the 25 GlnR binding sites found in intergenic regions, we identified a consensus DNA‐binding motif. Overall, this work re‐illustrates the complexity of metabolism in mycobacteria and how *M. tuberculosis* has adapted its nitrogen metabolism for life inside a vertebrate host.

## Results

### Defining nitrogen stress conditions

**Figure 1:**
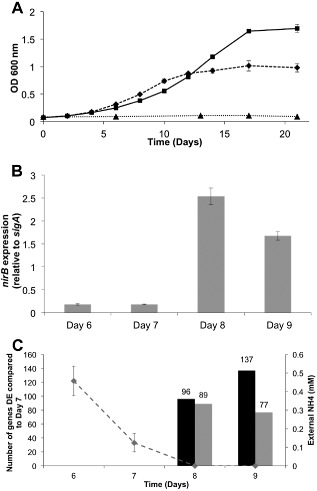
Effect of nitrogen limitation on *M* *. tuberculosis* growth and gene expression. A. *M* *. tuberculosis* was grown in nitrogen‐free Sauton's medium (filled triangles), or containing 1 mM ammonium chloride (nitrogen limiting, filled diamonds), or 30 mM ammonium chloride (nitrogen excess, filled squares). B. The expression of *nir* *B* was measured by qRT‐PCR using RNA from three independent cultures, with *sig* *A* as internal control. Fold change was calculated as a ratio of the arbitrary expression units, standardised to *sig* *A*. Ct values for *sig* *A* did not change significantly over nitrogen run‐out. C. The number of genes showing greater than twofold change in differential expression (DE) over nitrogen run‐out at days 8 and 9 compared with day 7. Black bars show an increase in DE; grey bars a decrease in DE. The concentration of external ammonium concentration (mM) in the growth medium as measured by AquaQuant analysis is also shown (dashed line).

*Mycobacterium tuberculosis* was grown in nitrogen‐free Sauton's medium containing either 1 mM ammonium chloride (nitrogen limiting) or 30 mM ammonium chloride (nitrogen excess) (Fig. 1A). External ammonium levels were monitored and shown to be completely depleted in the nitrogen limiting medium by day 8 (Fig. 1C). To ensure that a transcriptional response was induced in our nitrogen limiting conditions, transcript levels of a gene previously shown to be highly induced in nitrogen limitation in *M. smegmatis* (*nirB*) (Jenkins *et al*., 2012) was monitored. The expression level of *nirB* was induced in the nitrogen limiting medium at day 8 relative to *sigA*, correlating with the point of external ammonium depletion (Fig. 1B). Gene expression was unchanged in the nitrogen excess medium over these time points (data not shown).

### Expression profiling of the nitrogen stress response

RNA samples (three biological replicates, 1 mM ammonium chloride) were taken at three time points (days 7, 8 and 9) and applied to an *M. tuberculosis* tiling microarray. Fully annotated data have been deposited in BμG@Sbase and ArrayExpress, and can be viewed in File S1. The data were analysed as described to determine which genes were significantly differentially expressed (DE) during nitrogen limitation (days 8 and 9) compared with nitrogen replete (day 7). Genes were considered to be significantly differentially expressed if their expression changed greater than twofold compared with their expression at day 7 with a false discovery rate (FDR) corrected *P*‐value of < 0.1. A complete list of significantly DE genes identified by these criteria can be viewed in File S2. At nitrogen run‐out, day 8, 185 genes were DE (96 up and 89 down) and at day 9, 214 genes were DE (137 up and 77 down) greater than twofold (Fig. 1C). In total, expression of approximately 5% of the genome was altered significantly upon nitrogen stress.

### Metabolic analysis of the nitrogen stress response

**Figure 2:**
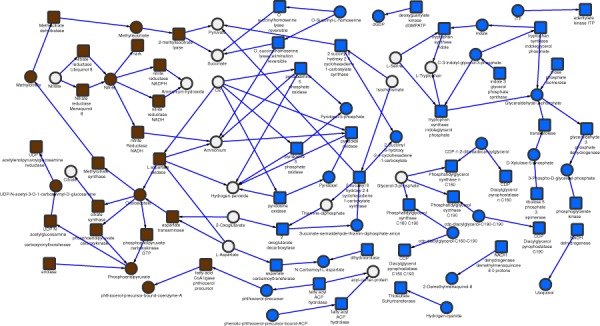
Ambient modules are presented in their metabolic network context, illustrating a redirection of metabolism. Squares represent enzymatic reactions and circles represent metabolites. Grey (blue online) represents negatively regulated, and dark (brown online) positively regulated reactions. No definitive claims can be made about fluxes, so arrows are only indicative and taken from the identity or context of the relevant reaction. For reference, reactions with gene numbers are given in Fig. S1.

To obtain a more holistic view of how changes in gene expression might reflect changes in metabolism, we used AMBIENT (Bryant *et al*., 2013) and a genome scale metabolic model (Jamshidi and Palsson, 2007) to identify areas of metabolism that were affected in nitrogen limitation, a method we have previously applied successfully to *M. smegmatis* (Williams *et al*., 2013). The modules identified ultimately depend on the quality of the genome scale metabolic model, and they do not always fit neatly into traditional pathways. We discovered a large interconnected network of reactions linking 102 enzymes, with both increased (37) and decreased (65) activity (Fig. 2). The genes annotated with each of these metabolic reactions have been overlaid on the same diagram (Fig. S1) to indicate their position in the network. The reactions were grouped into 19 modules according to the AMBIENT analysis (Table 1). Nitrate/nitrite metabolism and nitric oxide detoxification are the major responses to nitrogen stress, and are regulated by GlnR (see discussion later). These potentially produce ammonium for growth as well protecting the cell from toxic effects of NO. A wide range of other biosynthetic activities also respond to nitrogen stress, but most are not obviously linked directly to nitrogen metabolism (Table 1). The methylcitrate/isocitrate lyase in the glyoxylate shunt are up‐regulated, and PDIM and peptidoglycan biosynthesis are increased, indicating changes in energy metabolism and cell envelope biosynthesis, whereas phosphatidyl glycerol shows decreased activity, with links in the network suggesting redistribution of metabolic activity. The *M. tuberculosis* network identified is more interconnected than that seen in *M. smegmatis* (Jenkins *et al*., 2013), but smaller in size, with a number of notable differences. There is no large decrease in modules associated with growth, such as DNA, RNA and protein synthesis, perhaps suggesting *M. tuberculosis* can maintain its growth rate in the range of habitats encountered *in vivo*.

**Table 1:** AMBIENT modules differentially regulated in response to nitrogen stress

Module	q‐value	No. of reactions	No. of metabolites	Specific function(s)	Genes
**Up**					
u1	< 0.001	6	1	Nitrite/nitrate reductase activity	Rv0252, Rv0253, Rv0261, Rv0267, Rv1161, Rv1162, Rv1163, Rv1164, Rv1737, Rv2329, Rv2391
u2	< 0.001	11	5	Citrate/oxaloacetate metabolism	Rv0211, Rv0337, Rv0467, Rv0482, Rv0889, Rv0896, Rv1023, Rv1130, Rv1131, Rv1315, Rv1475, Rv1595, Rv1915, Rv3565
u3	0.01	1	1	Phthiocerol precursor ligase	Rv2930
**Down**					
d1	< 0.001	9	5		Rv1408, Rv1436, Rv1437, Rv1438, Rv1449, Rv1611, Rv1612, Rv1613
d2	0.037	2	1	Phosphatidylglycerol synthase	Rv1822, Rv2289, Rv2746
d3	0.037	2	1	Pyridoxine 5 phosphate oxidase	Rv2607
d4	0.037	2	1	Pyridoxal oxidase	Rv2607
d5	0.037	2	1	Phosphatidylglycerol synthase	Rv1822, Rv2289, Rv2746
d6	0.037	2	1	Phosphatidylglycerol synthase	Rv1822, Rv2289, Rv2746
d7	0.037	2	1	Aspartate carbamoyltransferase	Rv1380, Rv1381
d8	0.037	2	1	*O*‐succinylhomoserine lyase	Rv0391, Rv1079
d9	0.039	1	1	Fatty acyl ACP hydrolase	Rv2928
d10	0.041	1	1	NADH dehydrogenase demethylmenaquinone 8	Rv0082, Rv3145, Rv3146, Rv3147, Rv3148, Rv3149, Rv3150, Rv3152, Rv3153, Rv3154, Rv3155, Rv3156, Rv3157, Rv3158
d11	0.041	1	1	NADH dehydrogenase (ubiquinol)	Rv0082, Rv3145, Rv3146, Rv3147, Rv3148, Rv3149, Rv3150, Rv3152, Rv3153, Rv3154, Rv3155, Rv3156, Rv3157, Rv3158
d12	0.041	1	1	Thiosulfate sulfurtransferase	Rv2291
d13	0.041	1	1	Fatty acyl ACP hydrolase	Rv2928
d14	0.045	1	1	Adenylate kinase ITP	Rv0733
d15	0.045	3	2	2‐Oxoglutarate decarboxylase	Rv0555
d16	0.045	1	1	Deoxyguanylate kinase dGMPATP	Rv1389

Up‐ and down‐regulated modules found using AMBIENT in nitrogen stress, indicating the number of reactions and metabolites present in each. Scores are calculated by AMBIENT, taking into account the logarithm of the fold change of the genes associated with each reaction and the connectivity in the metabolic network of each metabolite in the module. The number of genes associated with these reactions according to the *M. tuberculosis* model (Jamshidi and Palsson, 2007) is also shown. Functional assignments are based on manual inspection of the member reactions of the modules.

### Determination of GlnR controlled genes in nitrogen stress

In order to determine the genes regulated by GlnR, we constructed a GlnR null mutant. Substitution of the *glnR* gene by the hygromycin cassette was confirmed by polymerase chain reaction (PCR) (Fig. S2) and western analysis using a GlnR specific antibody (Fig. S2). The *M. tuberculosis glnR* KO mutant did not grow with nitrate as the sole nitrogen source, or show any phenotype under nitrogen limitation, and did not up‐regulate *nirB* expression upon nitrogen limitation (data not shown). There was also no difference in the response of the mutant to environmental stresses including pH 3, hydrogen peroxide or a nitric oxide donor (data not shown). The expression profiles of *M. tuberculosis* wild type and *glnR* deletion mutant grown in nitrogen limiting conditions were then obtained. Cells were harvested 1 day after nitrogen run‐out, total RNA was extracted and cDNA hybridised to the *M. tuberculosis* microarray as described. Data were normalized and genes were considered significantly differentially expressed if they exhibited a difference in gene expression of greater than twofold with an FDR corrected *P*‐value of < 0.1. Fully annotated microarray data have been deposited in BμG@Sbase and ArrayExpress. A total of 52 genes were significantly up‐regulated and 15 significantly down regulated (File S3). This indicates that GlnR mediates (directly or indirectly) the expression of at least 67 genes.

### Identification of GlnR binding sites during nitrogen stress

**Figure 3:**
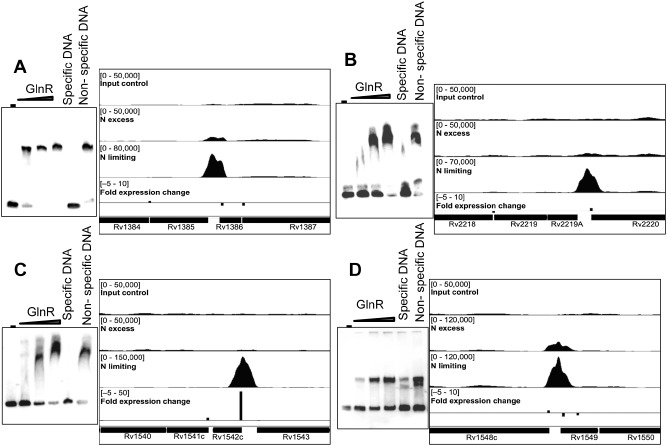
Novel GlnR binding sites identified upstream of differentially expressed genes, with corresponding EMSA to confirm specific GlnR binding. EMSA were performed by incubating increasing amounts of His‐GlnR recombinant protein with labelled DNA corresponding to the promoter regions of the genes downstream of the GlnR binding site. GlnR binding was visualised in IGV. The top track represents input control DNA, the second track represents GlnR binding in nitrogen excess and the third track represents GlnR binding in nitrogen limiting conditions. Bar height is representative of fold change in gene expression in the wild type compared with the *glnR* KO mutant in nitrogen limitation. Levels of gene expression are indicated in the bottom track. (A) Peak 13, Rv1386 (*PE15*); (B) peak 20, Rv2219A; (C) peak 17, Rv1542c (*glbN*); (D) peak 18, Rv1548c (*PPE22*). The negative control Rv1360 DNA showed no GlnR binding (Fig. S4).

**Figure 4:**
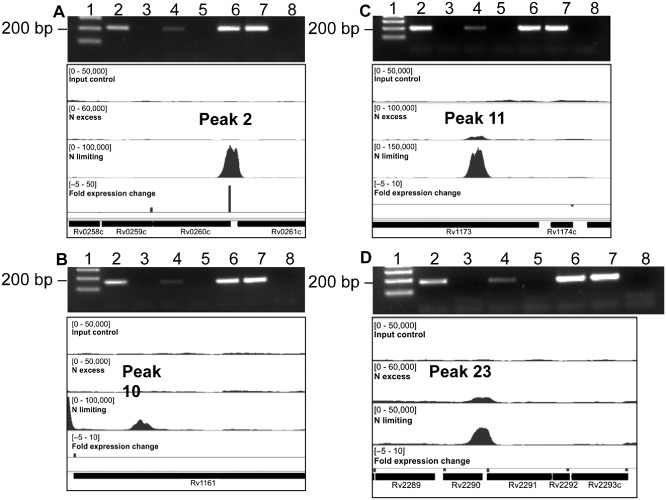
Novel GlnR binding sites identified upstream of differentially expressed genes, with corresponding DNA enrichment verified by qPCR. DNA enrichment for an additional four novel GlnR‐binding regions was observed in nitrogen limitation, but not nitrogen excess using rate‐limiting qPCR. (A) Peak 2, Rv0260c (*nna* *R*); (B) peak 10, Rv1161 (*narG*); (C) peak 11, Rv1173 (*fbi* *C*); (D) peak 23, Rv2291 (*sse* *B*). Lane 1, size marker, 200 bp arrowed. Lane 2, ChIP nitrogen excess. Lane 3, nitrogen excess no antibody control. Lane 4, ChIP nitrogen limiting. Lane 5, nitrogen limiting no antibody control. Lane 6, input nitrogen excess. Lane 7, input nitrogen limiting. Lane 8, no DNA control. A negative control region showed no enrichment (Fig. S3).

In order to identify directly regulated GlnR genes, we used ChIP‐seq to identify the location of GlnR binding sites in the genome during nitrogen limitation. Cells were grown in 1 mM (limiting) or 30 mM (excess) ammonium chloride, and DNA–protein complexes were cross‐linked 1 day after ammonium depletion; nitrogen excess samples were cross‐linked at the same time point, cells were then lysed and the DNA sheared by sonication. GlnR‐bound DNA fragments were immunoprecipitated using affinity‐purified anti‐GlnR polyclonal antibody. Prior to Illumina library construction, we performed quantitative PCR on the nitrite reductase (*nirB*) promoter region to confirm the enrichment of known GlnR binding regions in nitrogen limited cells compared with nitrogen replete cells; a gene thought not to be GlnR regulated (Rv1360) was included as a negative control (Fig. S3). The immunoprecipitated DNA was then prepared for next generation sequencing using the Illumina ChIP‐seq library kit. Sequencing of the DNA libraries generated approximately 144 million reads per sample which were mapped to the *M. tuberculosis* genome. All ChIP‐seq data files have been deposited into ArrayExpress (accession number E‐MTAB‐2492). GlnR binding regions were identified using the peak‐calling algorithm SISSRs (Narlikar and Jothi, 2012), with peaks defined as significant if they showed greater than fivefold enrichment in the immunoprecipitated sample compared with the input control DNA with a *P* value of < 0.005. This identified 36 putative GlnR binding sites in nitrogen limitation (Table 2). Of the 36 GlnR binding sites in nitrogen limitation, one (peak 12) was incorrectly identified by SISSRs as a GlnR binding site due to an excess of ribosomal RNA in this region; this was excluded from further analyses. Of the remaining 35 GlnR binding sites identified in nitrogen limitation, 26 were located in intergenic regions and 9 were located within genes. Although identification of the *nirB* promoter region (peak 1) as a GlnR binding site corroborated our ChIP‐seq data (Table 2, Fig. S3), further validation of four novel GlnR binding sites identified in this study by electromobility shift assays (EMSA) were performed. Two hundred base pair DNA regions, representing the binding regions from peaks 13, 17, 18 and 20, were incubated with increasing concentrations of recombinant GlnR protein. Fig. 3 shows that GlnR binds specifically to these promoter regions with a protein‐concentration dependent shift; the negative control Rv1360 DNA displayed no GlnR binding (Fig. S4). In addition, DNA enrichment of another four novel GlnR binding sites identified in this study (peaks 2, 10, 11 and 23) was verified by rate‐limiting qPCR. Enrichment of these four genomic regions by GlnR IP was observed in nitrogen limitation and not in nitrogen excess (Fig. 4); negative control region showed no enrichment (Fig. S3).

**Table 2:** GlnR binding regions identified by ChIP‐seq and corresponding gene expression fold change (wild type vs *gln* *R* deletion strain) in *M* *. tuberculosis* during nitrogen limitation

Peak no.^a^	Coordinates^b^	Peak Intensity^c^	Adjacent gene(s)^d^	Fold change DE^e^ (wt v glnR ko)	Gene name	Msmeg GlnR regulon^g^	Lsr2‐GlnR overlap^h^	TSS^i^	TSS (Exp or Strv)^j^
**1**	302811–302851	54.53	Rv0252*	36.77	nirB	Y	Y	302853	Both
**2**	312651–312691	25.91	Rv0260c*	44.63	nnaR	Y	Y	312659	Exp
**3**	314191–314231	11.84	Rv0261c	10.65	narK3	Y		None	
**4**	559811–559851	11.21	Rv0469	Not Sig	umaA			559862	Both
**5**	560371–560411	7.77	Within Rv0469	Not Sig	umaA		y	N/A	
**6**	1077911–1077951	5.4	Rv0966c*	0.74	Rv0966c			1077836	
			Rv0967	0.86	csoR			1077954	Both
**7**	1163531–1163571	50.83	Rv1040c*	4.75	PE8		Y	None	
**8**	1214391–1214431	5.08	Rv1088	Not Sig	PE9		Y	None	
**9**	1287091–1287131	60.55	Rv1161*	1.76	narG			1287124	Both
**10**	1288291–1288331	7.14	Within Rv1161	1.76	narG			N/A	
**11**	1304571–1304611	34.76	Within Rv1173	Not Sig	fbiC			N/A	
***12***	*1471651–1471691*	*5.4*	*Ribosomal RNA* f	*N/A*	*N/A*			*N/A*	
**13**	1561351–1561391	12.06	Rv1386*	0.67	PE15		Y	1561403	Strv
**14**	1728391–1728431	10.62	Rv1527c*	1.33	pks5		Y	1728877	Strv
**15**	1728911–1728951	12.52	Rv1527c*	1.33	pks5			1728877	Strv
**16**	1735631–1735671	6.45	Rv1535	0.44	Rv1535			1735507/8	Both
**17**	1744831–1744871	30.01	Rv1542c*	99.66	glbN	Y		1744836	Strv
**18**	1753431–1753471	30.04	Rv1548c*	1.14	PPE22		Y	None	
			Rv1549	0.56	fadD11			1753563	
**19**	2029771–2029811	5.38	Rv1791	1.33	PE19		Y	2029771	Both
**20**	2487551–2487591	9.56	Rv2219A*	Not Sig	Rv2219Ac			2487478	Both
			Rv2220	1.35	glnA1	Y		2487544	Exp
**21**	2493731–2493771	41.58	Rv2222c	1.23	glnA2	Y		2493745	Both
**22**	2553111–2553151	47.26	Rv2281	1.30	pitB			2553192	
**23**	2563071–2563111	9.15	Rv2291	0.63	sseB			2562953	Both
**24**	2603491–2603531	42.33	Rv2329c	2.33	narK1			2603499	Both
**25**	2752931–2752971	12.79	Within Rv2452c	Not Sig	Rv2452c		Y	N/A	
			Within Rv2453c	Not Sig	mobA		Y	N/A	
**26**	3079111–3079151	6.27	Rv2769c*	5.11	PE27		Y	3079137	
**27**	3477871–3477911	6.29	Within Rv3109	0.62	moaA1		Y	N/A	
**28**	3478231–3478271	5.47	Within Rv3109	0.62	moaA1		Y	N/A	
**29**	3595491–3595531	6.07	Rv3219	Not Sig	whiB1	Y	Y	3595603	Both
**30**	3784831–3784871	14.79	Rv3370c	Not Sig	dnaE2			3484777	
			Rv3371	24.60	Rv3371			3784902	Strv
**31**	3799991–3800031	15.45	Rv3385c*	Not Sig	vapB46			3800093	Both
			Rv3386	Not Sig	Rv3386			3799988	Both
**32**	3834631–3834671	26.47	Rv3415c	Not Sig	Rv3415c		Y	None	
			Rv3416	3.44	whiB3	Y	Y	3834791	Both
**33**	3964871–3964911	5.15	Within Rv3528c	0.85	Rv3528c		Y	N/A	
**34**	3971191–3971231	8.71	Within Rv3533c	0.91	PPE62		Y	N/A	
**35**	4060611–4060651	8.02	Rv3620c*	Not Sig	esxW		Y	4060630	
**36**	4062351–4062391	48.07	Rv3622c*	0.81	PE32		Y	None	
			Rv3623	Not Sig	lpqG		Y	4062342	Both

a Assigned peak number.

b Peak coordinates on the *M. tuberculosis* genome.

c Fold enrichment of each peak compared with the input control calculated using SISSRs.

d Adjacent gene(s) to peak (*italics* denotes intragenic).

e Fold change in gene expression (wild type vs *glnR* KO) with FDR < 0.1, Not Sig means FDR > 0.1.

f rRNA peak, excluded from further analysis.

g Whether or not part of the GlnR regulon in *M. smegmatis*.

h Overlap of GlnR and Lsr2 binding sites (lower case, *italics* denotes intragenic).

i Transcriptional start site (TSS) from (Cortes *et al*., 2013).

j TSS recorded under starvation or exponential growth (Cortes *et al*., 2013). * Genes in operons.

### Determination of the GlnR regulon during nitrogen stress

In order to identify the genes directly controlled by GlnR, and thus determine the GlnR regulon, we compared the 35 GlnR binding sites with the profile of transcripts regulated by GlnR during nitrogen limitation (Table 2). Twenty‐one GlnR binding sites corresponded to the significant (twofold cut‐off, FDR corrected *P* value of ≤ 0.05) differential expression of 10 genes, 9 genes up regulated and 1 down regulated (Table 2), indicating that GlnR functions as both an activator and repressor of transcription. GlnR binding also controlled the expression of one set of divergent genes (peak 18), although DE fall below the twofold cut‐off in both cases. Genes adjacent to 32 predicted GlnR binding sites did not show any differential expression above the twofold cut‐off during nitrogen limitation suggesting that GlnR binds silently to the genome and/or requires additional transcription factors to control expression of these genes. All GlnR peaks and associated DE genes are provided as supplementary data file (File S4).

### Analysis of the GlnR DNA binding motif

**Figure 5:**
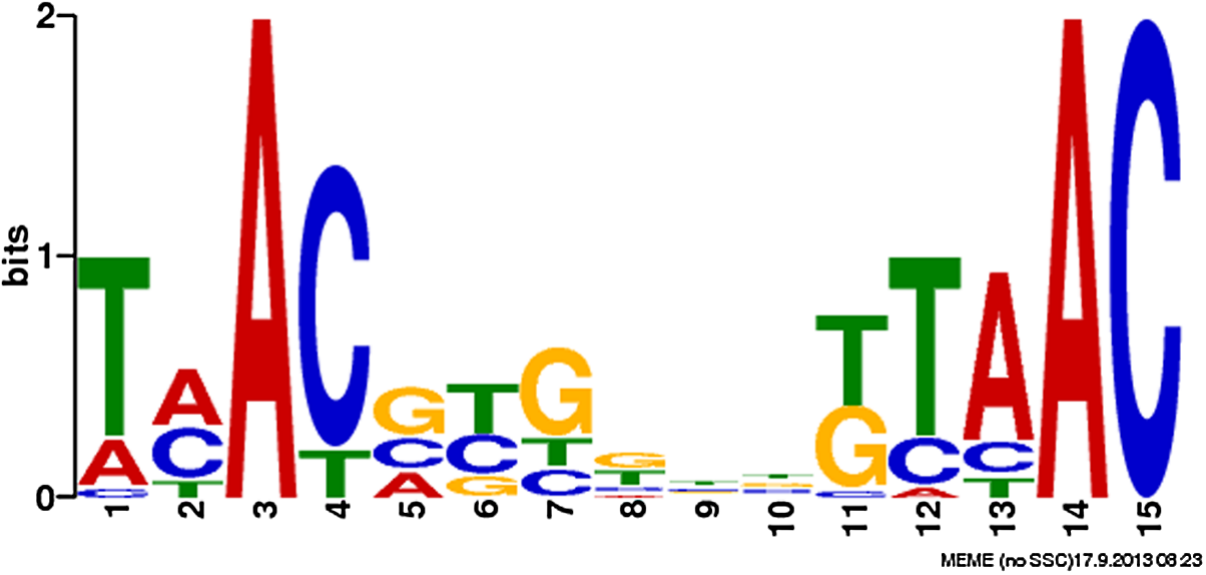
*M* *ycobacterium tuberculosis* GlnR consensus binding motif derived from 20 GlnR binding regions identified during nitrogen limitation. MEME generated GlnR motif from 200 bp DNA sequences surrounding the 20 peaks associated with differential gene expression.

Initially, all 35 GlnR binding site sequences (200 bp) identified in this study were submitted to MEME to determine if there was a motif that was responsible for GlnR genomic binding. A consensus motif was initially identified, but this motif was only present in 18 of the 35 binding sites (data not shown). Therefore, the MEME search was restricted to just GlnR binding sites in intergenic regions. With the additional constraint, a significant consensus motif was identified which was present in all GlnR regulon binding sites (Fig. 5).

### GlnR‐binding and transcriptional start sites

Of the 35 peaks identified by GlnR ChIP‐seq, 27 mapped to potential promoter regions of a total of 33 genes (including divergently transcribed) and the remaining 8 were within coding regions (*italics* in Table 2). Localisation of transcription factor binding sites within intragenic regions has been described in the case of EspR and linked to a potential additional role as a nucleoid associated protein (Galagan *et al*., 2013). It is interesting to note in this context that 6 of the 8 intragenic GlnR sites, and 15 out of 27 intergenic GlnR binding sites overlapped with sites recognised by the nucleoid associated protein Lsr2 (Gordon *et al*., 2010). One of the genes with an internal GlnR site (*moaA*) showed increased abundance in the GlnR deletion mutant, while *narG*, which has an additional promoter‐associated GlnR site, had decreased abundance. Localisation of the potential promoter‐associated GlnR sites in the context of a whole genome map of transcription start sites (TSSs) in *M. tuberculosis* (Cortes *et al*., 2013) identified adjacent TSSs for 27 of the 33 genes, including seven TSSs below the stringent cut‐off used by Cortes *et al*. that were detected by visual inspection of the original data using the Artemis genome browser. Four of the TSSs were recorded only under starvation conditions, 2 only during exponential growth, and 15 during both (Table 2). Five of the six genes for which we were unable to identify a relevant TSS also overlapped with Lsr2 sites; the remaining gene (*narK3*) was strongly down‐regulated in the GlnR knockout (Table 2). Rv1535 is flanked by two riboswitches: an Mbox upstream of Rv1535 and a T‐box upstream of Rv1536 (*ileS*) (Arnvig *et al*., 2011), but there are no transcripts evident from the intergenic probes on the tiling microarray flanking Rv1535.

## Discussion

Despite more than a century of TB research, including the publication of the genome sequence of *M. tuberculosis* in 1998, fundamental questions still remain about the biology of the organism, such as the nature of the nutrients available to the pathogen and how it obtains these from its host environment. This lack of basic knowledge about the physiology of the organism has impacted negatively on our ability to identify targets for the urgently needed new drugs. Despite this lack of success, an attractive area for TB drug development remains the discovery of drugs with novel modes of action that inhibit essential metabolic processes. In order to identify such targets, we need a better understanding of host environments and how the bacteria metabolically adapt to them. The ability to sense and respond to the environment is fundamental for bacterial survival, such responses often occur through coordinated and complex programmes of metabolite signalling molecules, signal transduction proteins, metabolic enzymes and gene expression controlled by transcriptional regulators. Although several global studies of the response of *M. tuberculosis* to various models of *in vivo* stresses have been performed (for example Voskuil, 2003; Voskuil *et al*., 2004; Voskuil *et al*., 2011; Cunningham‐Bussel and Zhang, 2013), the specific metabolic adaptation to nitrogen has not been studied, and the genes/pathways responsible for nitrogen uptake and assimilation are unknown.

The mycobacterial genome (Cole *et al*., 1998) provides insight into nitrogen metabolic pathways and nitrogen sources the tubercle bacillus can potentially use, but does not indicate which pathways are active and under what conditions. *M. tuberculosis* contains fewer genes predicted to be involved in nitrogen metabolism than the non‐pathogenic *M. smegmatis* (see Amon *et al*., 2009 for a comparison), presumably due to the diverse range of environmental nitrogen sources available in the soil compared with those within a vertebrate host. *M. tuberculosis* encodes genes for ammonium uptake and assimilation through GS/GOGAT pathways, urea and nitrate assimilation, and amino acid permeases, indicating that it may encounter these compounds *in vivo*. However, many of these nitrogen sources are toxic to host cells at high levels, limiting their availability *in vitro*. Analysis of how bacteria respond when nitrogen becomes limiting may provide more general clues about how bacteria survive *in vivo* starvation.

The identification of *glnR* in transposon screens *in* vitro and *in vivo* is rather variable. However, we would not necessarily expect to see differential expression of *glnR* – since, as described here, *glnR* is not hugely up‐regulated under nitrogen stress. Instead GlnR phosphorylation and dimer formation (signal for either unknown) modify its function rather than increased levels of GlnR. A survey of five published studies, and unpublished data from the TBdb resource, identifies two studies where there is no differential expression of *glnR* under a variety of conditions (THP‐1 infection, starvation, heat shock, Bone Marrow Macrophages (BMM), surfactants/lipids) (Fontan *et al*., 2008; Schwab *et al*., 2009). One study (Voskuil *et al*., 2004) showed a decrease in expression concomitant with the stationary phase and non‐replication persistence. Three studies (Voskuil *et al*., 2011) (Boshoff *et al*., 2004) and *Unpublished Carbon Sources* on TBdb from Liu and Schoolnik (http://www.tbdb.org/expressionHistory.shtml?gn=Rv0818) observed differential expression of *glnR* on exposure to DETA/NO, H2O2, oleic acid and palmitate.

In previous studies, we examined the transcriptomic response to nitrogen stress of the soil‐dwelling saprophyte *M. smegmatis* (Williams *et al*., 2013) and demonstrated that GlnR is the global nitrogen response regulator, controlling the expression of over 100 genes, including those involved in the assimilation and utilisation of nitrogen (Jenkins *et al*., 2013). Scavenging of nitrogen from a variety of environmental sources was a key area of metabolism up‐regulated in *M. smegmatis*, and AMBIENT analysis of the transcriptional response identified modules associated with this as well as carbon scavenging and energy generation. In this study, we used a similar approach to investigate how the obligate human pathogen *M. tuberculosis* alters its metabolism in response to nitrogen stress. The transcriptional data in Fig. S2 highlight the fact that *M. tuberculosis* does not seem to be adapted to scavenge a diverse range of nitrogen sources, with only the genes associated with asparagine and ornithine transport/metabolism upregulated greater than twofold on nitrogen run‐out at day 8.

Inspection of the AMBIENT network (Fig. 2) and comparison with the GlnR‐binding data (Table 2) show that GlnR regulation of nitrate–nitrite metabolism and nitric oxide detoxification is the major response to nitrogen stress, with both nitrate (*narGHJI*) and nitrite (*nirBD*) reductase operons activated by GlnR. The *nirBD* operon transcription is strongly activated by the intergenic GlnR‐binding site, whereas the *narGHJI* operon contains two GlnR sites, one promoter‐associated site upstream of *narG* and one within the gene (Fig. 3, peak 10), and shows only modest activation. Other genes involved in NO detoxification (*narK1*, *narK3*, *nnaR*, *glnbN*, *whiB3*) also have GlnR‐binding sites. It may be that production of nitrite by NarGHJI is limited by substrate availability, and not enzyme levels (as reflected by only modest changes in gene expression), but that as ammonium levels fall and metabolism of nitrate increases, a much larger increase in NirBD activity is needed to remove potentially toxic nitrite. GlnR does not regulate *narGHJI* in *M. smegmatis*, so the up‐regulation in response to nitrogen stress must be controlled by another mechanism, which is more in keeping with its ability to metabolise a much wider range of nitrogen sources than are evident here. The importance of nitrate–nitrite metabolism to the survival of *M. tuberculosis* inside human macrophages has recently been reported (Cunningham‐Bussel and Zhang, 2013), with bacterial adaptation to nitrite utilisation likely a key adaptation during infection, regardless of oxygen status. During adaptation to hypoxia, nitrate has also been shown to modulate the tricarboxylic acid cycle in mycobacteria (Eoh and Rhee, 2013), regulating both metabolism and respiratory activity, and the methylcitrate cycle/isocitrate lyase are up‐regulated here.

*Mycobacterium tuberculosis* also does not show the large hydrogen peroxide response seen in *M. smegmatis* (Williams *et al*., 2013), but *ahpC*, *ahpD* and *katG* are all up‐regulated in response to nitrogen stress. A similar response has been reported on exposure of mycobacteria to nitric oxide (Voskuil *et al*., 2011), leading the authors to propose that mycobacteria are continually primed for oxidative stress defence.

The reduction in bacterial growth rate after nitrogen run‐out is accompanied by a decrease in ribosomal proteins, and the AMBIENT shows how alterations (up and down) in metabolism are linked. For example, Rv2928 (fatty acyl ACP hydrolase) and Rv2930 (fatty acyl CoA ligase) both act on phthiocerol and are down‐ and up‐regulated respectively. Aspartate metabolism, which has been proposed to be an important source of nitrogen *in vivo* (Gouzy *et al*., 2013), is altered with less going to pyrimidine biosynthesis, making more aspartate available to be converted into ammonium. Genes down‐regulated in reduced growth rate may not be associated with nitrogen stress *per se*, but up‐regulated genes are going against the general metabolism down shift so are likely to be part of the nitrogen stress response. RNA was collected when external nitrogen had run out to try and mitigate the effect of the subsequent decrease in growth rate.

Several AMBIENT nodes are marked as ‘none’ in Fig. 2; these are either spontaneous chemical reactions or are indicated as a result of the manual curation of the genome model, suggesting that such a reaction exists, but has not yet been identified.

The GlnR‐regulon in *M. tuberculosis* is much more restricted in terms of the number of GlnR‐binding sites, with only 26 intergenic ones (Table 1), compared with 53 in *M. smegmatis* (Jenkins *et al*., 2013). This fits with the smaller number of nitrogen metabolism genes compared with *M. smegmatis*. Indeed there are only eight binding site locations shared between the two organisms in terms of the genes controlled. The GlnR‐binding site identified is similar to that found for *M. smegmatis* with conserved AC residues nine bases apart. As mentioned earlier, *nirB* is GlnR controlled in both, as are genes associated with nitrogen metabolism such as the nitrate/nitrite transporter *narK3* and the transcription factor *nnaR*. The two glutamine synthetases are GlnR regulated in both organisms, although only *glnA1* is differentially expressed in *M. tuberculosis*. Two other transcription factors, *whiB1* and *whiB3*, feature in both organisms, as does the most highly up‐regulated gene the globin *glbN*. This encodes a potent mannosylated oxygen‐dependent nitric oxide dioxygenase which protects mycobacteria from toxic NO generated by macrophages (Arya *et al*., 2013). Its up‐regulation under *in vitro* conditions may be in response to NO produced as by‐product of nitrate reductase activity. Overall this suggests a central core of genes in both organisms that mediate the bacterial response to nitrogen stress – not only directly through the nitrite reductases and transporters but also indirectly via other transcription factors, which may recruit more general stress response elements, such hydrogen peroxide detoxification (Voskuil *et al*., 2011) needed for survival. A recent paper (Elharar *et al*., 2014) shows that survival of *M. smegmatis* under nutrient limitation, including nitrogen, requires proteasome‐mediated amino acid recycling.

Of the 28 genes for which we identified a TSS, in 18 cases, the midpoint of GlnR‐binding fell within a region between 75 bp upstream and 10 bp downstream of the TSS that is commonly associated with transcription factor regulation. For 11 of these genes, the level of expression in the GlnR knockout was significantly decreased over that in the wild type (Table 2), consistent with a model in which GlnR binding acts by promoting access of RNA polymerase. GlnR homo‐dimer formation is important in its function, and its interaction with other proteins may be governed by post‐translational modifications such as acetylation or small molecule binding (Lin *et al*., 2014). Any post‐translational modification and interaction with other factors may explain its dual ability to both repress and activate gene expression.

The expression of glutamine synthase genes *glnA1* and *glnA2*, which have previously been identified as components of the GlnR regulon in *M. smegmatis*, showed little or no change in expression in the knockout. While the overall abundance of *gln*A1 and *gln*A2 transcripts also changed only slightly in the starvation model described by Cortes *et al*., they recorded a switch in the dominant TSS from a distal to a proximal site in the case of *glnA1* and from a leaderless to an internal TSS for *glnA2*. The location of GlnR binding sites at positions −37 (*glnA1*) and −30 (*glnA2*) would be consistent with GlnR regulation of transcription from the downstream promoter in each case. The resulting change in 5′ untranslated region may affect the efficiency of translation. A similar switch from distal to proximal TSS occurs in the starvation response of thiosulphate sulphuryltransferase *sseB*, with an overall increase in transcript abundance in the GlnR knockout. In spite of GlnR binding at −23 with respect to the TSS, there was no change in the level of *csoR* transcript in the knockout. In this case, the GlnR binding site overlaps that of CsoR itself, which inhibits its own expression under conditions of low copper concentrations (Liu *et al*., 2007). EsxW, part of the new TB vaccine (Knudsen *et al*., 2014), shows a similar picture, with a GlnR‐binding site which overlaps with that of Lsr2 (Gordon *et al*., 2010) and no differential expression. In this case, the lack of altered expression also fits with transcriptional data obtained during mouse infection (Knudsen *et al*., 2014), used as evidence for its stability and suitability as a vaccine candidate. Transcription may be dually regulated by competition between the two transcription factors in these cases. Similarly for PE15, transcriptional regulation may depend on competition between GlnR and cAMP receptor protein binding to a site at 1561338 (Bai *et al*., 2007), and for *whiB1*, there may be competition between CRP (Rickman *et al*., 2005; Agarwal *et al*., 2009), Lsr2 (Gordon *et al*., 2010) and GlnR, suggesting a complex level of regulation.

With the evolution of *M. tuberculosis* as an obligate vertebrate pathogen, it has lost many of the pathways not required for the assimilation of nutrients readily available *in vitro*. In keeping with this, the GlnR‐regulon has shrunk overall, with the nitrogen component now seemingly limited to the nitrate and nitrite reductases, which are presumably a main source of nitrogen available in the host. We still do not know how GlnR is activated. There is no obvious pattern linking the GlnR‐binding site, the transcriptional start site and whether GlnR functions as an activator or repressor. Presumably, this depends on post‐translational modification or the interaction with other transcription factors and DNA binding proteins, and indeed the majority of genes that show no differential expression have GlnR‐binding sites that overlap with Lsr2, CRP or both. This suggests that regulation results from a complex interplay between these factors, or that some sites act as depots for DNA‐binding proteins. The GlnR regulated inter conversion of nitrate, nitrite and nitric oxide are increasingly recognised as the key processes in nitrogen assimilation and intracellular survival by *M. tuberculosis* (Voskuil *et al*., 2011; Cossu *et al*., 2013; Cunningham‐Bussel and Zhang, 2013; Eoh and Rhee, 2013), making them important areas for further research.

## Experimental procedures

### Bacterial strains and growth conditions

*Mycobacterium tuberculosis* H37Rv wild type (ATCC 27294) and *M. tuberculosis* H37Rv *glnR* KO (this study) were used in this study. *M. tuberculosis* was grown aerobically in Middlebrook 7H9 liquid broth (supplemented with 0.2% glycerol, 0.025% tyloxapol and 10% OADC) at 37°C, 100 rpm. Optimised nitrogen limiting conditions have been described (Behrends *et al*., 2012; Jenkins *et al*., 2012). Briefly, a 10 day culture of *M. tuberculosis* was washed twice in nitrogen‐free Sauton's medium (0.05% (w/v) KH_2_PO_4_, 0.05% (w/v) MgSO_4_, 0.2% (w/v) citric acid, 0.005% (w/v) ferric citrate, 0.2% (v/v) glycerol, 0.0001% (v/v) ZnSO_4_, 0.015% (v/v) tyloxapol) and inoculated into Sauton's nitrogen‐free medium, supplemented with 1 mM (nitrogen limiting) or 30 mM (nitrogen excess) ammonium chloride (Ultra pure; Sigma) to a starting OD_60nm_ of 0.04. Growth was monitored by OD_600nm_. Ammonium ions in the culture supernatant were quantified using an AquaQuant Ammonium detection kit (Merck).

### Construction of GlnR mutant

The *M. tuberculosis glnR* KO mutant was constructed by a recombineering approach as previously described (van Kessel and Hatfull, 2007), replacing the entire *glnR* gene with a hygromycin resistance cassette (Jenkins *et al*., 2012). Primers used to amplify the flanking regions and to confirm mutant construction are given in Table S1. Successful construction of the mutant was confirmed by flanking PCR and Western analysis for the GlnR protein using a GlnR specific antibody previously described (Jenkins *et al*., 2013). For complementation experiments, vector pSM57, a 1582 bp *Eco*RV–*Sma*I fragment containing *glnR* cloned into the promoterless pMV306 vector (kind gift from Prof Franz‐Christoph Bange) (Malm *et al*., 2009), was transformed into the *glnR* mutant.

### EMSA

To analyse GlnR binding to gene promoter regions, DNA fragments were PCR amplified from *M. tuberculosis* genomic DNA and used in EMSA. Primers used to amplify DNA regions are given in Table S1. His‐GlnR protein was purified as described (Jenkins *et al*., 2013). DNA fragments were labelled using a DIG Oligonucleotide 3′‐End Labelling Kit (Roche). DNA : protein binding reactions contained 0.4 ng of labelled DNA, 0.5 μg poly d(A–T), 0–0.9 μg His‐GlnR, 25 mM HEPES (pH 7.9), 150 mM NaCl, 2.5 mM MgCl_2_. The reaction mixture was incubated at 37°C for 15 min, before separation on a pre‐run 6% DNA retardation gel (Life Technologies). Bands were visualised using a LAS‐3000 Fuji imager.

### Rate‐limiting PCR (qPCR)

To identify enrichment in GlnR‐immunoprecipitated DNA, a rate‐limiting PCR was performed. DNA was immunoprecipitated and purified as described under ChIP. DNA sequences were amplified using primers listed in Table S1. Reaction mixtures consisted of GlnR‐immunoprecipitated DNA (0.3 ng), 1 × BioMix (Bioline), 1 μM of each primer and 5% (v/v) dimethyl sulfoxide (Sigma). PCR was carried out in a thermocycler T3000 (Biometra); 95°C for 5 min, 23 cycles of 95°C 30 s, 55°C 30 s, 72°C 1 min, with final extension 72°C for 8 min. DNA was visualised on a 2% agarose gel.

### RNA isolation

*Mycobacterium tuberculosis* strains were grown in triplicate in nitrogen limiting conditions until external nitrogen was depleted. Total RNA was extracted from exponentially growing cells using the GTC/Trizol method (Ehrt *et al*., 2003). Extracted RNA was purified using the RNeasy kit (Qiagen) and residual DNA was removed by TURBO DNA‐free (Ambion Life Technologies) treatment. Superase (ABI Life Technologies) was added and RNA was stored at −80°C. Quality and quantity of RNA were determined using a Bio‐analyser (Agilent).

### Quantitative real‐time PCR (qRT‐PCR)

cDNA was amplified from 100 ng of RNA using the SuperScript III First‐Strand Synthesis SuperMix (Invitrogen). qRT‐PCR reactions were carried out in a final volume of 10 μl [1 μl of cDNA, 5 μl of TaqMan PCR master mix (Applied Biosystems), 0.5 μl TaqMan probe (Applied Biosystems)]. Amplification was performed on an Applied Biosystems 7500 Real‐Time System (50°C 5 min, 95°C 10 min, and 40 cycles of 95°C 15 s, 60°C 1 min). Linear amplification and amplification efficiencies for each TaqMan primer/probe were determined. Real‐time analysis was performed on RNA from three independent cultures and quantification of *sigA* expression served as an internal control. Fold change was calculated as a ratio of the arbitrary expression units, standardised to *sigA*. Primers and Taqman probe sequences for each gene studied are given in Table S2.

### Preparation of labelled cDNA from total RNA

Labelled cDNA was prepared from 1 μg of total RNA using Cy3‐dCTP (GE Healthcare) and SuperScript II reverse transcriptase with random hexamer primers (*Invitrogen*). Labelled cDNA was purified by Qiagen MinElute column, combined with 10 × CGH blocking agent and 2× Hi‐RPM hybridisation buffer (Agilent) and heated (95°C for 5 min) prior to loading onto microarray slides. Slides were incubated overnight in an Agilent rotating oven at 65°C, 20 rpm. After hybridization, slides were washed (5 min at room temperature) with CGH Wash Buffer 1 (Agilent) and 1 min at 37°C with CGH Wash buffer 2 (Agilent). Slides were scanned immediately, using an Agilent High Resolution Microarray Scanner (Agilent Technologies, Stockport, UK), at 2 μm resolution, 100% PMT. Scanned images were quantified using Feature Extraction software v 10.7.3.1. RNA for tiling microarray hybridization was directly labelled using the Kreatech Universal Linkage System (Leica Microsystems, Milton Keynes, UK), which is based on the stable binding of platinum to the N7 position of guanine, according to the manufacturer's instructions.

### Microarray design

The microarray for the wild type vs GlnR KO experiments was constructed by determining all unique genes from the 3992 chromosomal predicted coding sequences of *M. tuberculosis* H37Rv, downloaded from Ensembl Bacteria Release 5 (http://bacteria.ensembl.org/). Multiple optimal hybridisation 60‐mer oligonucleotide sequences were designed (Oxford Gene Technologies), from which a minimal non‐redundant subset of oligonucleotides were selected with target coverage of three 60‐mers per gene. Arrays were manufactured on the Inkjet *in situ* synthesized platform (Agilent) using the 8 × 60k format. Analysis of gene expression over nitrogen run‐out was performed using a 180k Tiling array, designed as described (Golby *et al*., 2013).

### Statistical analyses of differential gene expression

Statistical analyses of the gene expression data were carried out using the statistical analysis software environment R together with packages available as part of the Bioconductor project (http://www.bioconductor.org). Data generated from the Agilent Feature Extraction software for each sample were imported into R. Replicate probes were mean summarised and quantile normalised using the pre‐process Core R package. The limma R package (Smyth, 2004) was used to compute empirical Bayes moderated *t*‐statistics to identify DE genes between time points. Generated *P*‐values were corrected for multiple testing using the Benjamini and Hochberg FDR. An FDR corrected *P*‐value cut‐off of less than 0.1 was used to determine significant differential expression, which is more stringent than an uncorrected *P*‐value of 0.05.

### ChIP

*Mycobacterium tuberculosis* was grown as specified and processed for ChIP using purified rabbit anti‐GlnR polyclonal antibody as described (Jenkins *et al*., 2013). DNA were prepared for next generation sequencing using the Illumina ChIP‐seq DNA sample preparation kit according to the manufacturer's protocol, with the addition of a second gel extraction step after PCR amplification, to remove excess primer dimers. DNA size and purity were confirmed by DNA Bioanalyser (Agilent) and sequencing conducted on an Illumina HiSeq2000 sequencer (MRC Clinical Sciences Centre, Hammersmith). Sequencing reads were mapped to the *M. tuberculosis* genome using Bowtie (Langmead *et al*., 2009), and GlnR binding regions were identified using the peak‐calling algorithm SISSRs (Site Identification for Short Sequence Reads) (Narlikar and Jothi, 2012), with peaks defined as significant if they showed greater than fivefold enrichment in the immunoprecipitated sample compared with the input control DNA with a *P* value of < 0.005. The SISSR peaks and gene expression data were visualised using the Integrative Genome Viewer (IGV) (Robinson *et al*., 2011; Thorvaldsdóttir *et al*., 2013) and screenshots were taken of individual peaks.

## Supporting information

The Tiling array design is available in BμG@Sbase (Accession No. A‐BUGS‐47; http://bugs.sgul.ac.uk/A‐BUGS‐47) and also ArrayExpress (Accession No. A‐BUGS‐47). All sequencing data have been deposited in ArrayExpress (Accession No. E‐MTAB‐2492). The other data sets supporting the results of this article are included within the article and its additional files.

## Supporting information

Supporting information
