# Supplementary material for: Deciphering the metabolic response of M ycobacterium tuberculosis to nitrogen stress
Source: Mol Microbiol. 2015 Jul 17;97(6):1142–57. doi: 10.1111/mmi.13091 (PMC4950008; doi:10.1111/mmi.13091)
Supplement: Supplementary file 1 — Supporting information [file MMI-97-1142-s001.zip › MMI_13091_supp-0004-File_S4_IGV_screenshots_GlnR.pdf]

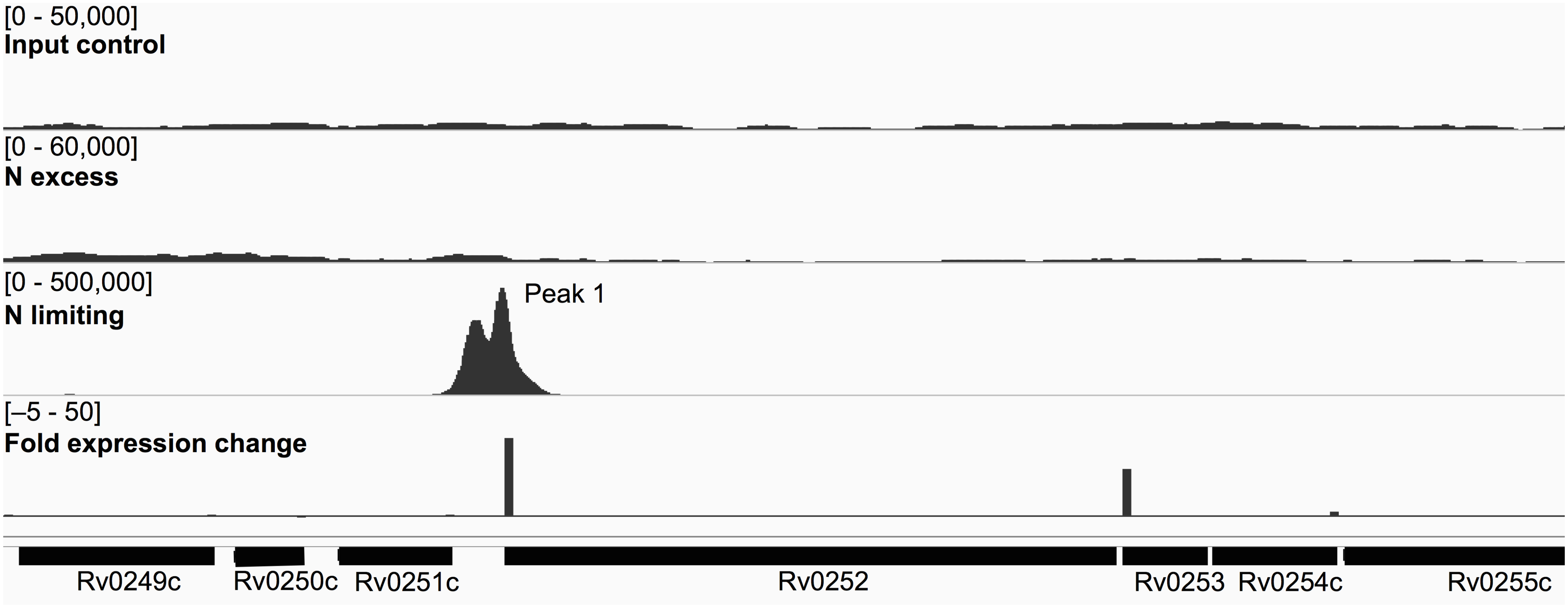
[0 - 50,000]
Input control
[0 - 60,000]
N excess
[0 - 500,000]
N limiting
Peak 1
[–5 - 50]
Fold expression change
Rv0249c
Rv0250c
Rv0251c
Rv0252
Rv0253
Rv0254c
Rv0255c

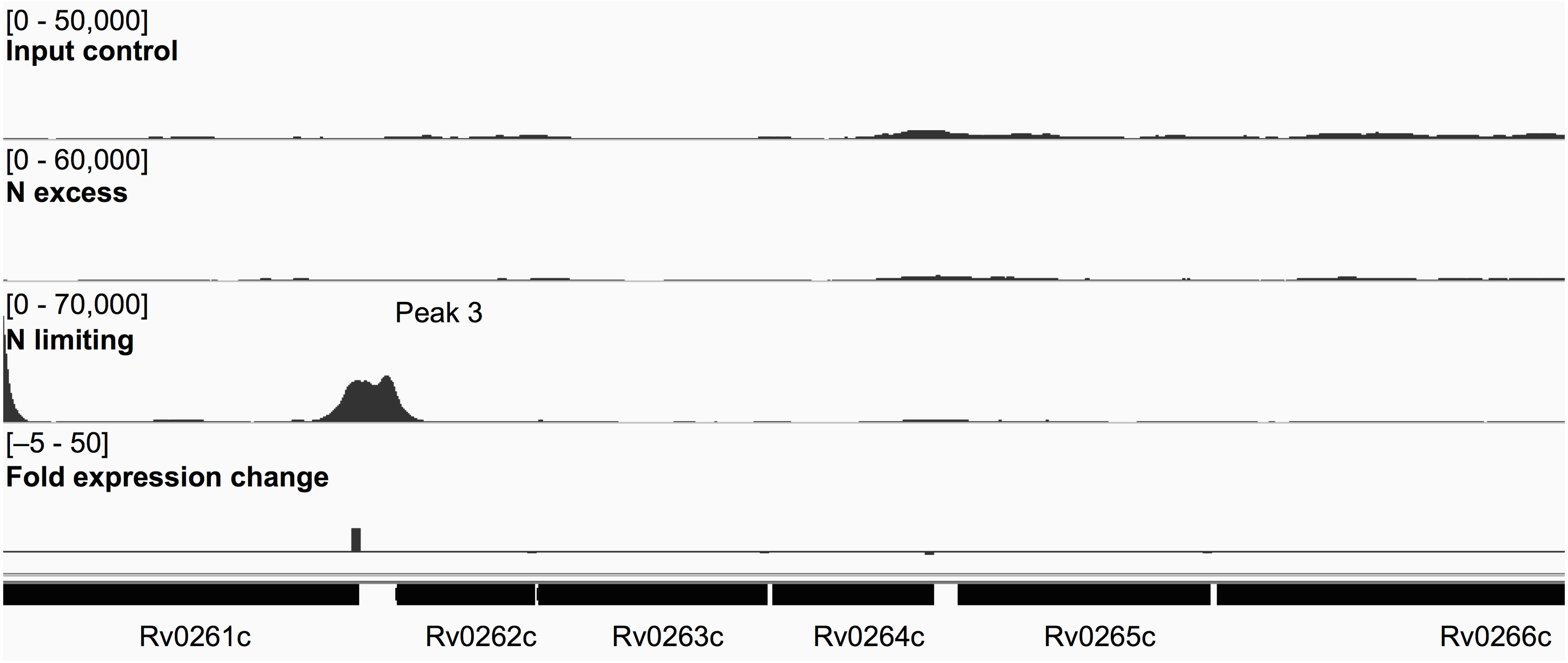
[0 - 50,000]
Input control
[0 - 60,000]
N excess
[0 - 70,000]
N limiting
Peak 3
[–5 - 50]
Fold expression change
Rv0261c
Rv0262c
Rv0263c
Rv0264c
Rv0265c
Rv0266c

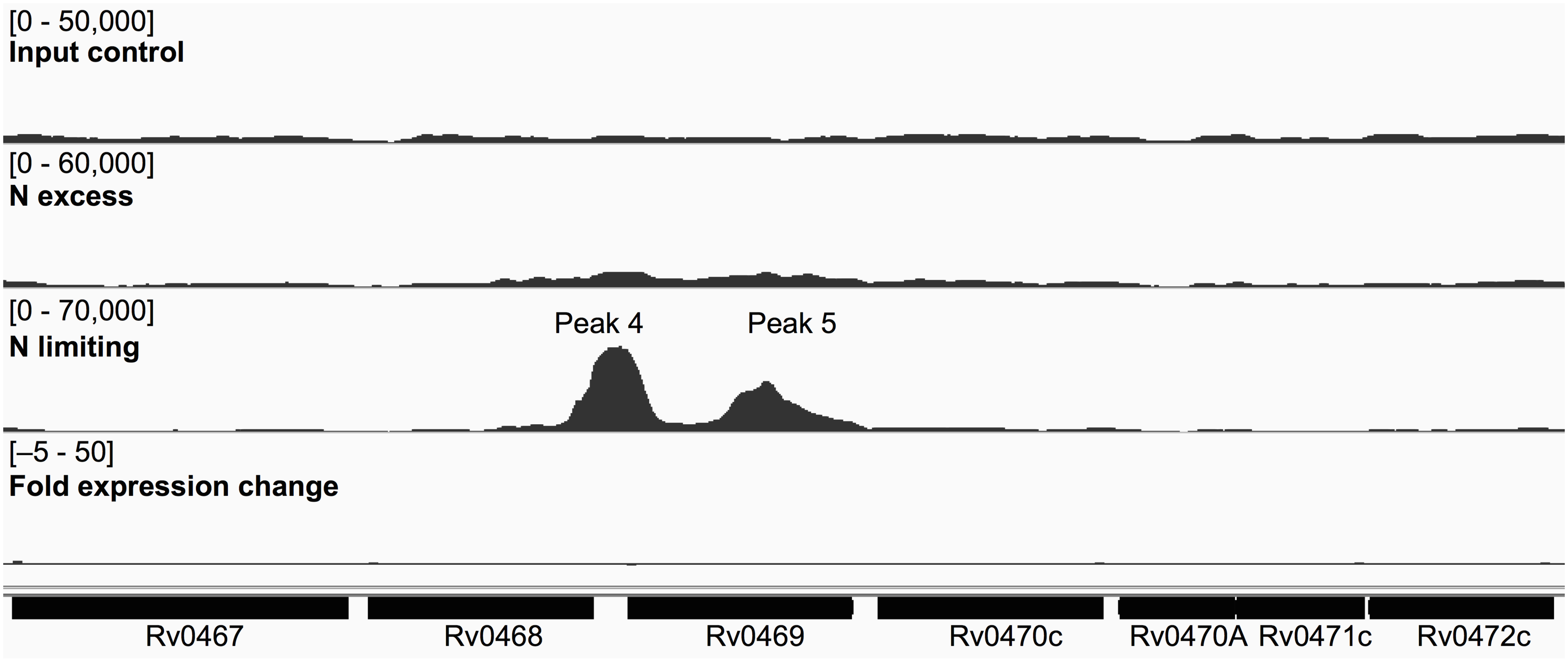
[0 - 50,000]
Input control
[0 - 60,000]
N excess
[0 - 70,000]
N limiting
Peak 4
Peak 5
[–5 - 50]
Fold expression change
Rv0467
Rv0468
Rv0469
Rv0470c
Rv0470A
Rv0471c
Rv0472c

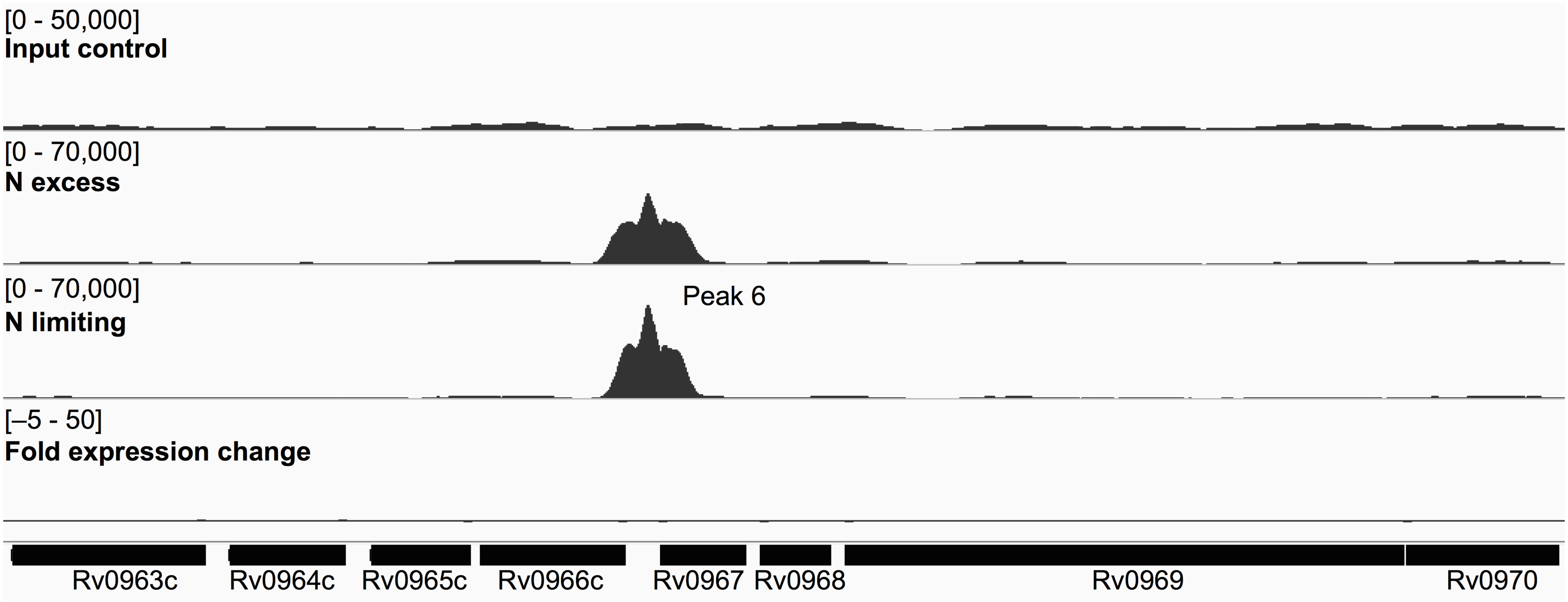
[0 - 50,000]
Input control
[0 - 70,000]
N excess
[0 - 70,000]
N limiting
Peak 6
[–5 - 50]
Fold expression change
Rv0963c
Rv0964c
Rv0965c
Rv0966c
Rv0967
Rv0968
Rv0969
Rv0970

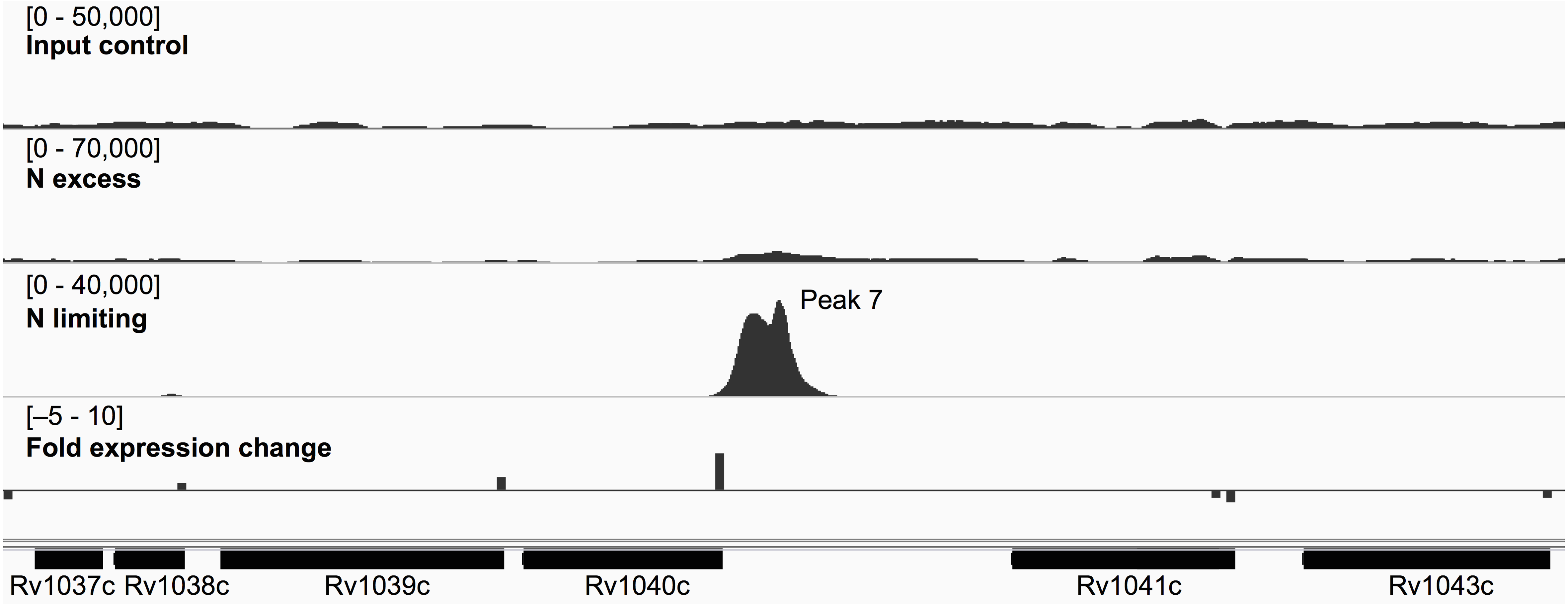

[0 - 50,000]
Input control
[0 - 70,000]
N excess
[0 - 40,000]
N limiting
Peak 7
[–5 - 10]
Fold expression change
Rv1037c
Rv1038c
Rv1039c
Rv1040c
Rv1041c
Rv1043c

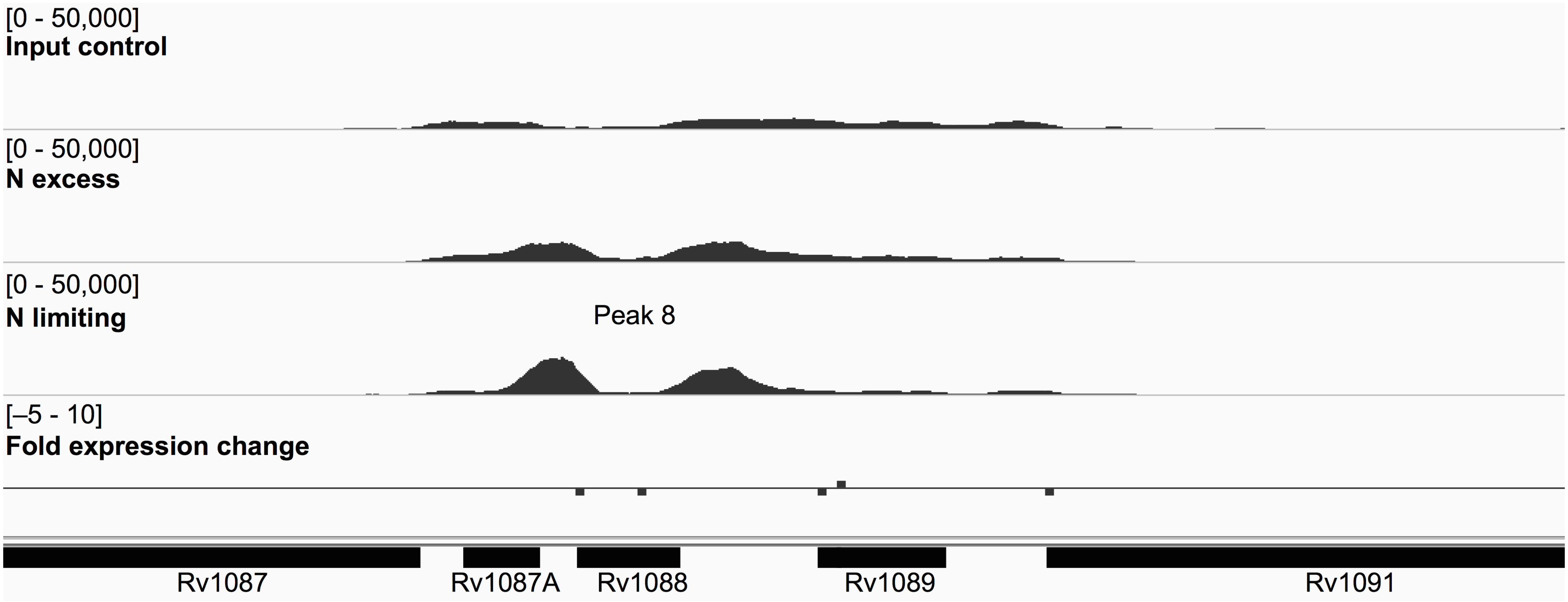
[0 - 50,000]
Input control
[0 - 50,000]
N excess
[0 - 50,000]
N limiting
Peak 8
[–5 - 10]
Fold expression change
Rv1087
Rv1087A
Rv1088
Rv1089
Rv1091

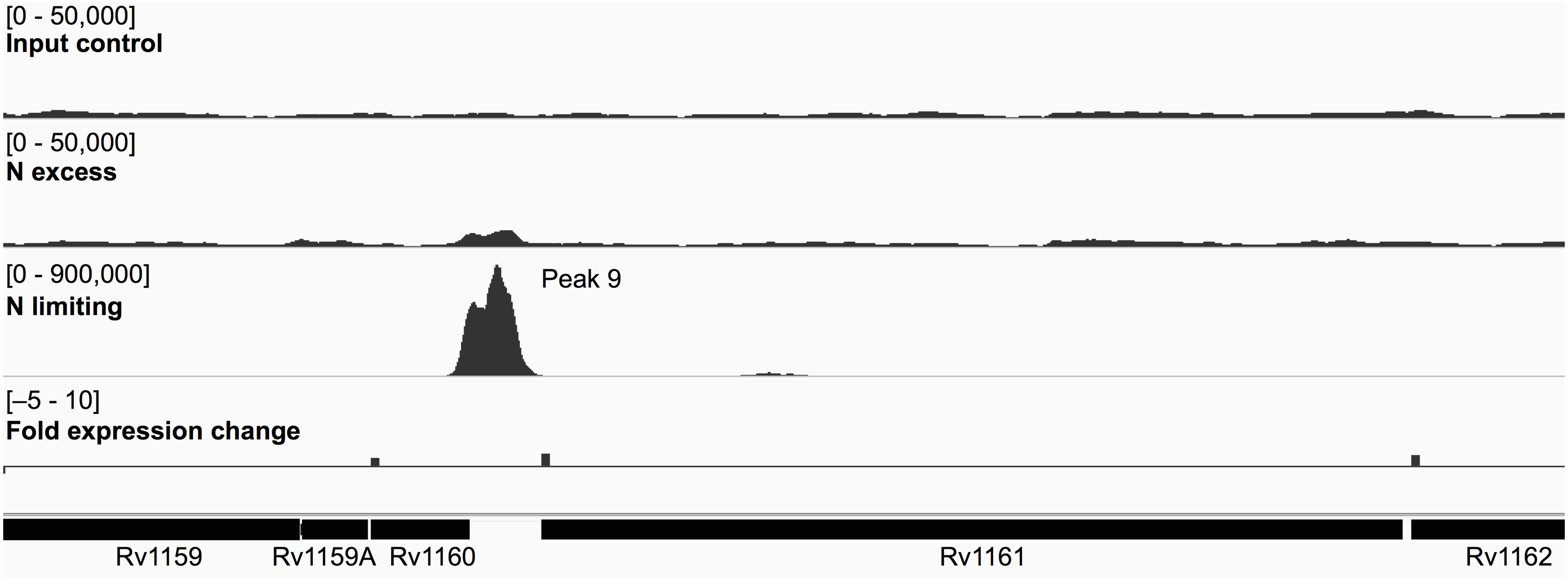
[0 - 50,000]
Input control
[0 - 50,000]
N excess
[0 - 900,000]
N limiting
Peak 9
[–5 - 10]
Fold expression change
Rv1159
Rv1159A
Rv1160
Rv1161
Rv1162

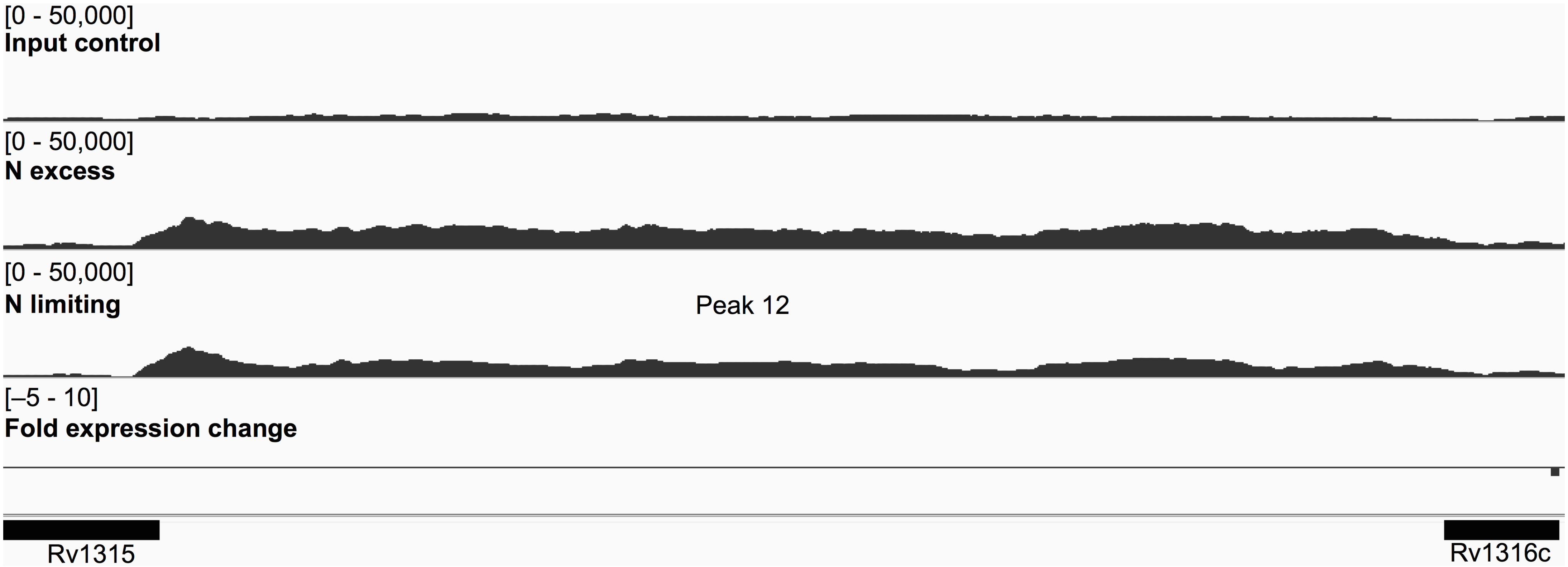
[0 - 50,000]
Input control
[0 - 50,000]
N excess
[0 - 50,000]
N limiting
Peak 12
[–5 - 10]
Fold expression change
Rv1315
Rv1316c

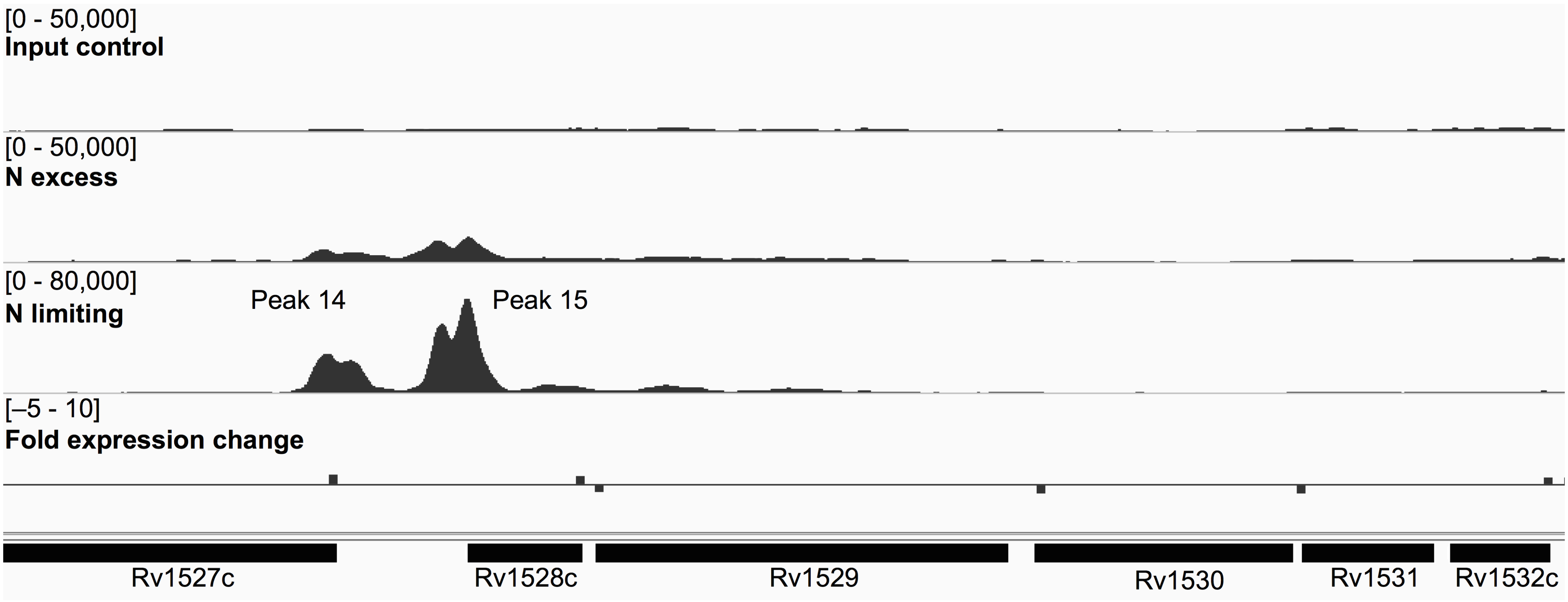
[0 - 50,000]
Input control
[0 - 50,000]
N excess
[0 - 80,000]
N limiting
Peak 14
Peak 15
[–5 - 10]
Fold expression change
Rv1527c
Rv1528c
Rv1529
Rv1530
Rv1531
Rv1532c

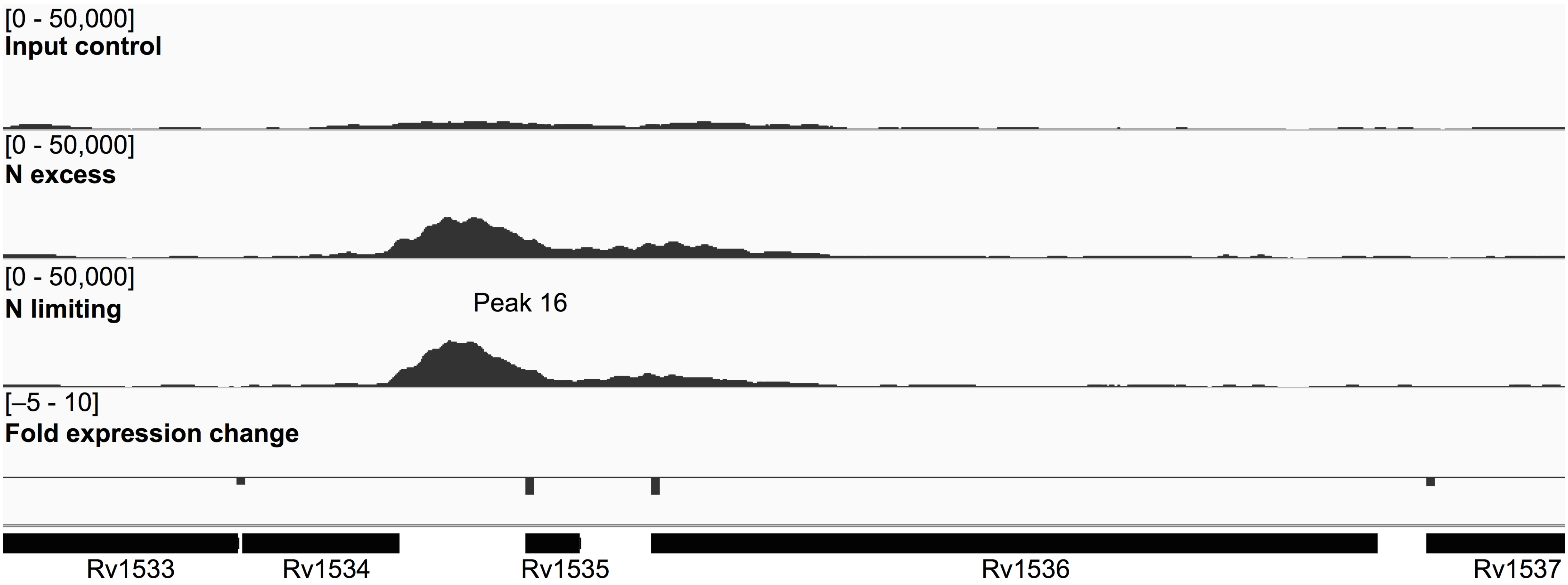
[0 - 50,000]
Input control
[0 - 50,000]
N excess
[0 - 50,000]
N limiting
Peak 16
[–5 - 10]
Fold expression change
Rv1533
Rv1534
Rv1535
Rv1536
Rv1537

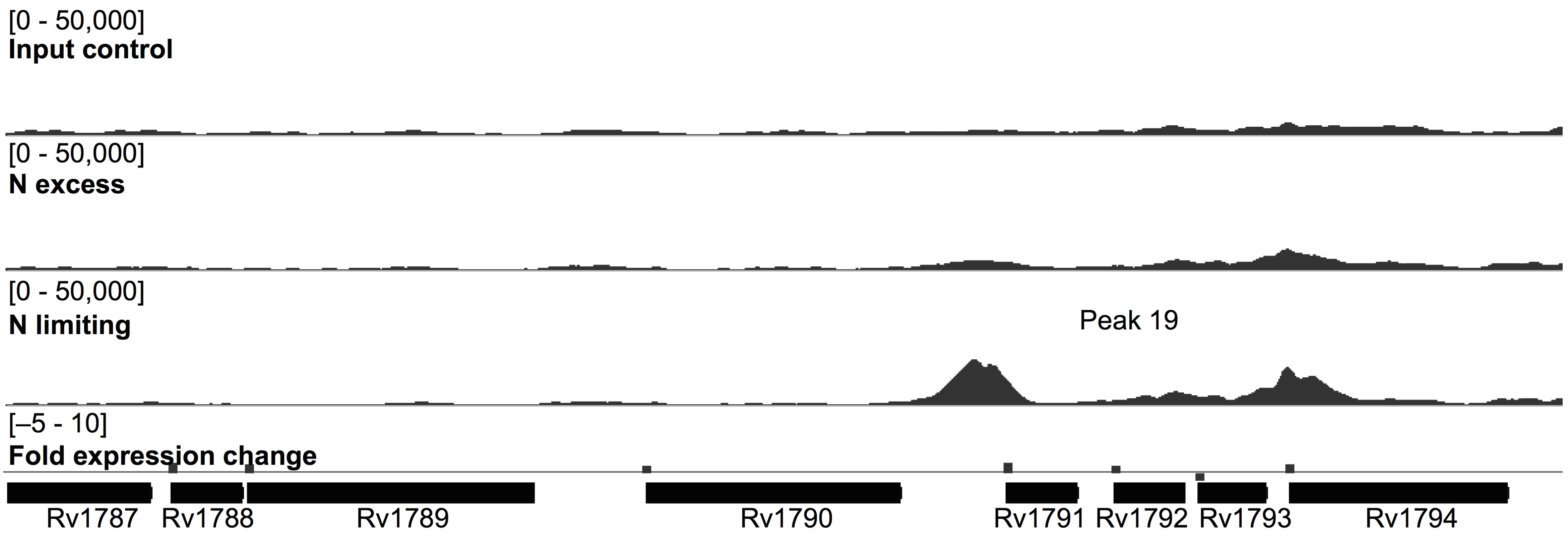
[0 - 50,000]
Input control
[0 - 50,000]
N excess
[0 - 50,000]
N limiting
Peak 19
[–5 - 10]
Fold expression change
Rv1787
Rv1788
Rv1789
Rv1790
Rv1791
Rv1792
Rv1793
Rv1794

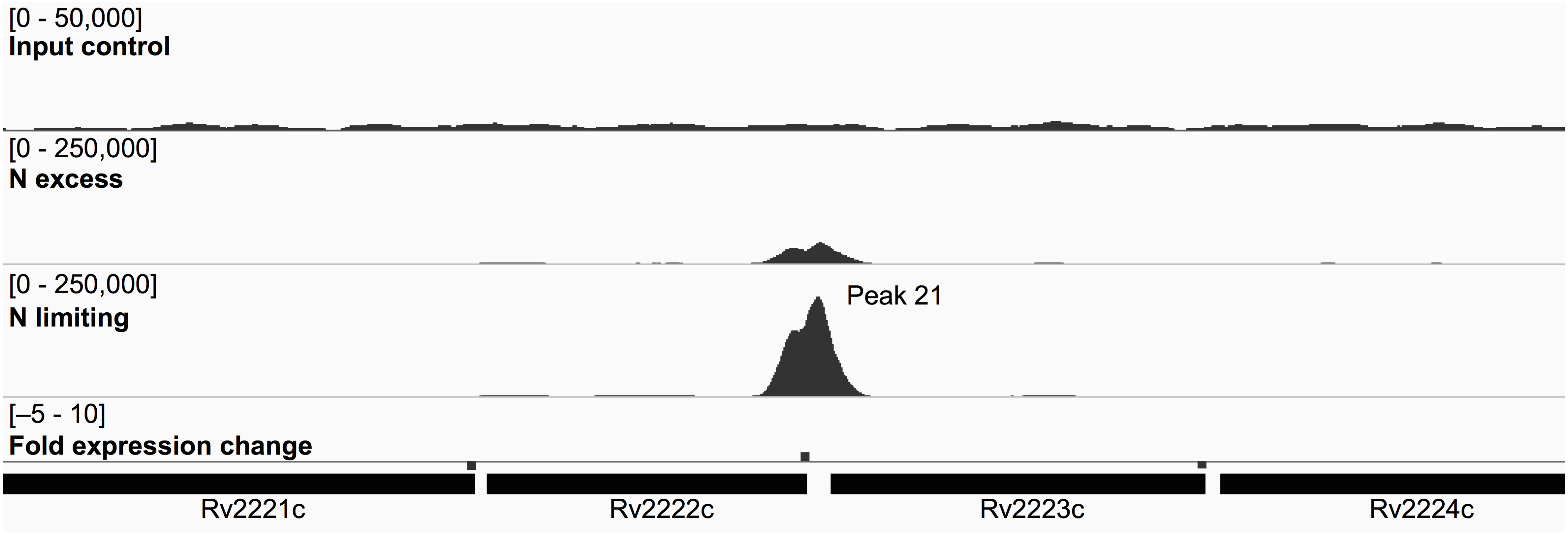
[0 - 50,000]
Input control
[0 - 250,000]
N excess
[0 - 250,000]
N limiting
Peak 21
[–5 - 10]
Fold expression change
Rv2221c
Rv2222c
Rv2223c
Rv2224c

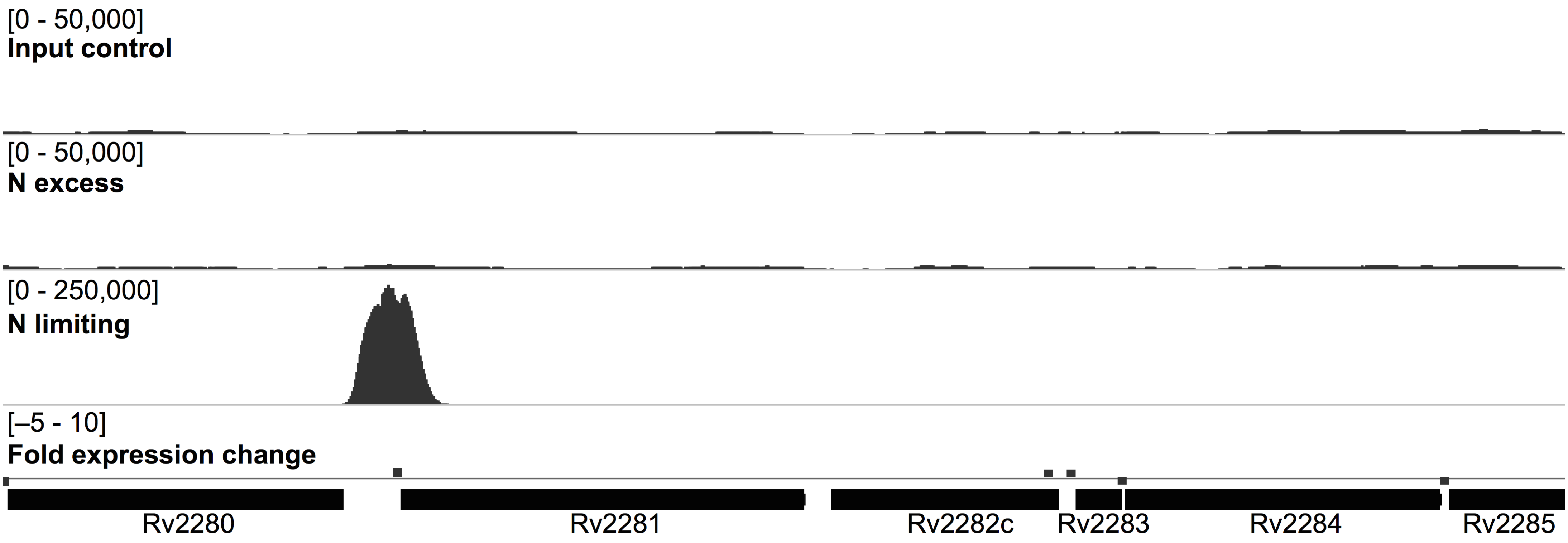
[0 - 50,000]
Input control
[0 - 50,000]
N excess
[0 - 250,000]
N limiting
[–5 - 10]
Fold expression change
Rv2280
Rv2281
Rv2282c
Rv2283
Rv2284
Rv2285

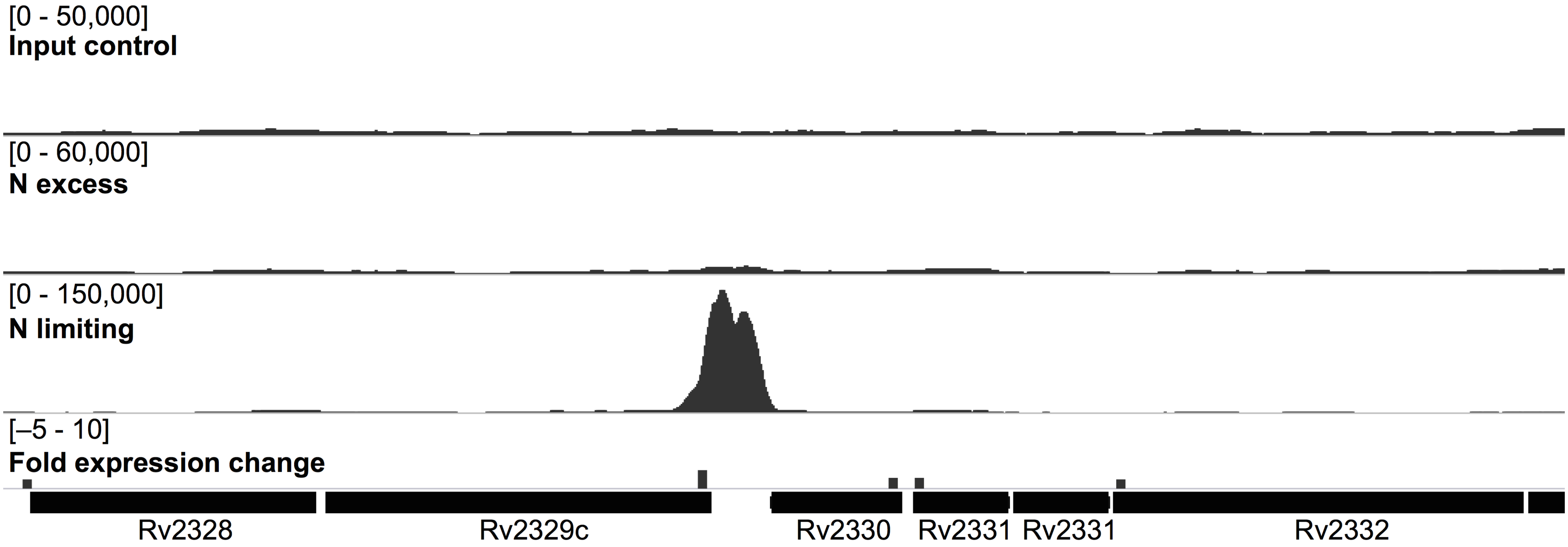
[0 - 50,000]
Input control
[0 - 60,000]
N excess
[0 - 150,000]
N limiting
[–5 - 10]
Fold expression change
Rv2328
Rv2329c
Rv2330
Rv2331
Rv2331
Rv2332

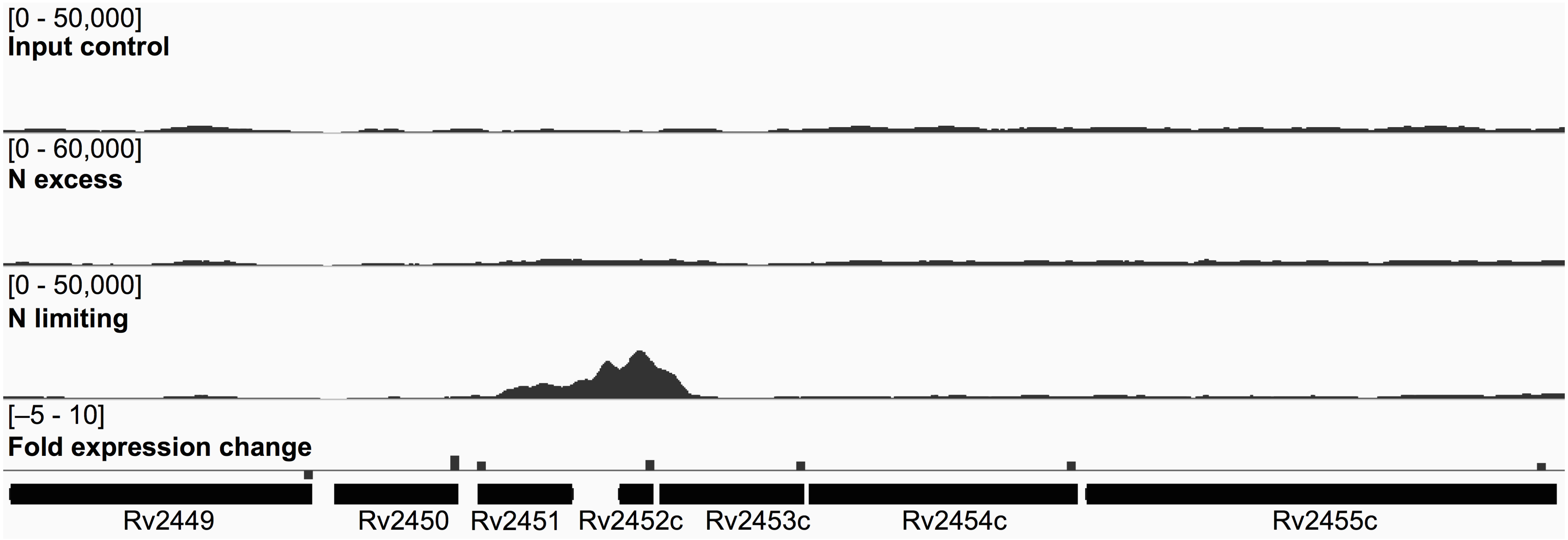
[0 - 50,000]
Input control
[0 - 60,000]
N excess
[0 - 50,000]
N limiting
[–5 - 10]
Fold expression change
Rv2449
Rv2450
Rv2451
Rv2452c
Rv2453c
Rv2454c
Rv2455c

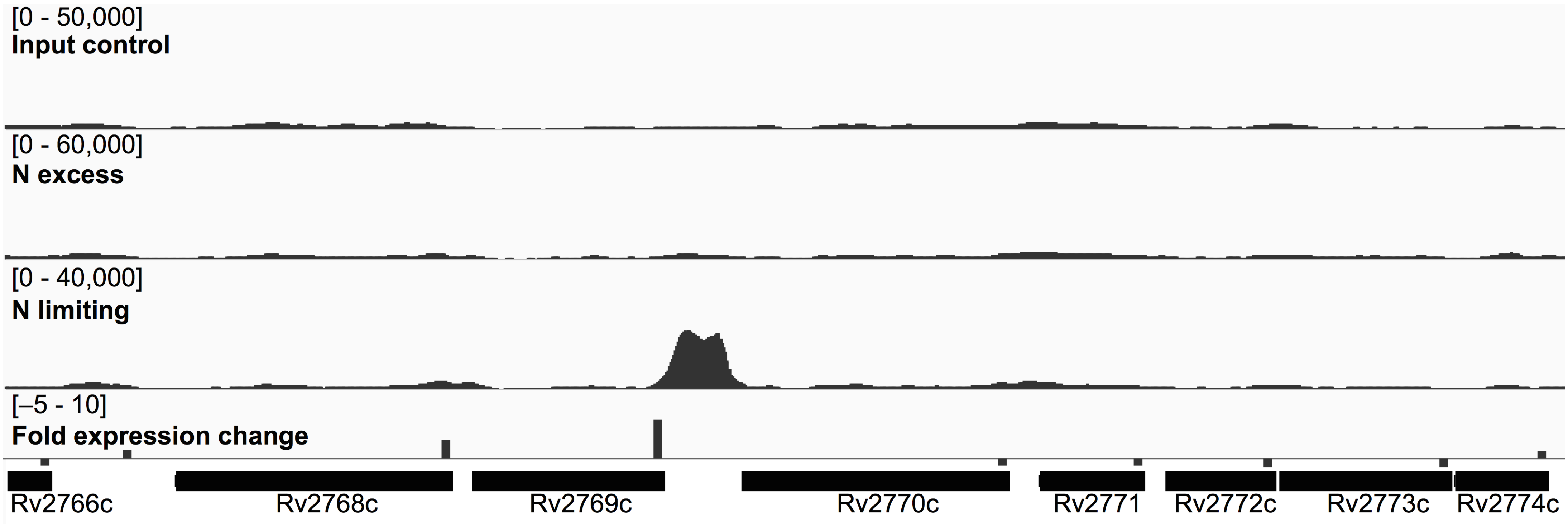
[0 - 50,000]
Input control
[0 - 60,000]
N excess
[0 - 40,000]
N limiting
[–5 - 10]
Fold expression change
Rv2766c
Rv2768c
Rv2769c
Rv2770c
Rv2771
Rv2772c
Rv2773c
Rv2774c

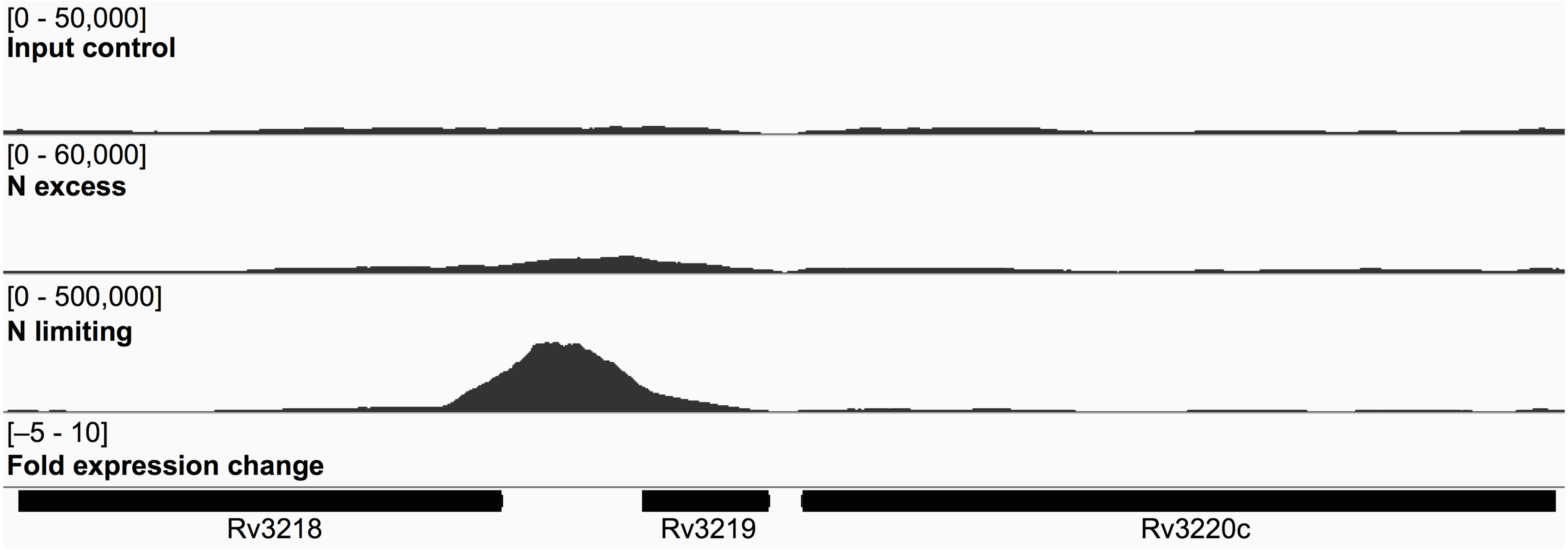
[0 - 50,000]
Input control
[0 - 60,000]
N excess
[0 - 500,000]
N limiting
[–5 - 10]
Fold expression change
Rv3218
Rv3219
Rv3220c

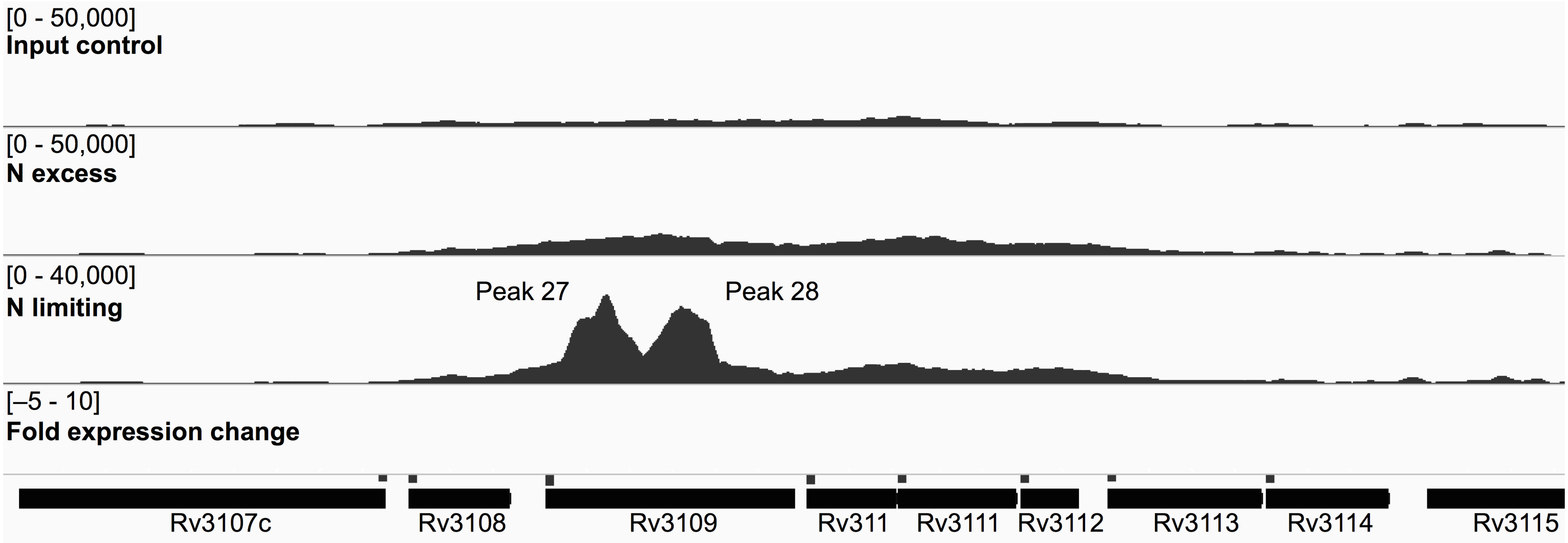
[0 - 50,000]
Input control
[0 - 50,000]
N excess
[0 - 40,000]
N limiting
Peak 27
Peak 28
[–5 - 10]
Fold expression change
Rv3107c
Rv3108
Rv3109
Rv311
Rv3111
Rv3112
Rv3113
Rv3114
Rv3115

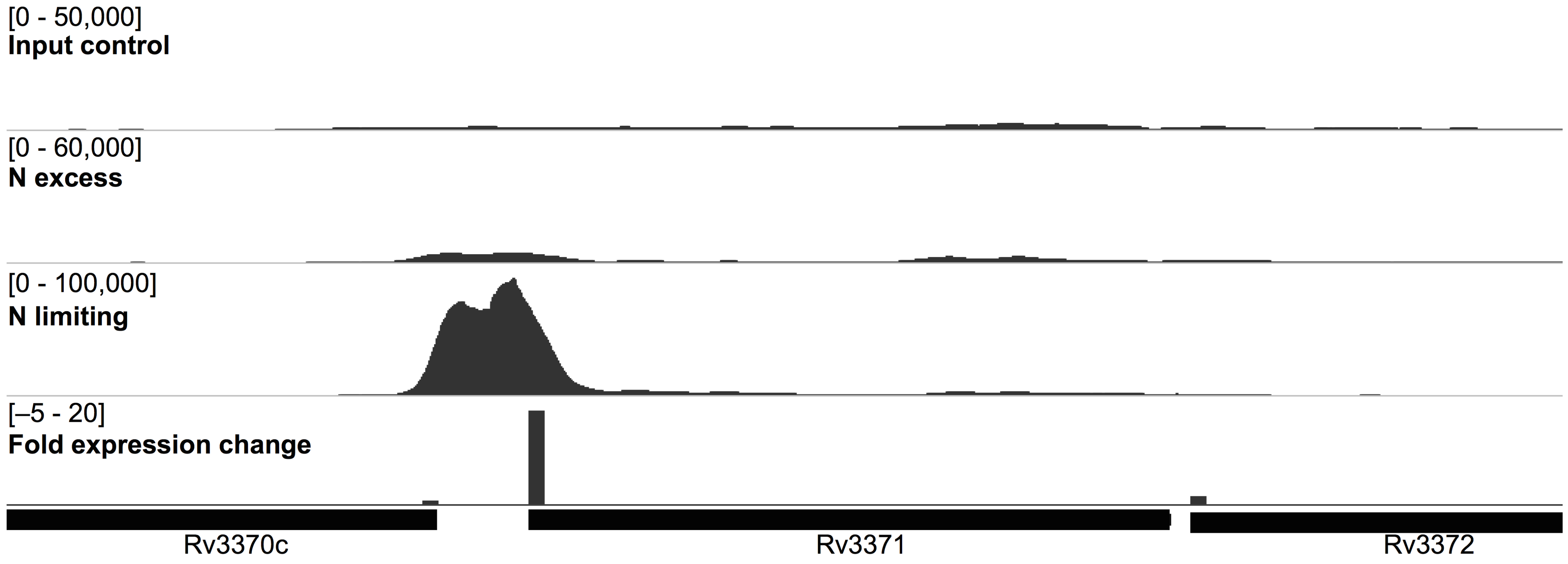
[0 - 50,000]
Input control
[0 - 60,000]
N excess
[0 - 100,000]
N limiting
[–5 - 20]
Fold expression change
Rv3370c
Rv3371
Rv3372

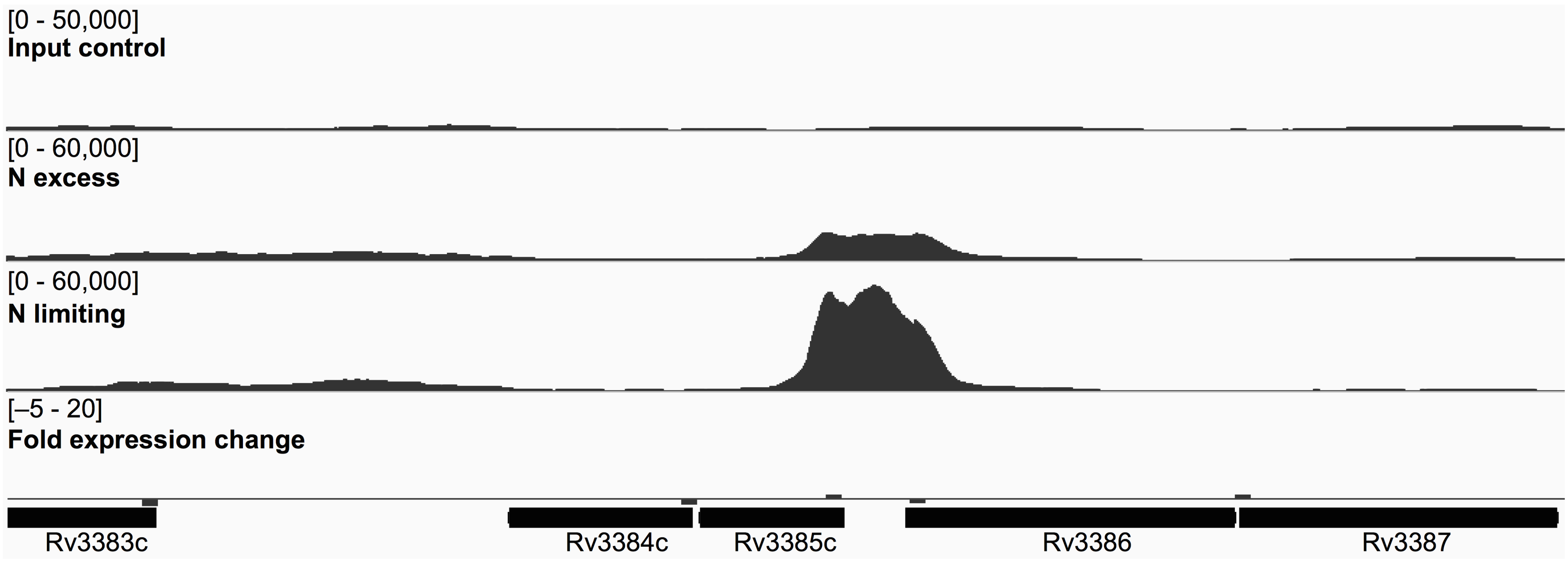
[0 - 50,000]
Input control
[0 - 60,000]
N excess
[0 - 60,000]
N limiting
[–5 - 20]
Fold expression change
Rv3383c
Rv3384c
Rv3385c
Rv3386
Rv3387

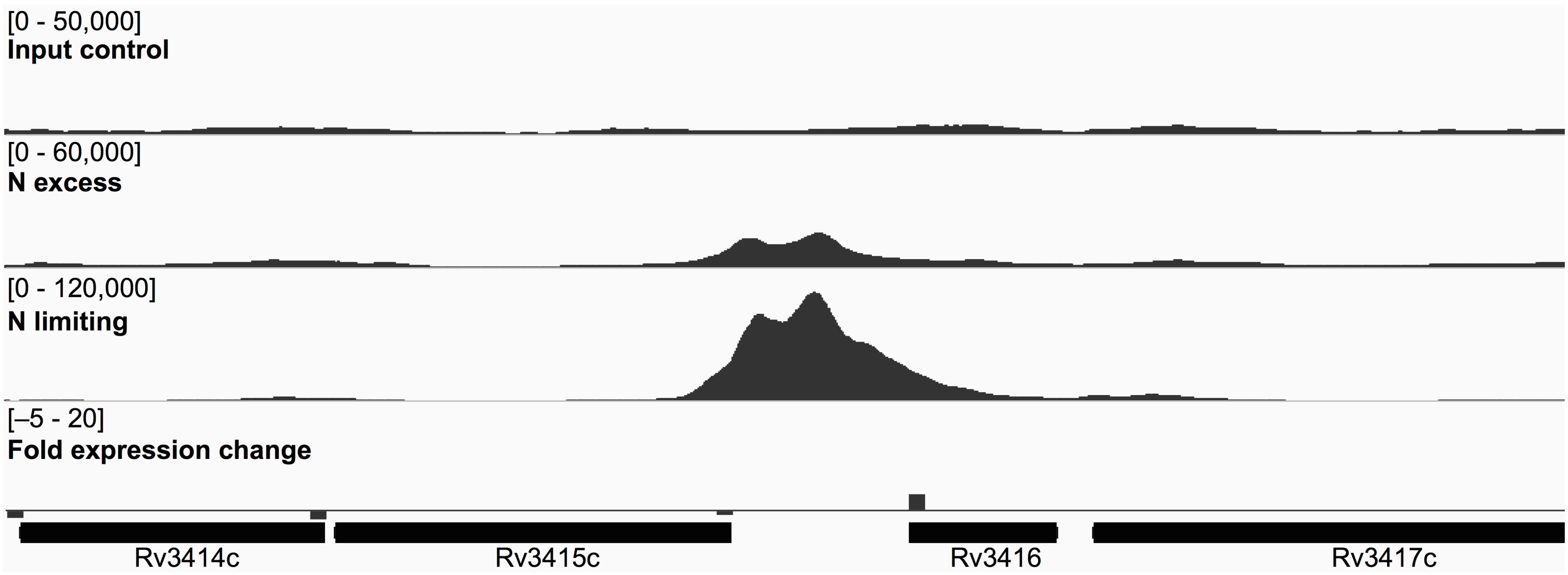
[0 - 50,000]
Input control
[0 - 60,000]
N excess
[0 - 120,000]
N limiting
[–5 - 20]
Fold expression change
Rv3414c
Rv3415c
Rv3416
Rv3417c

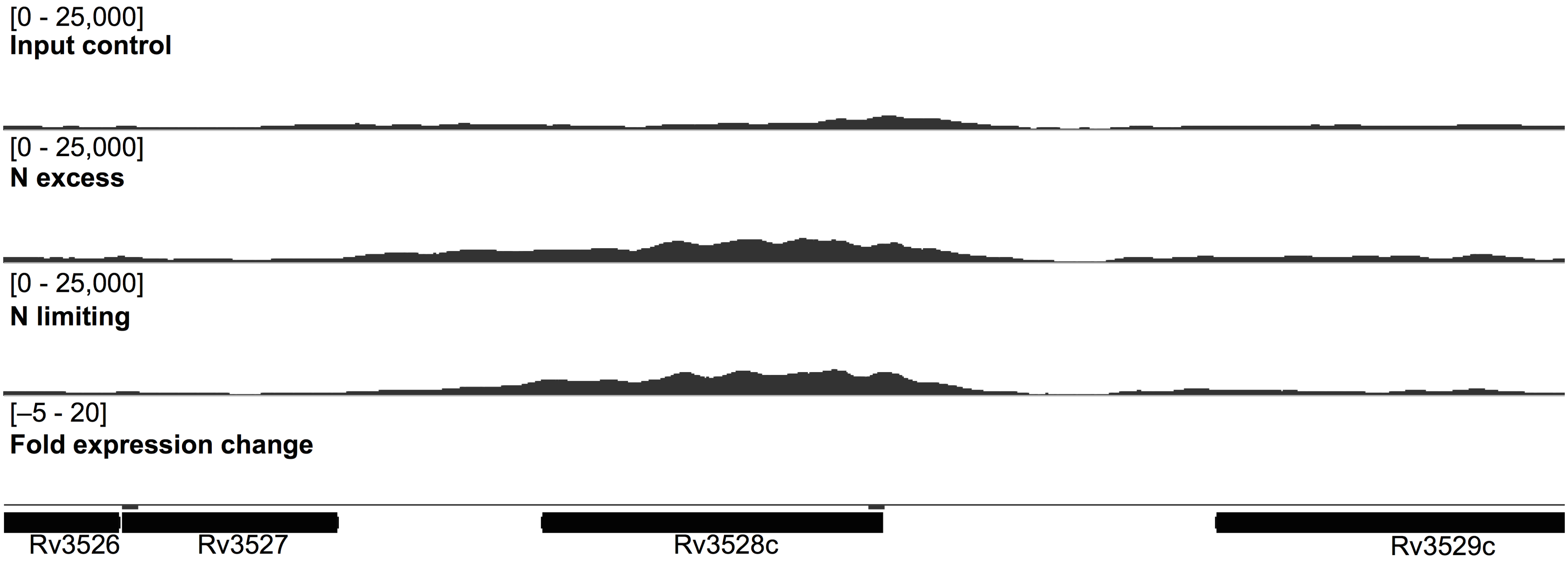
[0 - 25,000]
Input control
[0 - 25,000]
N excess
[0 - 25,000]
N limiting
[–5 - 20]
Fold expression change
Rv3526
Rv3527
Rv3528c
Rv3529c

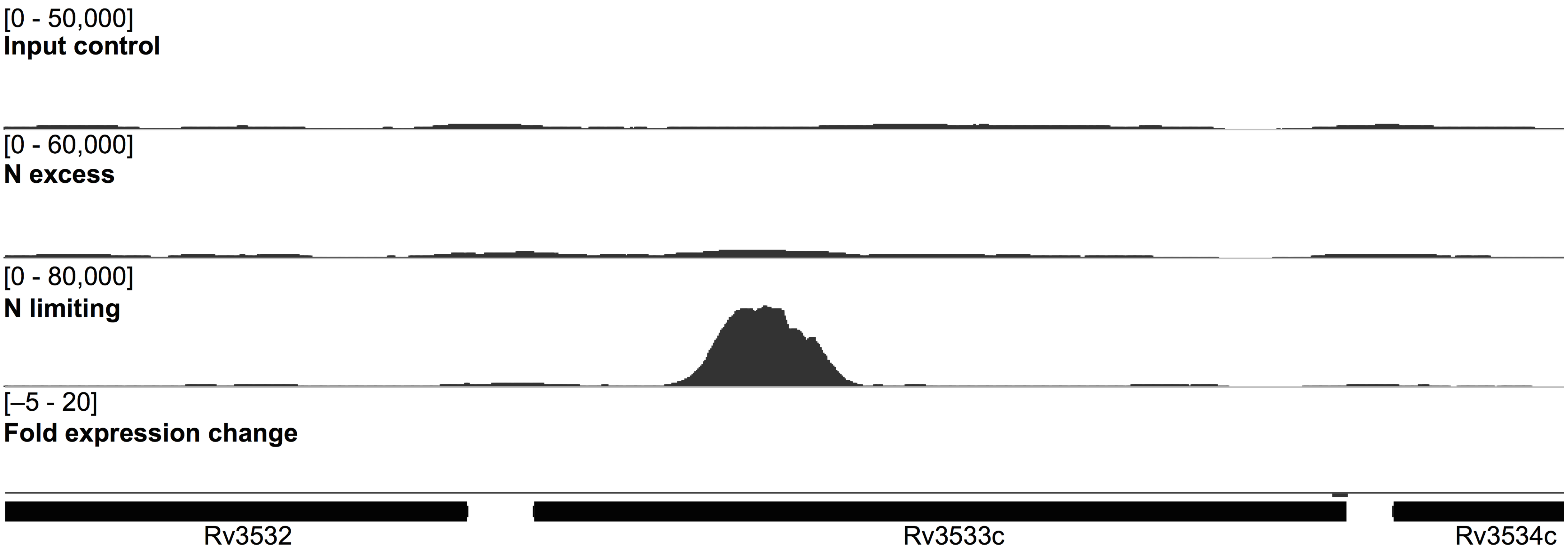

[0 - 50,000]
Input control
[0 - 60,000]
N excess
[0 - 80,000]
N limiting
[–5 - 20]
Fold expression change
Rv3532
Rv3533c
Rv3534c

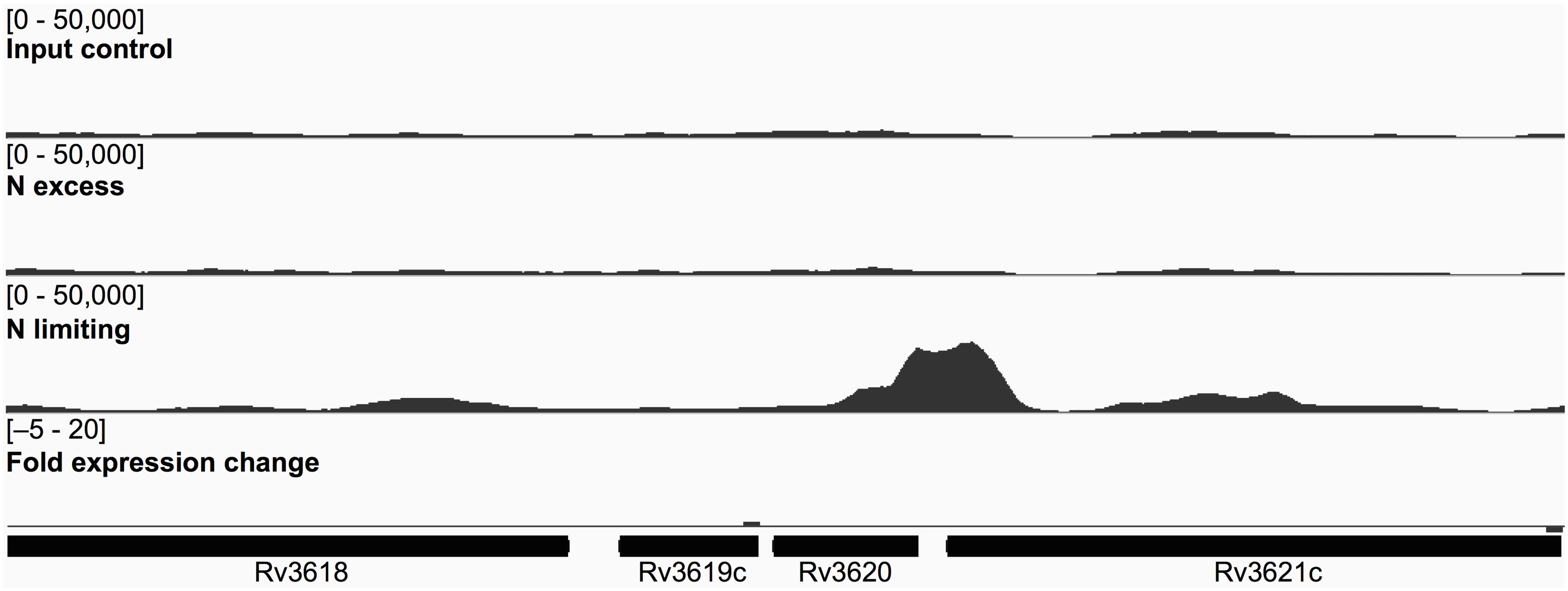
[0 - 50,000]
Input control
[0 - 50,000]
N excess
[0 - 50,000]
N limiting
[–5 - 20]
Fold expression change
Rv3618
Rv3619c
Rv3620
Rv3621c

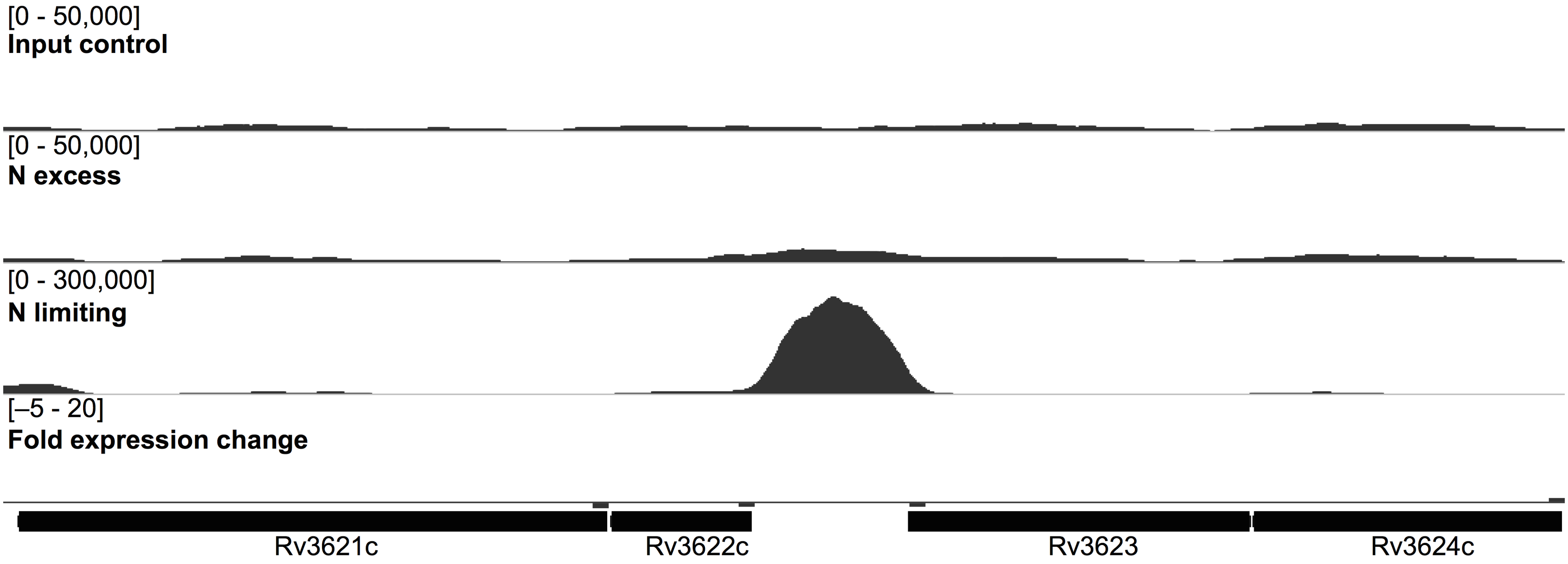

[0 - 50,000]
Input control
[0 - 50,000]
N excess
[0 - 300,000]
N limiting
[–5 - 20]
Fold expression change
Rv3621c
Rv3622c
Rv3623
Rv3624c
