# Supplementary material for: Deciphering the metabolic response of M ycobacterium tuberculosis to nitrogen stress
Source: Mol Microbiol. 2015 Jul 17;97(6):1142–57. doi: 10.1111/mmi.13091 (PMC4950008; doi:10.1111/mmi.13091)
Supplement: Supplementary file 1 — Supporting information [file MMI-97-1142-s001.zip › MMI_13091_supp-0005-Table_S1.docx]

**Table S1. Primers used in this study**

| **Name** | **Application** | **Sequence (5’ – 3’)^a^** |
| --- | --- | --- |
| MTB_GlnR_UpF  MTB_GlnR_UpR  MTB_GlnR_DnF  MTB_GlnR_DnR  MTB_Rv0818_koF  Hyg_Out_1  MTB_Rv0818_koR  Hyg_Out_2  TB_peak18F | Amplification and cloning of GlnR upstream region into pYUB854 for mutant construction  Amplification and cloning of GlnR downstream region into pYUB854 for mutant construction  Confirmation of GlnR replacement with hygromycin cassette  Confirmation of GlnR replacement with hygromycin cassette  Amplification of peak 18 for EMSA | GATCATCTTAAGCCAGCGCACCTGTAGCAACG  GATCTCTAGATACAGCTCCGAGGTCAGCAG  GATCATAAGCTTATGGTATGCAAGAACCACTG  GATCACTAGTCAGCGCGTAGGCGGTATCG  AAGCCAACAGTGGTTTCAG  GCATGCAAGCTCAGGATGTC  TAGATATCTTGATCATCGAG  TTCGAGGTGTTCGAGGAGAC  ACCATCCCGTCAGCCGGCCACAC |
| TB_peak18R |  | GTACGTCCACAATCGAAGGA |
| TB_peak13R | Amplification of peak 13 for EMSA | GCTAAATCCCACCAGCATG |
| TB_peak13R |  | CACAGACTCCATCTGTTG |
| TB_1360R | Amplification of Rv1360 upstream region for EMSA | ACTCCCTGCGGCAAGGTG |
| TB_1360R |  | GACATACGTGGATGTGCTG |
| TB_peak17F | Amplification of peak 17 for EMSA | GATCTTGTCGTAGATGCTG |
| TB_peak17R |  | CATGAGCTGATGAATGGAGT |
| TB_peak20F | Amplification of peak 20 for EMSA | GATATTGCCCGTCAGTC |
| TB_peak20R |  | TTCGGCATGCCACCGGTTAC |
| TB_nirBF | Amplification of *nirB* for rate limiting PCR | CTTCGTTGTGAGTTAGC |
| TB_nirBR |  | ATCGCCGAATGTGACGCAC |
| TB_peak2F | Amplification of peak 2 for rate limiting PCR | CGAAGCAATGCGCACAG |
| TB_peak2R |  | TGGCCTACGTCTAGCG |
| TB_peak10F | Amplification of peak 10 for rate limiting PCR | GACAACACCAAGTTCGC |
| TB_peak10R |  | ACGGCAGGTCGGTGTAGC |
| TB_peak11F | Amplification of peak 11 for rate limiting PCR | GCTTGCCACCGCCGAC |
| TB_peak11R |  | ACCGACAGCGAGTAGGC |
| TB_peak23F | Amplification of peak 23 for rate limiting PCR | TCGAAGCGACCAGGCAG |
| TB_peak23R  MTB_GlnR_Comp_306_F  MTB_GlnR_Comp_316_F  MTB_GlnR_Comp_R | Cloning of MTB *glnR* into pMV306/pMV361 to complement null mutant | ACCTCCGTGTTGCCTGC  GATGATGGTACCCCCACGATGAGAACG  GATGATAAGCTTTTGTTGGAGTTATTAC  GATGATATCGATTGACCAGTGCACGCACGC |
|  |  |  |

^a^ Restriction sites used for cloning are underlined.
