# Supplementary material for: Deciphering the metabolic response of M ycobacterium tuberculosis to nitrogen stress
Source: Mol Microbiol. 2015 Jul 17;97(6):1142–57. doi: 10.1111/mmi.13091 (PMC4950008; doi:10.1111/mmi.13091)
Supplement: Supplementary file 1 — Supporting information [file MMI-97-1142-s001.zip › MMI_13091_supp-0006-Table_S2.docx]

**Table S2.** Custom Taqman *M. tuberculosis* gene expression primer and probe sequences used in this study.

| **Gene** | **Forward primer (5’-3’)** | **Reverse primer (5’-3’)** | **FAM Reporter probe (5’-3’)** |
| --- | --- | --- | --- |
| *nirB* (Rv0252) | CCGGATTCGGCCCAGAT | CTTCAGCTCGCCCTTGGT | CTGCTCGTGCAACAAC |
| *amt* (Rv2920) | CCCCGGACCTGCATCAG | CAGCGCGCGTTCCAT | CTCCGCGACTAGCTCC |
| *glnR (Rv0818)* | TGGCTGACCAGGAGAGTCT | AGGTGCCTTCGTCGATCAC | TCGCCCAGGCTCACC |
| *sigA* (Rv2703) | GCCACGCAGCTGATGAC | GCGGCAGGCAGCTTT | TCGCCGCGCTCGCTAA |
