# Supplementary material for: Deciphering the metabolic response of M ycobacterium tuberculosis to nitrogen stress
Source: Mol Microbiol. 2015 Jul 17;97(6):1142–57. doi: 10.1111/mmi.13091 (PMC4950008; doi:10.1111/mmi.13091)
Supplement: Supplementary file 1 — Supporting information [file MMI-97-1142-s001.zip › MMI_13091_supp-0007-Williams_Supplementary_figures.pdf]

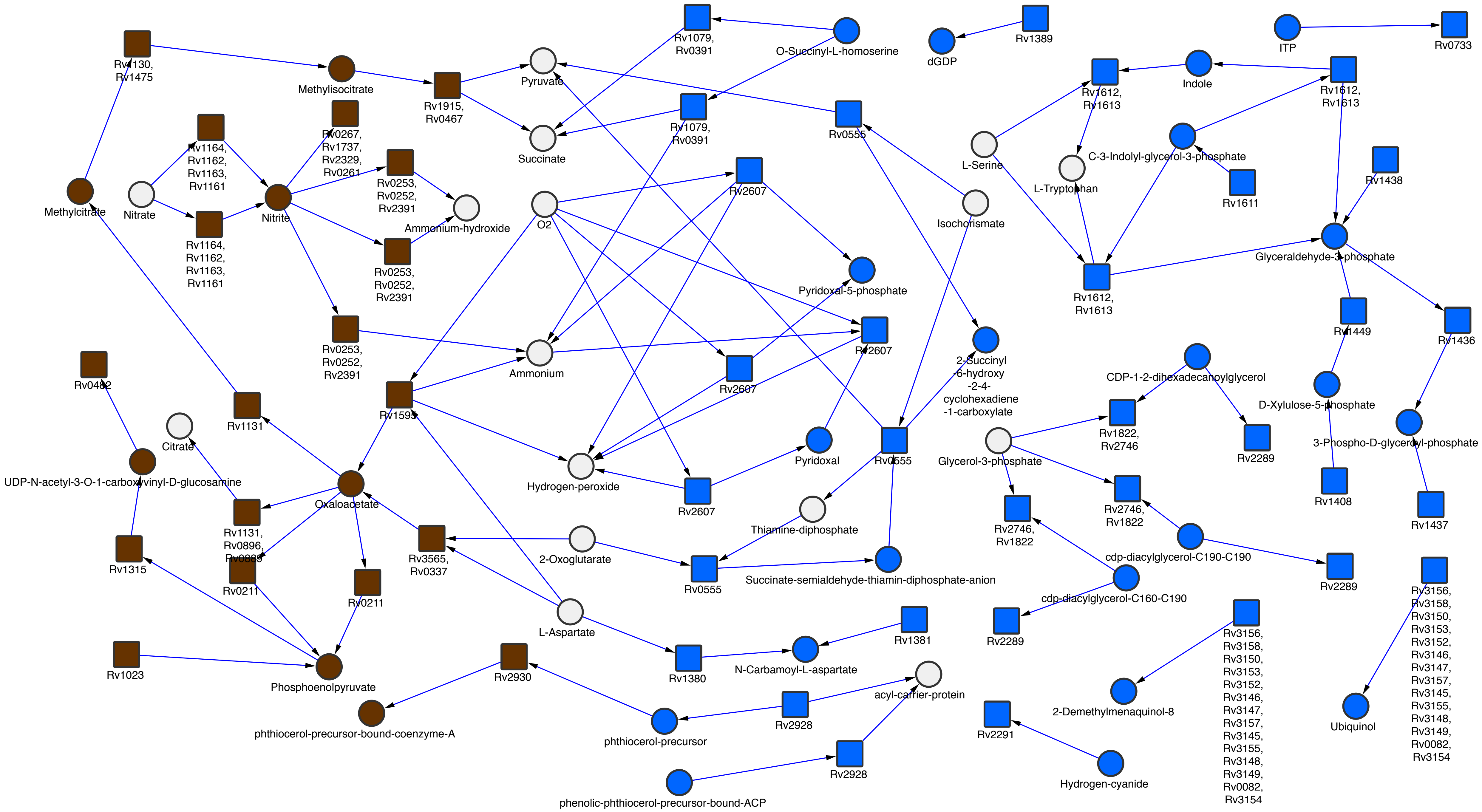
Rv1130, Rv1475
Methylisocitrate
Rv1915, Rv0467
Pyruvate
Succinate
Rv1079, Rv0391
O-Succinyl-L-homoserine
Rv1079, Rv0391
dGDP
Rv1389
ITP
Rv0733
Indole
Rv1612, Rv1613
Rv1612, Rv1613
L-Serine
L-Tryptophan
C-3-Indolyl-glycerol-3-phosphate
Rv1611
Rv1438
Rv1612, Rv1613
Glyceraldehyde-3-phosphate
Rv1449
Rv1436
D-Xylulose-5-phosphate
3-Phospho-D-glyceroyl-phosphate
Rv1408
Rv1437
Rv1164, Rv1162, Rv1163, Rv1161
Rv0267, Rv1737, Rv2329, Rv0261
Rv0253, Rv0252, Rv2391
Methylcitrate
Nitrate
Nitrite
Rv1164, Rv1162, Rv1163, Rv1161
Ammonium-hydroxide
Rv0253, Rv0252, Rv2391
O2
Rv2607
Rv0555
Isochorismate
Pyridoxal-5-phosphate
Rv2607
Rv2607
2-Succinyl -6-hydroxy -2-4- cyclohexadiene -1-carboxylate
CDP-1-2-dihexadecanoylglycerol
Rv0253, Rv0252, Rv2391
Ammonium
Rv0482
Rv1131
Rv1595
Citrate
UDP-N-acetyl-3-O-1-carboxyvinyl-D-glucosamine
Oxaloacetate
Hydrogen-peroxide
Pyridoxal
Rv0555
Rv1822, Rv2746
Rv2289
Glycerol-3-phosphate
Rv2746, Rv1822
Rv2746, Rv1822
cdp-diacylglycerol-C190-C190
Rv2289
Rv1131, Rv0896, Rv0889
Rv2607
Thiamine-diphosphate
Rv1315
Rv0211
Rv0211
Rv3565, Rv0337
2-Oxoglutarate
Rv0555
Succinate-semialdehyde-thiamin-diphosphate-anion
cdp-diacylglycerol-C160-C190
Rv2289
L-Aspartate
Rv1381
N-Carbamoyl-L-aspartate
Rv1380
Rv1023
Phosphoenolpyruvate
Rv2930
phthiocerol-precursor-bound-coenzyme-A
phthiocerol-precursor
Rv2928
acyl-carrier-protein
Rv2928
phenolic-phthiocerol-precursor-bound-ACP
Rv2291
Hydrogen-cyanide
2-Demethylmenaquinol-8
Rv3156, Rv3158, Rv3150, Rv3153, Rv3152, Rv3146, Rv3147, Rv3157, Rv3145, Rv3155, Rv3148, Rv3149, Rv0082, Rv3154
Ubiquinol
Rv3156, Rv3158, Rv3150, Rv3153, Rv3152, Rv3146, Rv3147, Rv3157, Rv3145, Rv3155, Rv3148, Rv3149, Rv0082, Rv3154

# Figure S2: *glnR* KO Characterisation

A

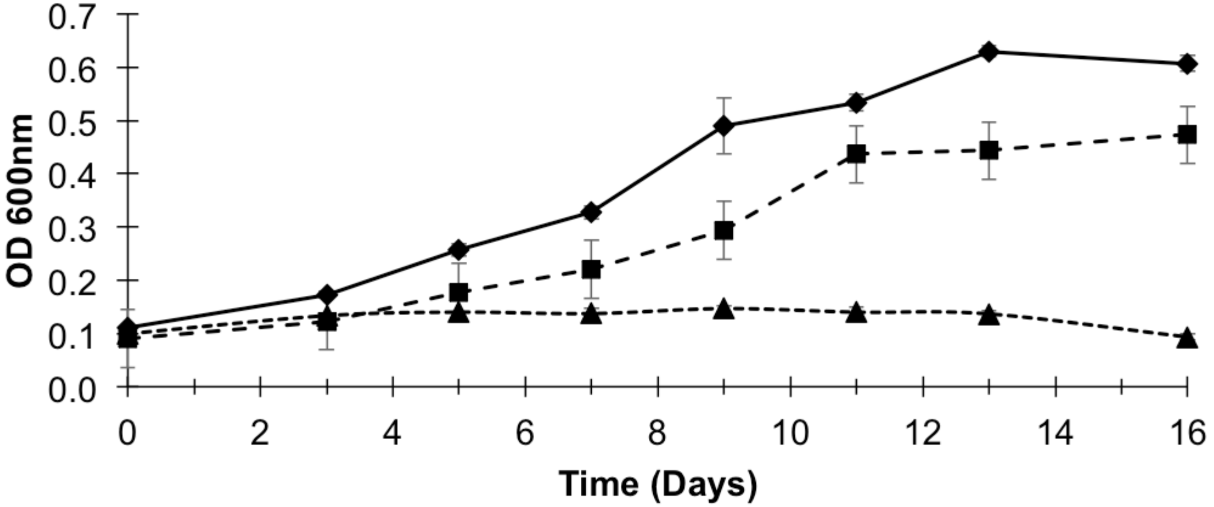

# Figure S2: *glnR* KO Characterisation

**B**

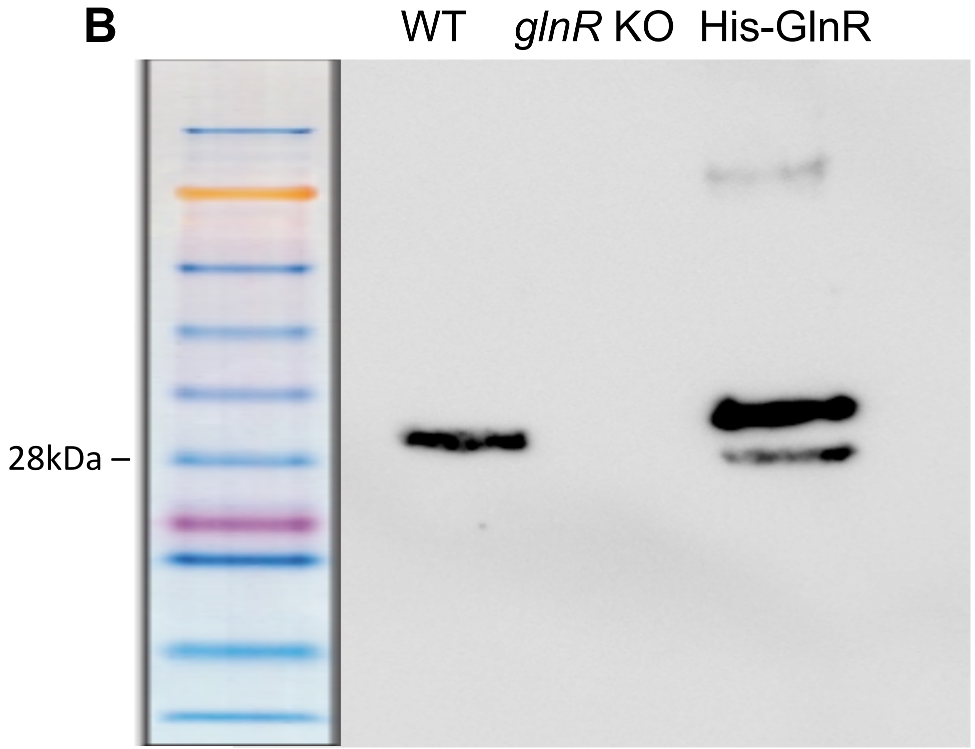


Western blot with GlnR Ab, confirming null phenotype

# Figure S2: *glnR* KO Characterisation

**C**

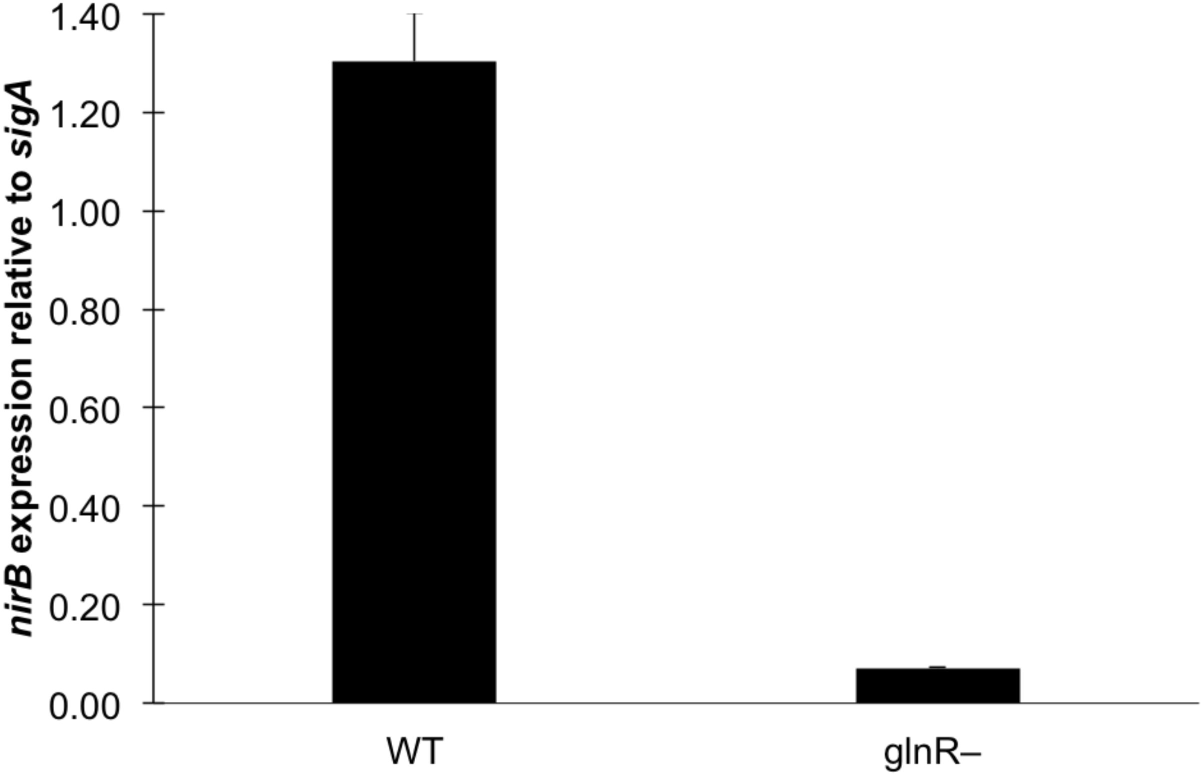

# Figure S2: *glnR* KO Characterisation

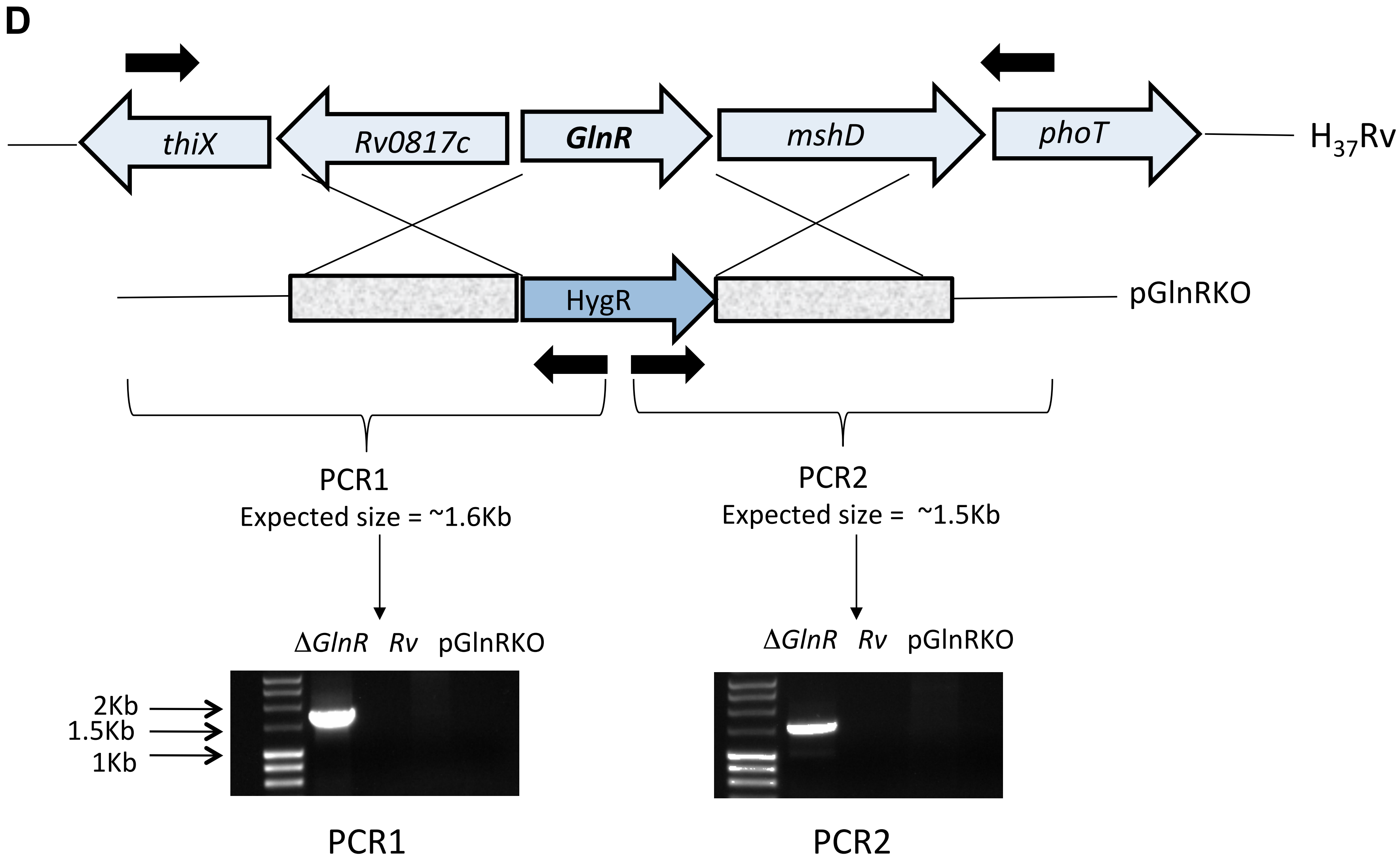

# Figure S3 – qPCR of *nirB* and negative control

Confirmed enrichment in Low N before ChIP library was made

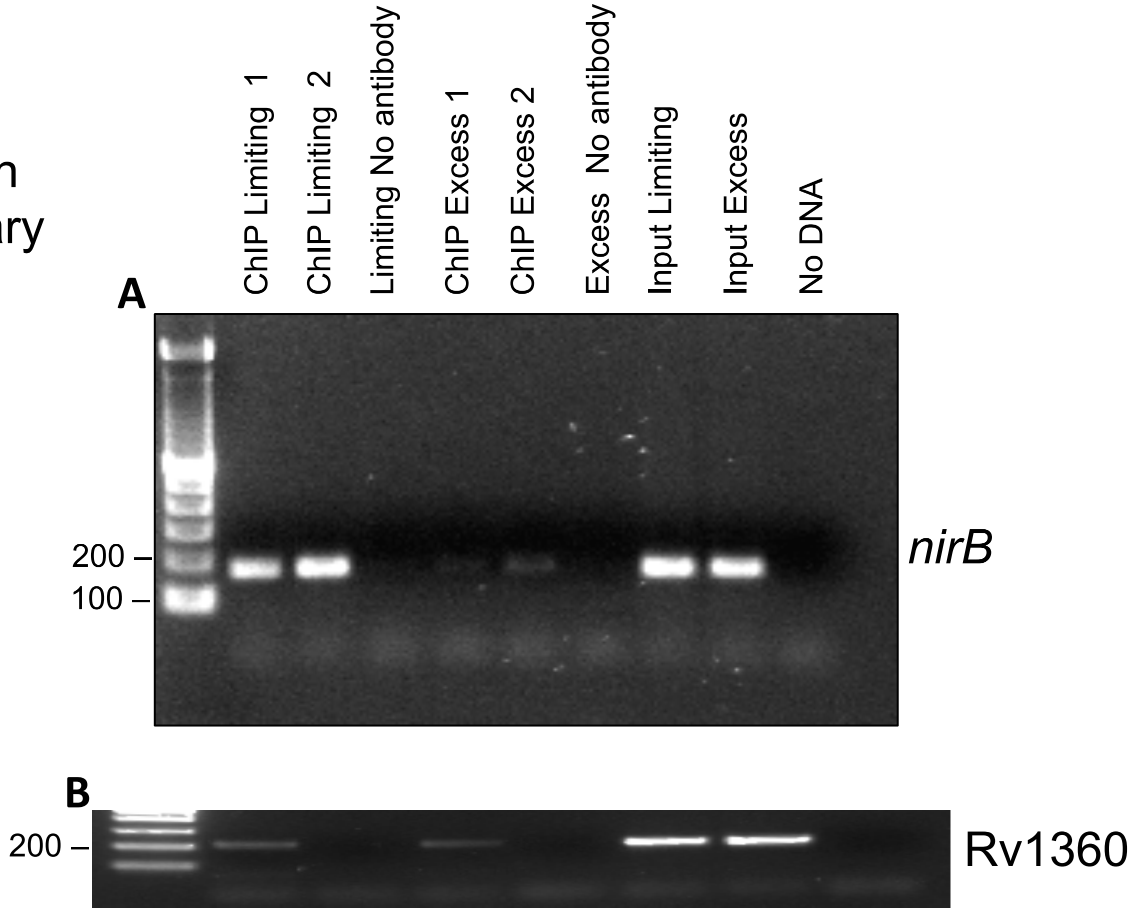

# Figure S4 - EMSA Negative control

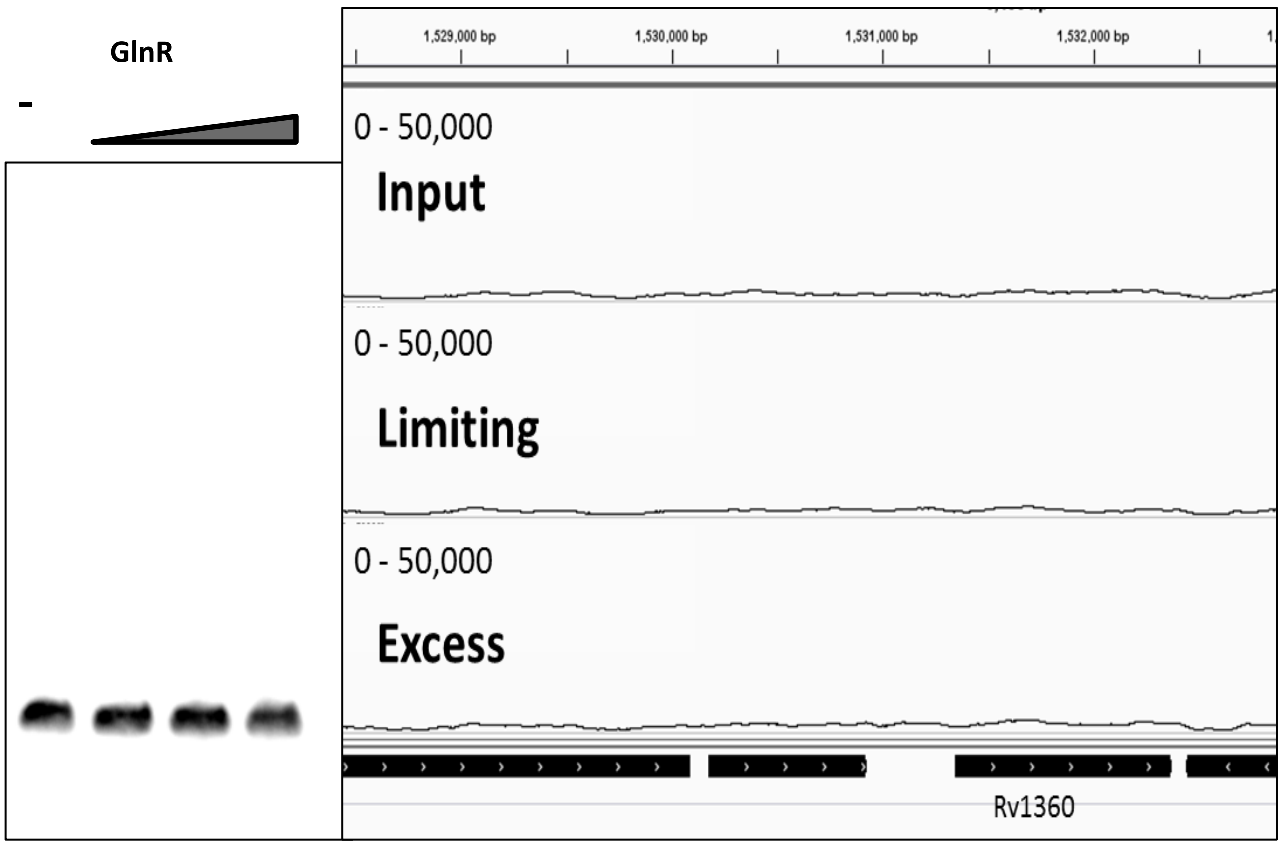

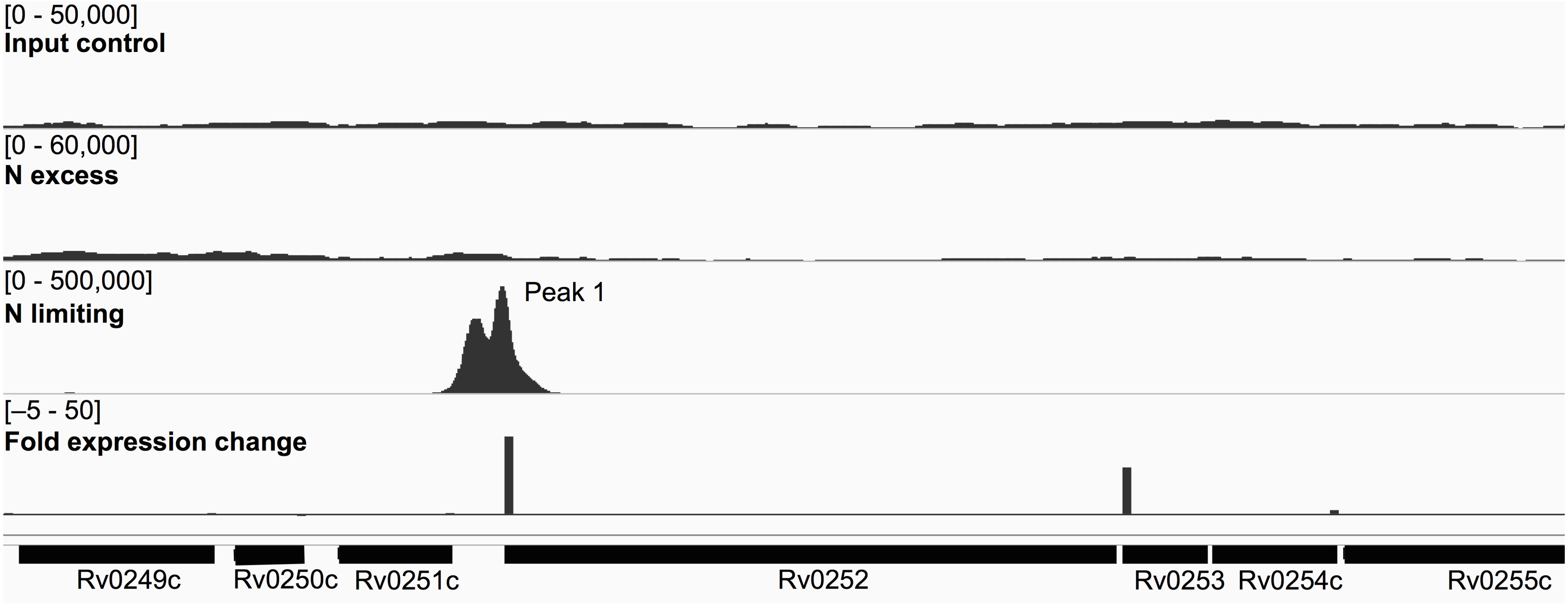
[0 - 50,000]
Input control
[0 - 60,000]
N excess
[0 - 500,000]
N limiting
Peak 1
[–5 - 50]
Fold expression change
Rv0249c
Rv0250c
Rv0251c
Rv0252
Rv0253
Rv0254c
Rv0255c

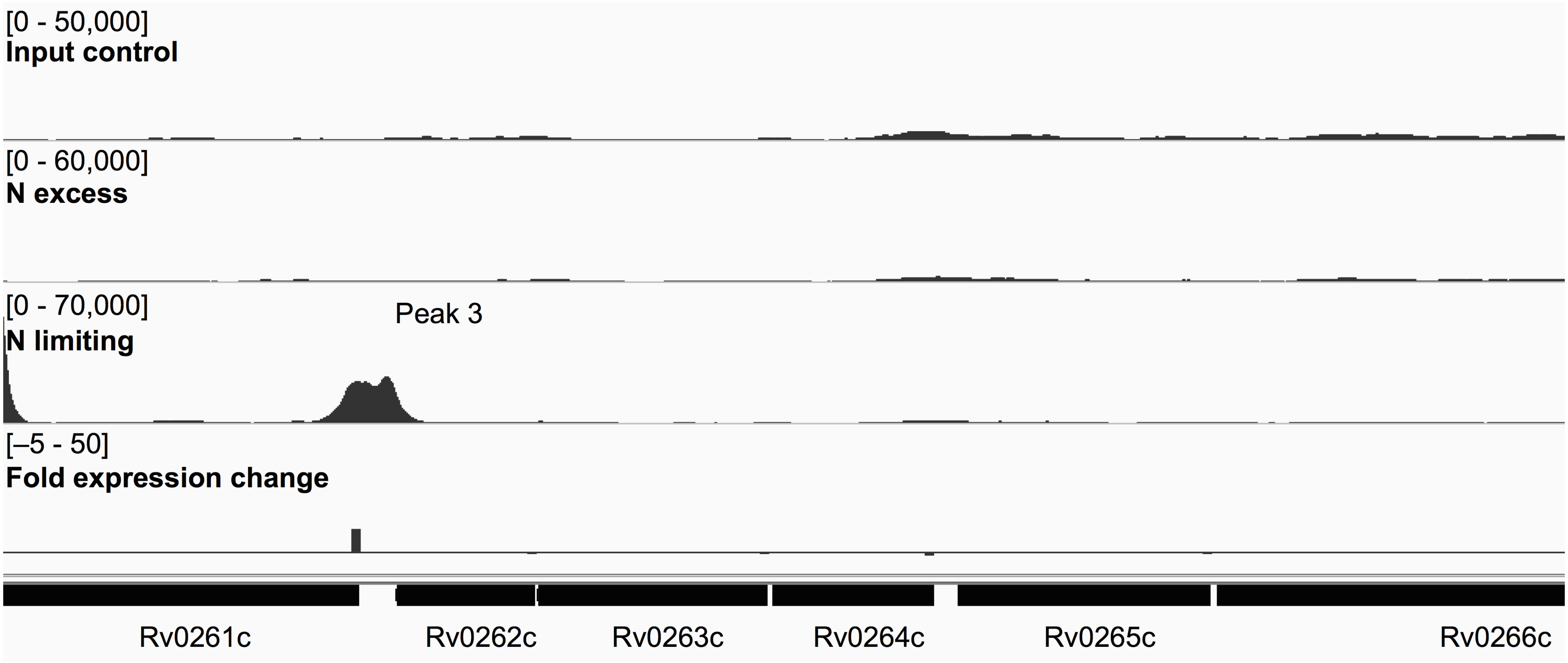
[0 - 50,000]
Input control
[0 - 60,000]
N excess
[0 - 70,000]
N limiting
Peak 3
[–5 - 50]
Fold expression change
Rv0261c
Rv0262c
Rv0263c
Rv0264c
Rv0265c
Rv0266c

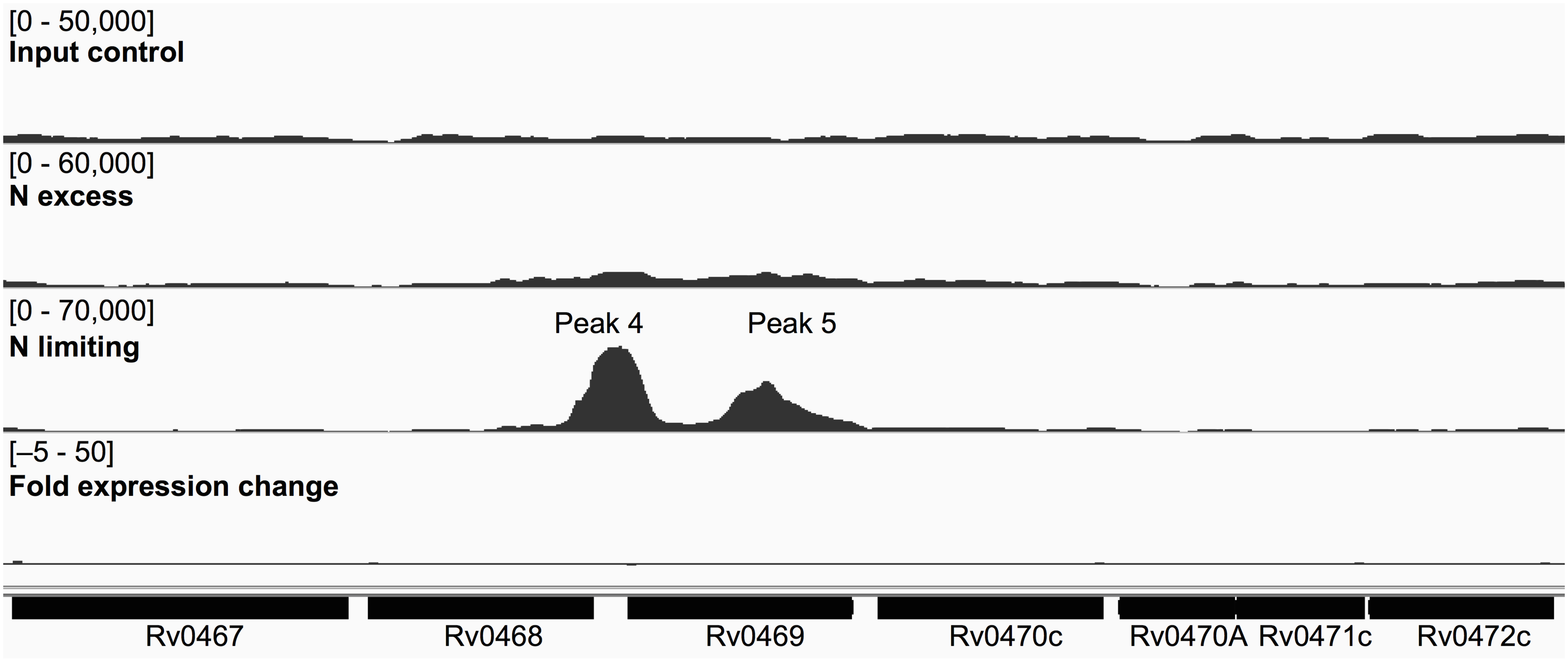
[0 - 50,000]
Input control
[0 - 60,000]
N excess
[0 - 70,000]
N limiting
Peak 4
Peak 5
[–5 - 50]
Fold expression change
Rv0467
Rv0468
Rv0469
Rv0470c
Rv0470A
Rv0471c
Rv0472c

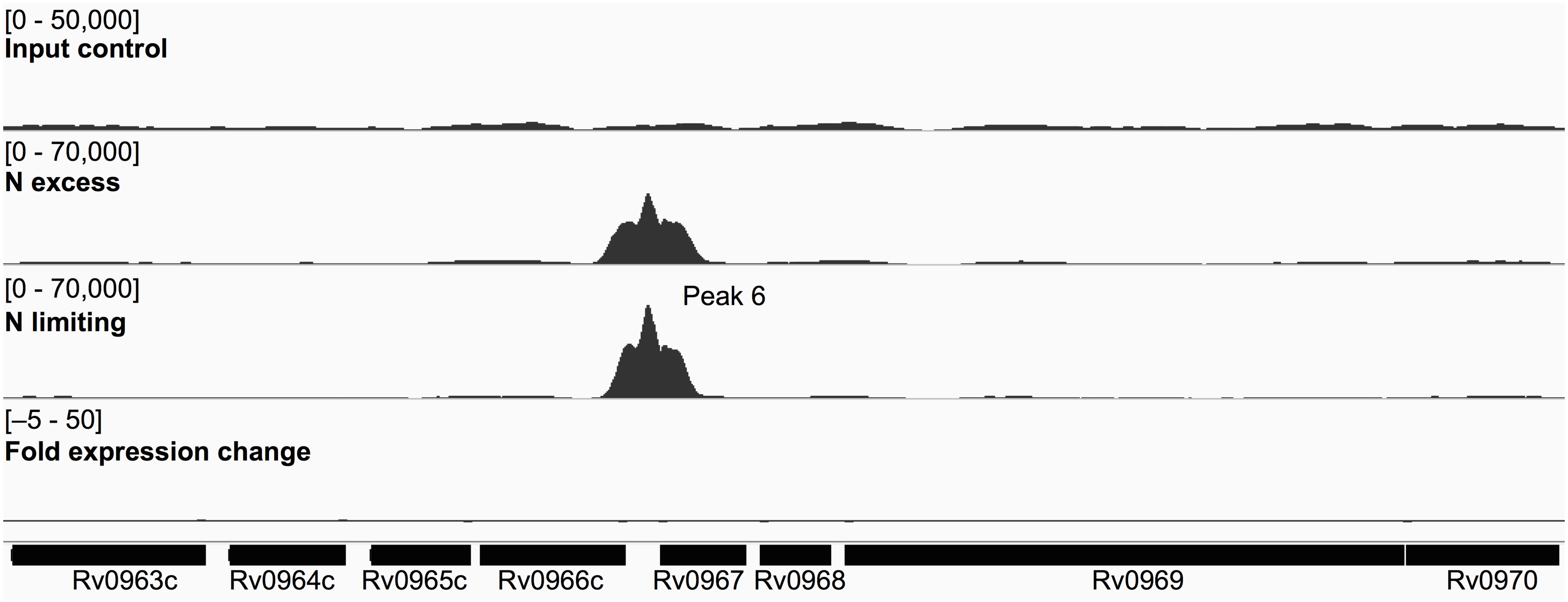
[0 - 50,000]
Input control
[0 - 70,000]
N excess
[0 - 70,000]
N limiting
Peak 6
[–5 - 50]
Fold expression change
Rv0963c
Rv0964c
Rv0965c
Rv0966c
Rv0967
Rv0968
Rv0969
Rv0970

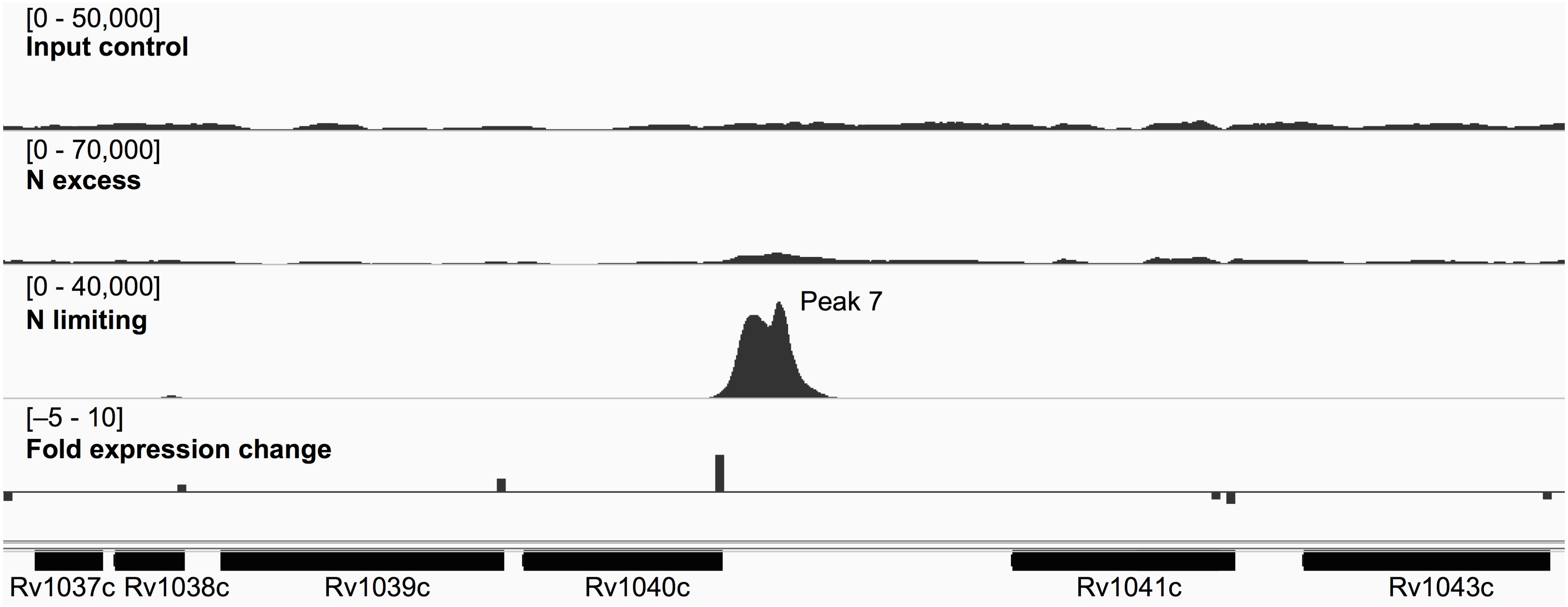
[0 - 50,000]
Input control
[0 - 70,000]
N excess
[0 - 40,000]
N limiting
Peak 7
[–5 - 10]
Fold expression change
Rv1037c
Rv1038c
Rv1039c
Rv1040c
Rv1041c
Rv1043c

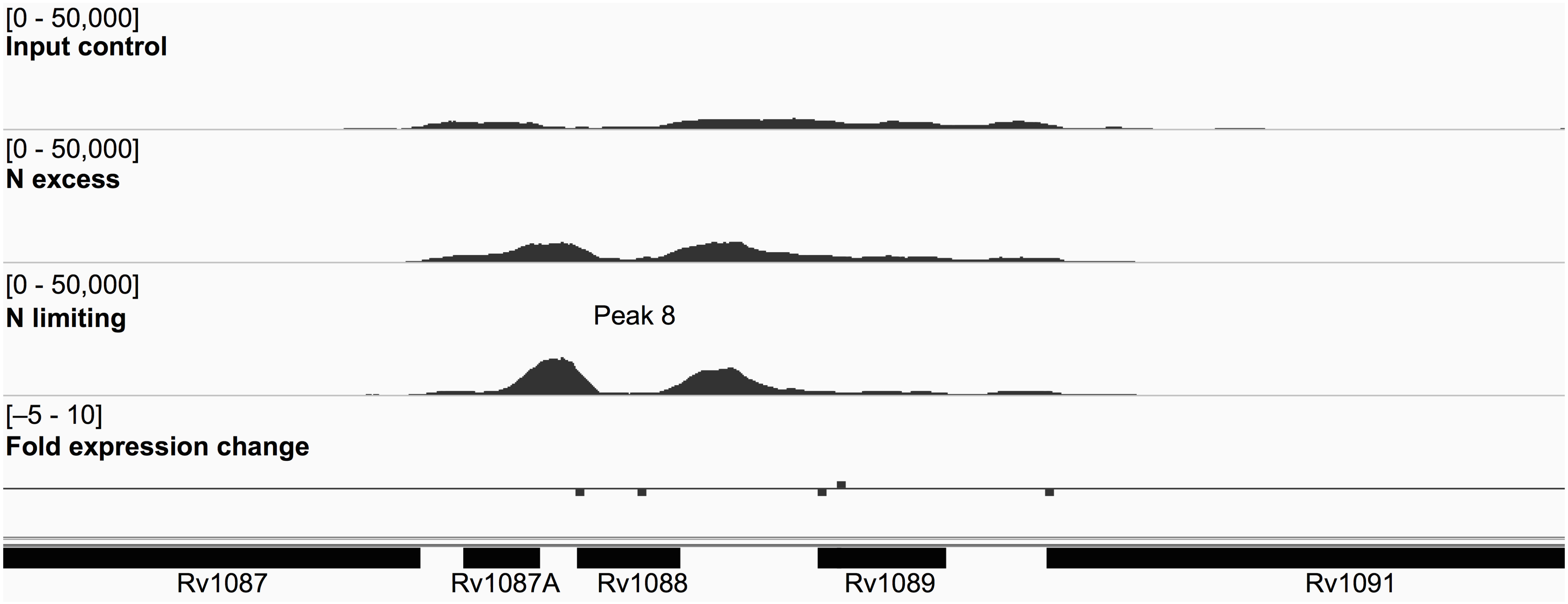
[0 - 50,000]
Input control
[0 - 50,000]
N excess
[0 - 50,000]
N limiting
Peak 8
[–5 - 10]
Fold expression change
Rv1087
Rv1087A
Rv1088
Rv1089
Rv1091

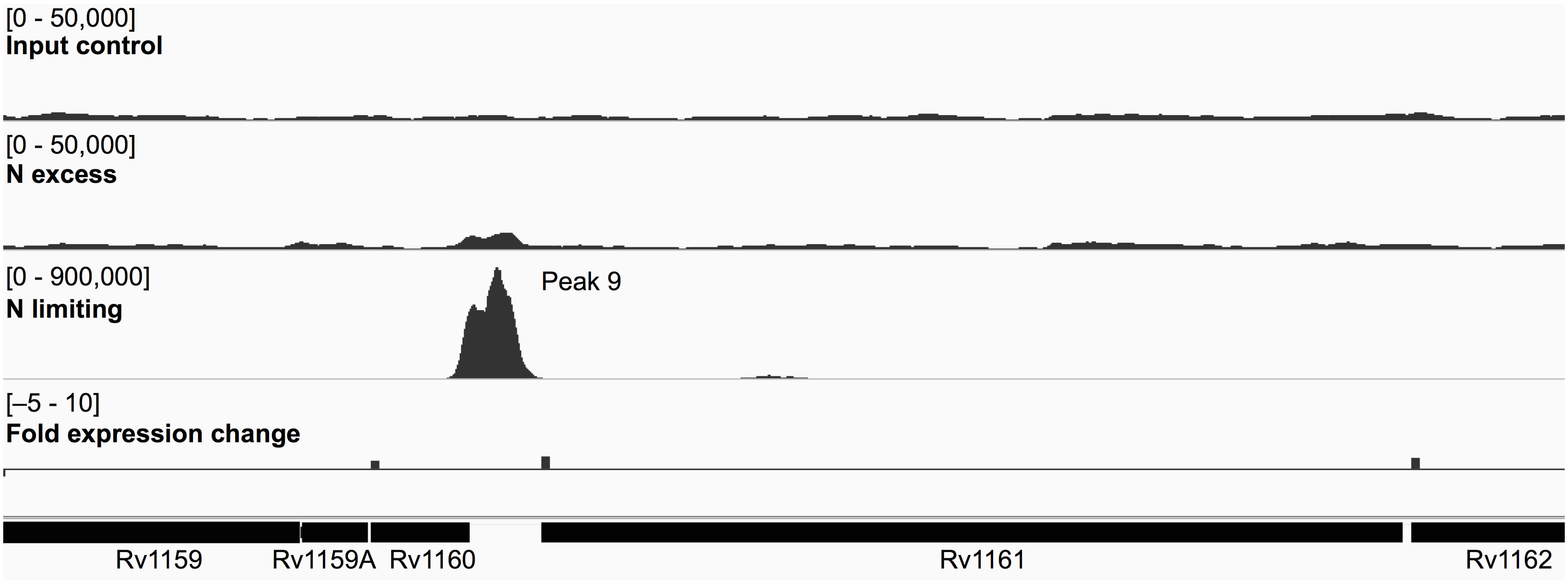

[0 - 50,000]
Input control
[0 - 50,000]
N excess
[0 - 900,000]
N limiting
Peak 9
[–5 - 10]
Fold expression change
Rv1159
Rv1159A
Rv1160
Rv1161
Rv1162

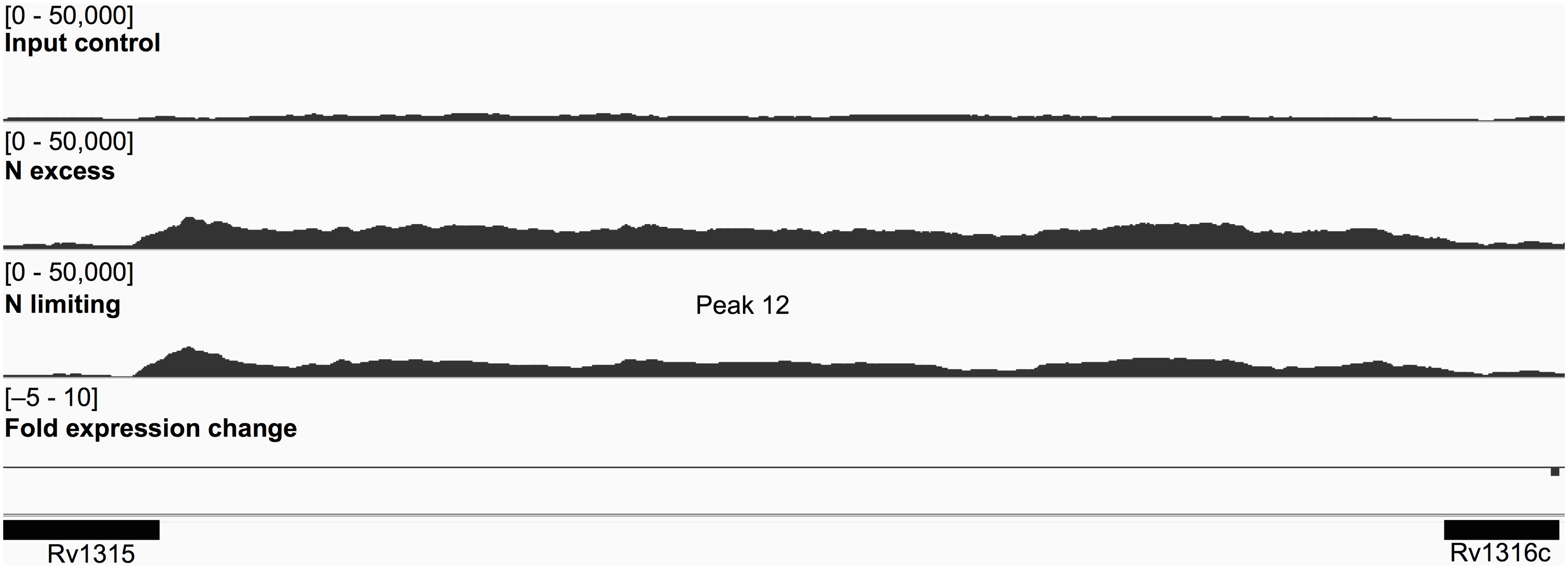
[0 - 50,000]
Input control
[0 - 50,000]
N excess
[0 - 50,000]
N limiting
Peak 12
[–5 - 10]
Fold expression change
Rv1315
Rv1316c

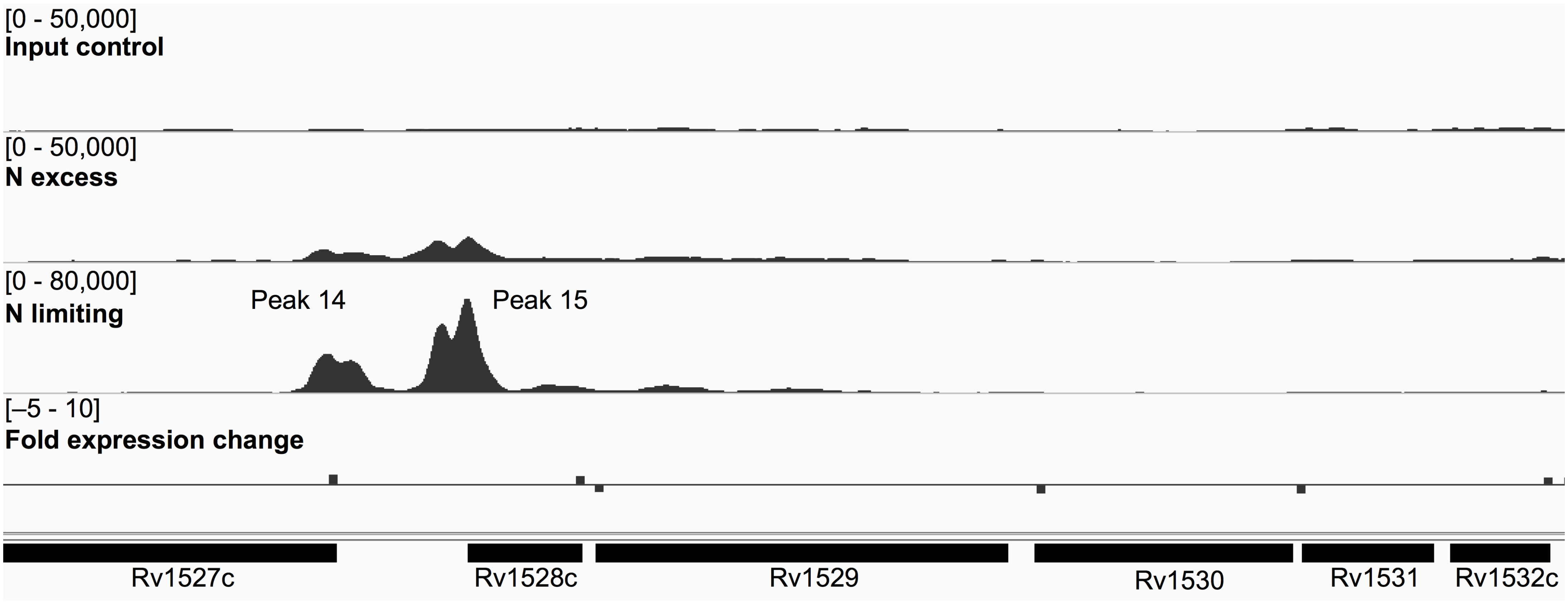
[0 - 50,000]
Input control
[0 - 50,000]
N excess
[0 - 80,000]
N limiting
Peak 14
Peak 15
[–5 - 10]
Fold expression change
Rv1527c
Rv1528c
Rv1529
Rv1530
Rv1531
Rv1532c

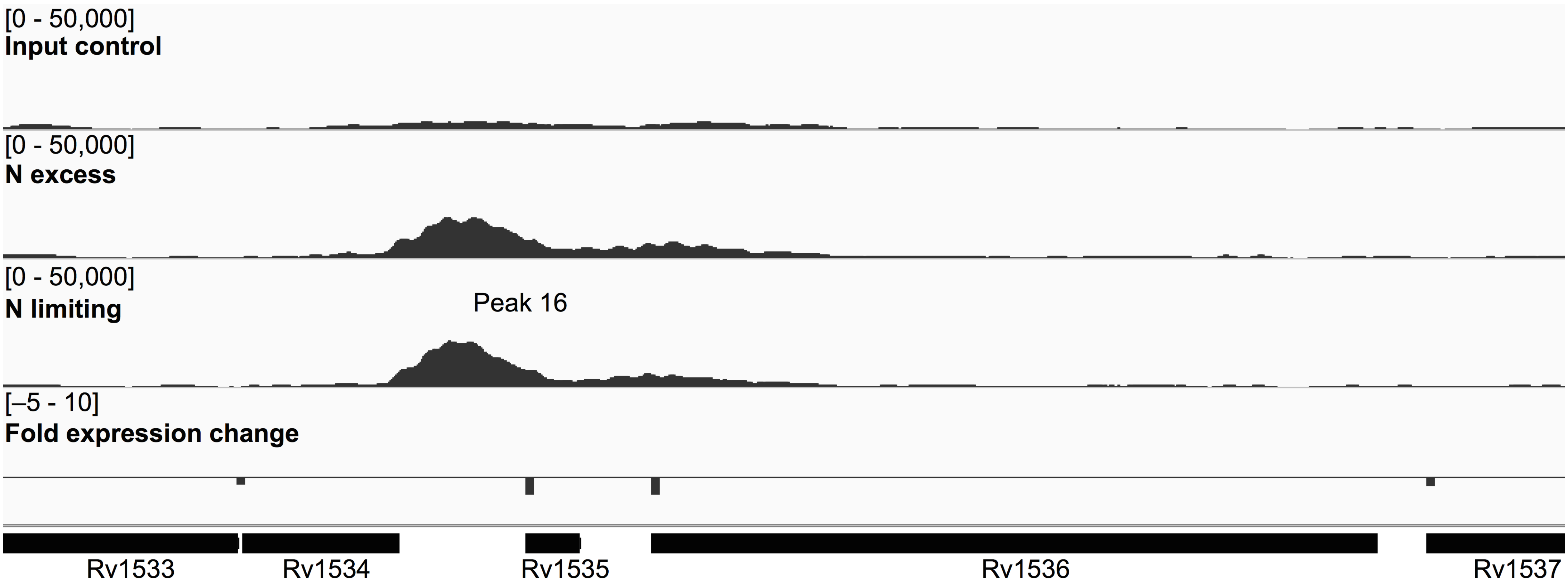

[0 - 50,000]
Input control
[0 - 50,000]
N excess
[0 - 50,000]
N limiting
Peak 16
[–5 - 10]
Fold expression change
Rv1533
Rv1534
Rv1535
Rv1536
Rv1537

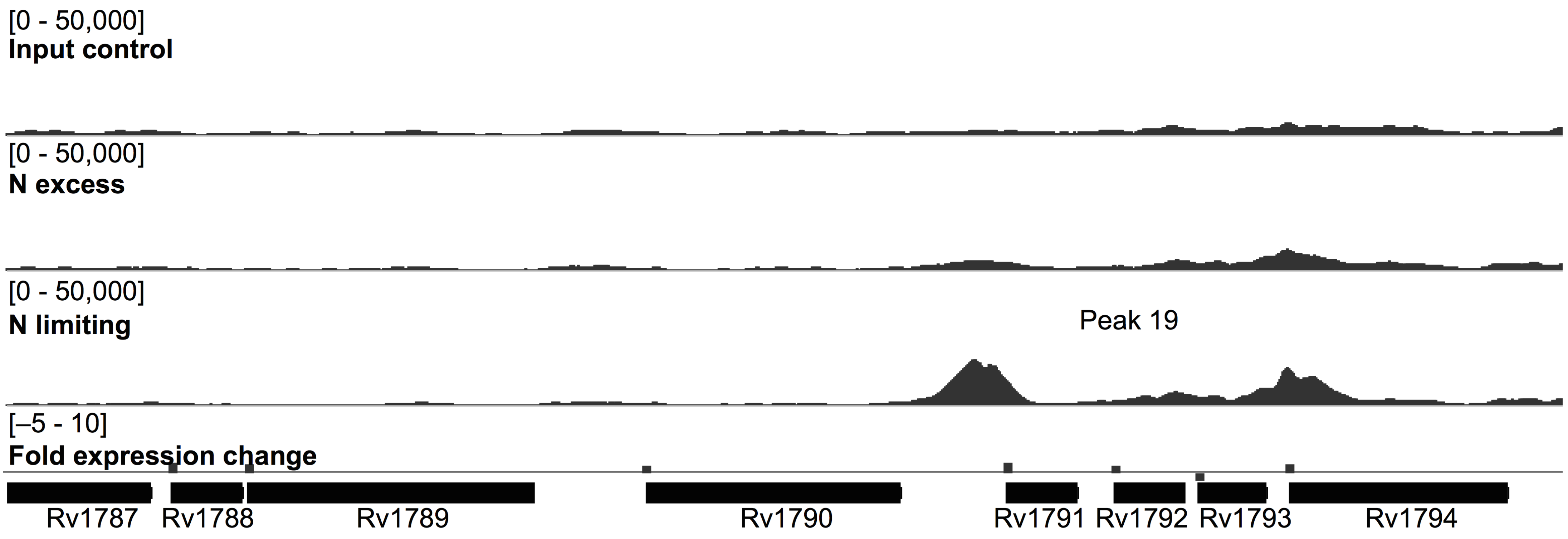

[0 - 50,000]
Input control
[0 - 50,000]
N excess
[0 - 50,000]
N limiting
Peak 19
[–5 - 10]
Fold expression change
Rv1787
Rv1788
Rv1789
Rv1790
Rv1791
Rv1792
Rv1793
Rv1794

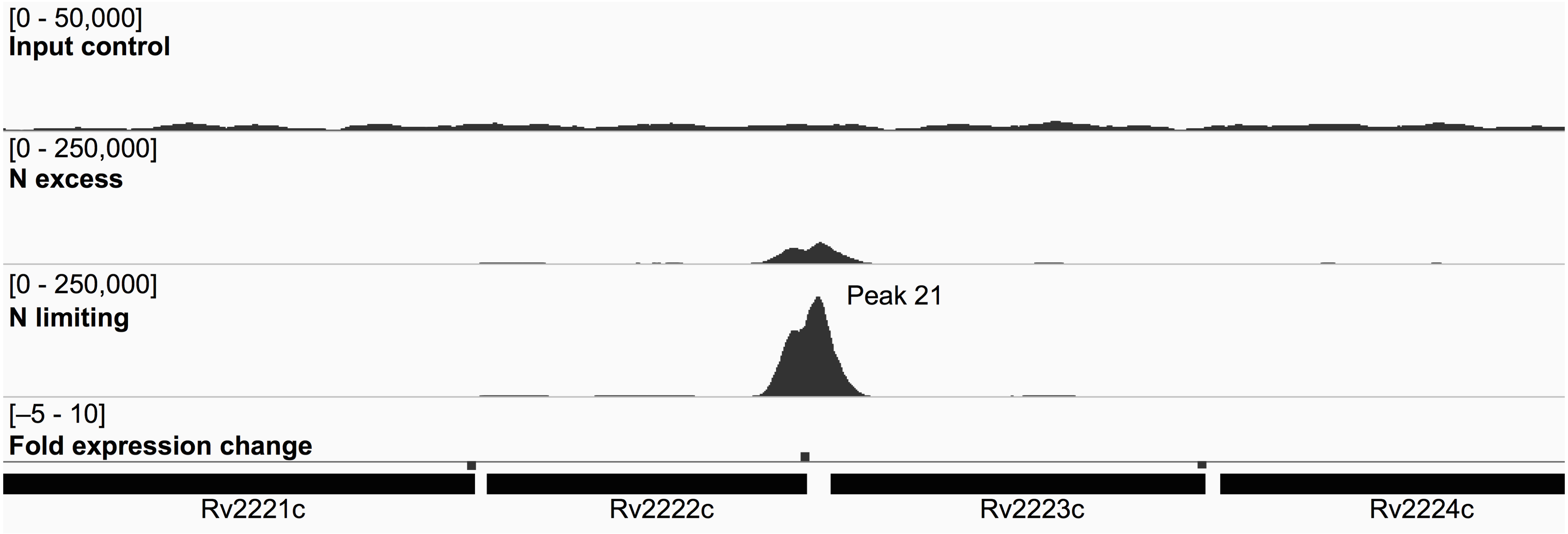
[0 - 50,000]
Input control
[0 - 250,000]
N excess
[0 - 250,000]
N limiting
Peak 21
[–5 - 10]
Fold expression change
Rv2221c
Rv2222c
Rv2223c
Rv2224c

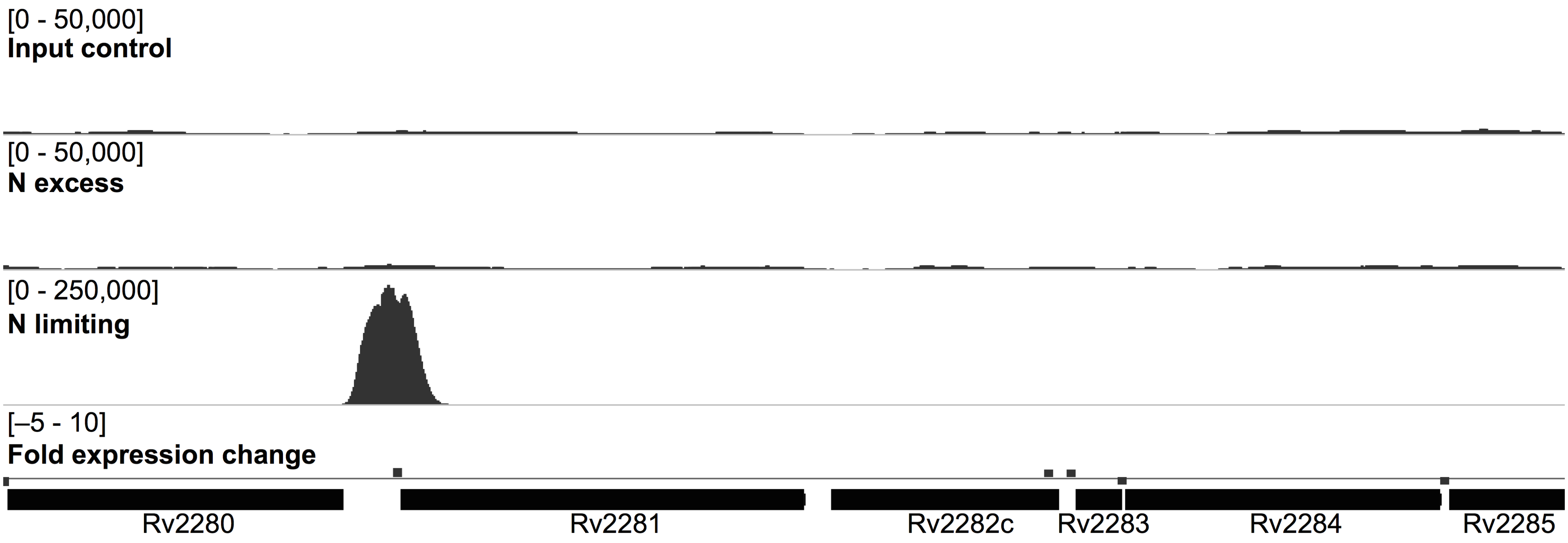
[0 - 50,000]
Input control
[0 - 50,000]
N excess
[0 - 250,000]
N limiting
[–5 - 10]
Fold expression change
Rv2280
Rv2281
Rv2282c
Rv2283
Rv2284
Rv2285

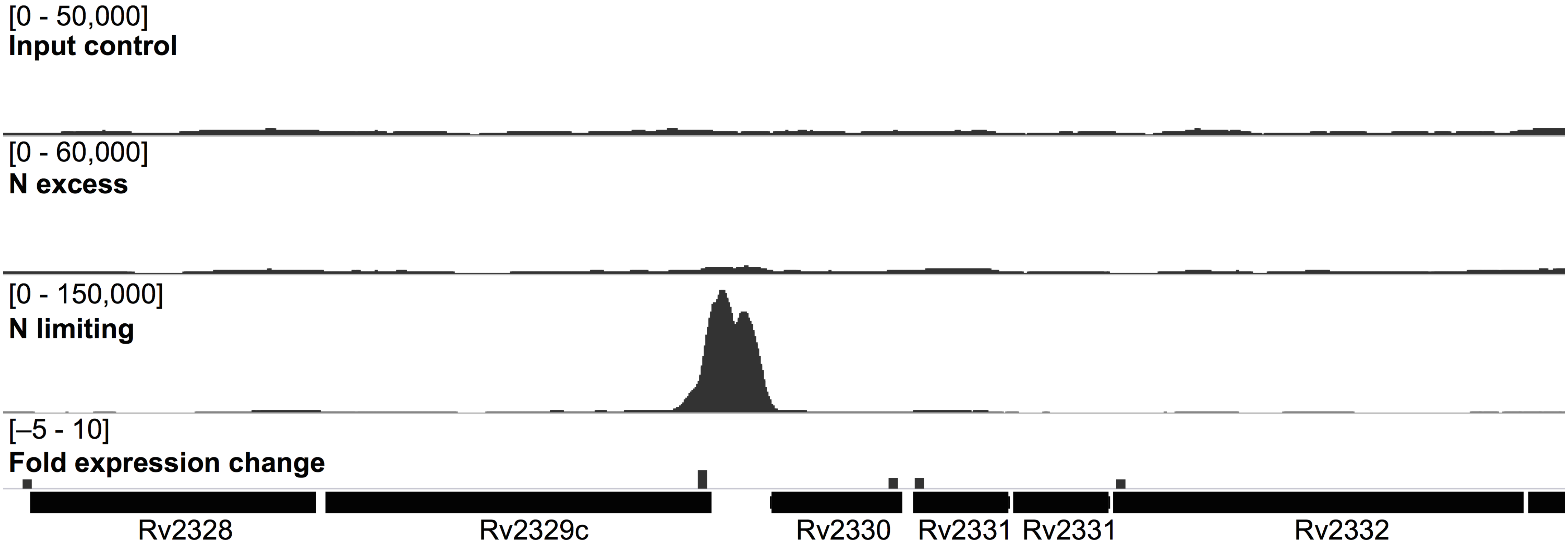
[0 - 50,000]
Input control
[0 - 60,000]
N excess
[0 - 150,000]
N limiting
[–5 - 10]
Fold expression change
Rv2328
Rv2329c
Rv2330
Rv2331
Rv2331
Rv2332

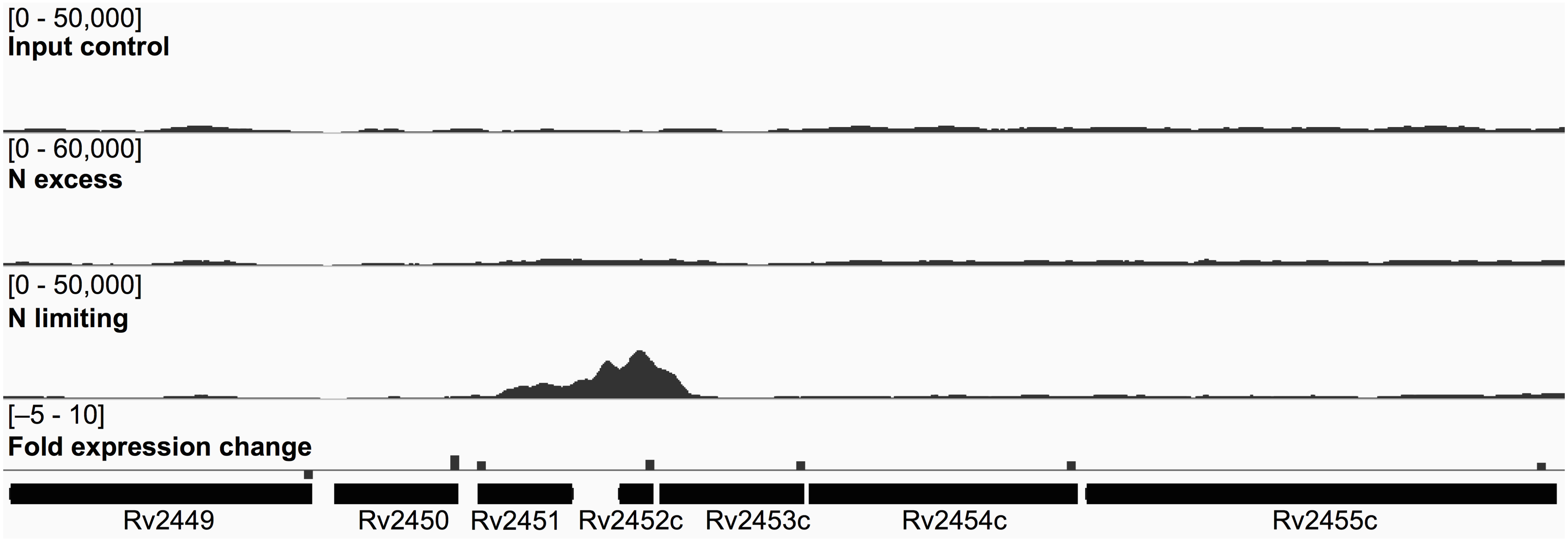
[0 - 50,000]
Input control
[0 - 60,000]
N excess
[0 - 50,000]
N limiting
[–5 - 10]
Fold expression change
Rv2449
Rv2450
Rv2451
Rv2452c
Rv2453c
Rv2454c
Rv2455c

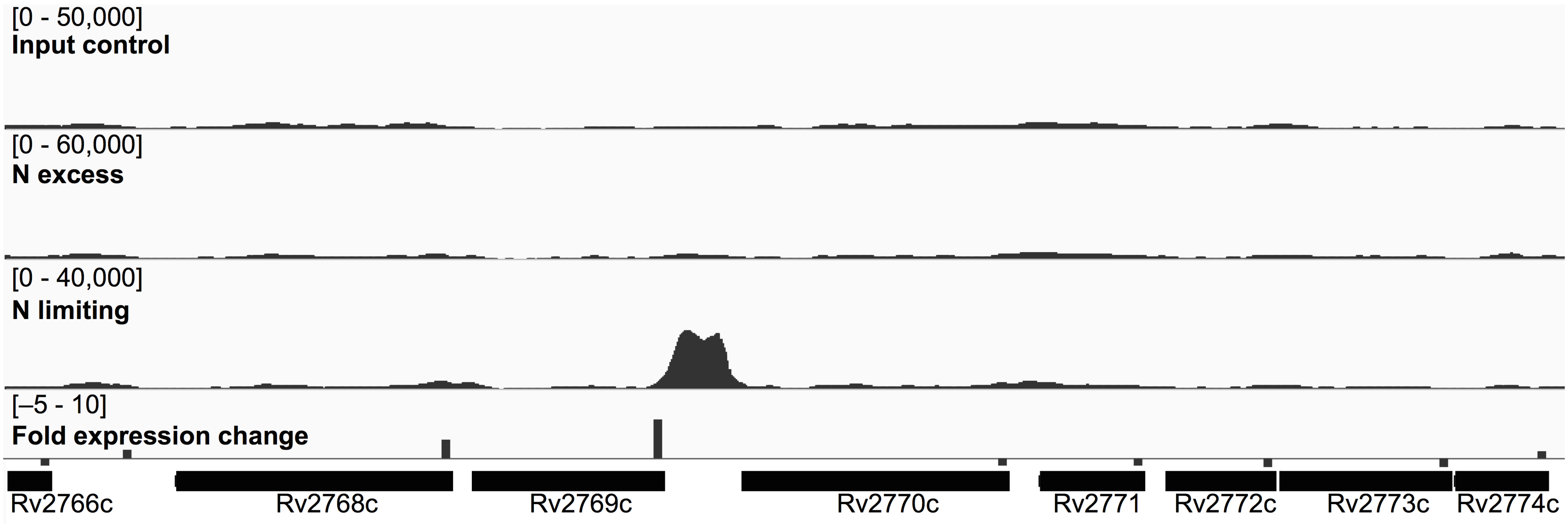

[0 - 50,000]
Input control
[0 - 60,000]
N excess
[0 - 40,000]
N limiting
[–5 - 10]
Fold expression change
Rv2766c
Rv2768c
Rv2769c
Rv2770c
Rv2771
Rv2772c
Rv2773c
Rv2774c

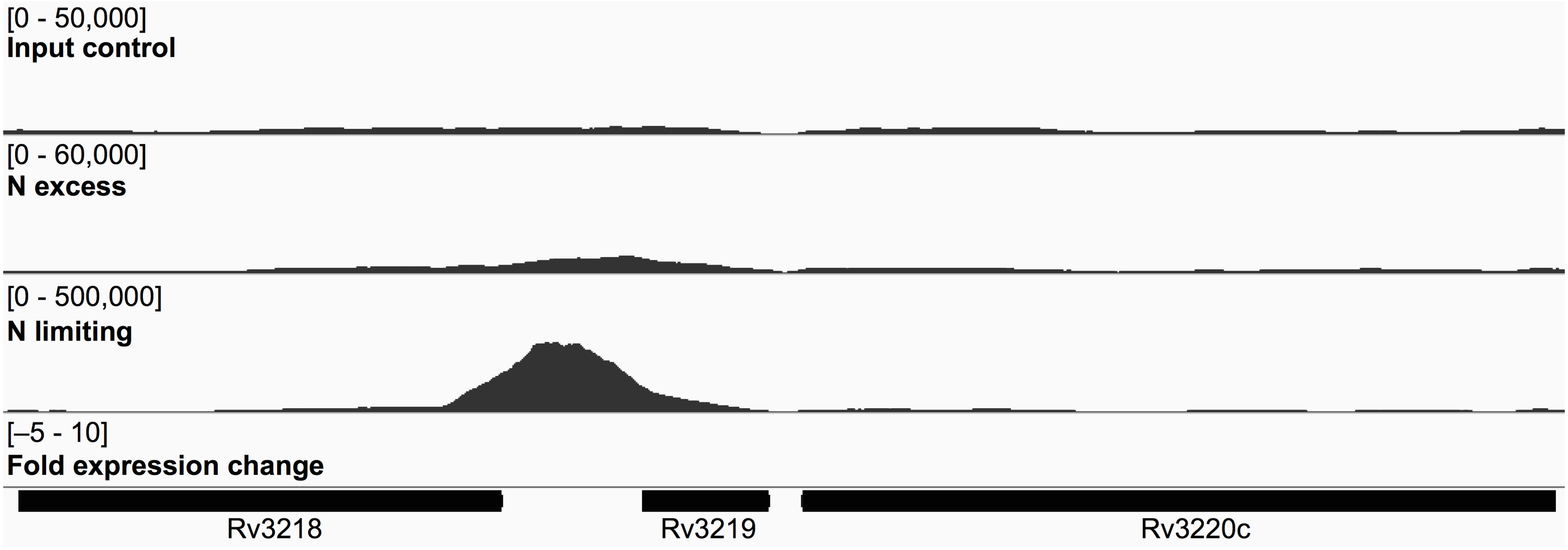
[0 - 50,000]
Input control
[0 - 60,000]
N excess
[0 - 500,000]
N limiting
[–5 - 10]
Fold expression change
Rv3218
Rv3219
Rv3220c

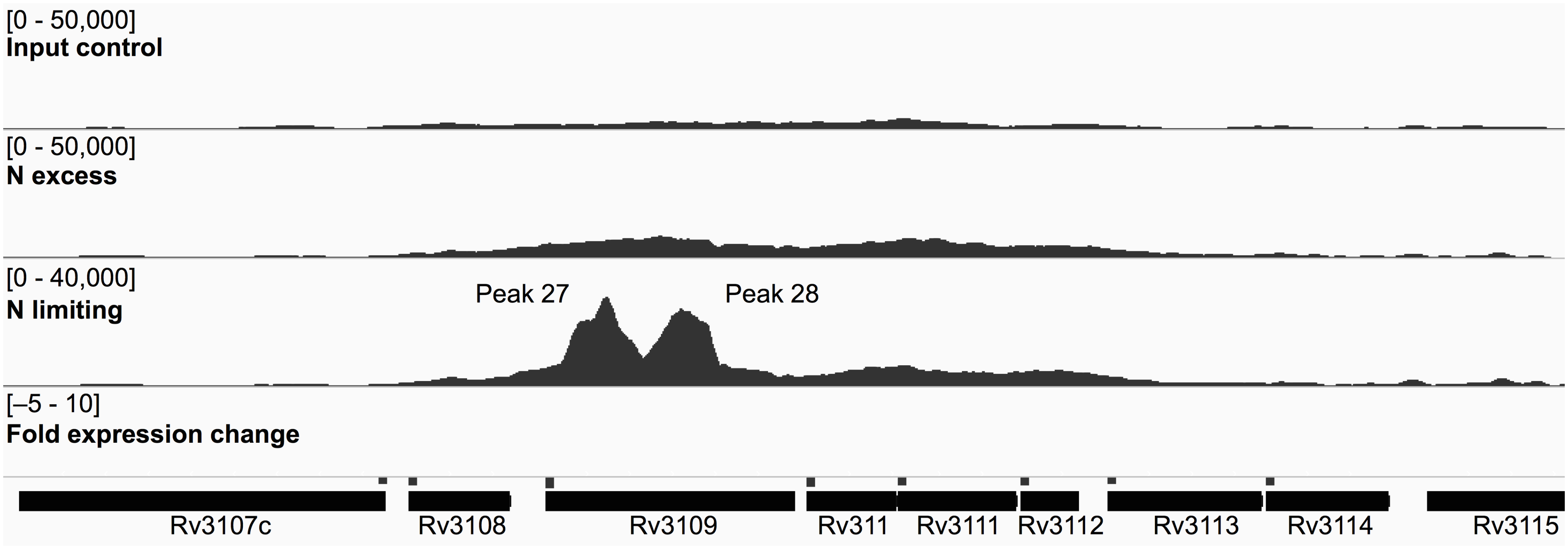
[0 - 50,000]
Input control
[0 - 50,000]
N excess
[0 - 40,000]
N limiting
Peak 27
Peak 28
[–5 - 10]
Fold expression change
Rv3107c
Rv3108
Rv3109
Rv311
Rv3111
Rv3112
Rv3113
Rv3114
Rv3115

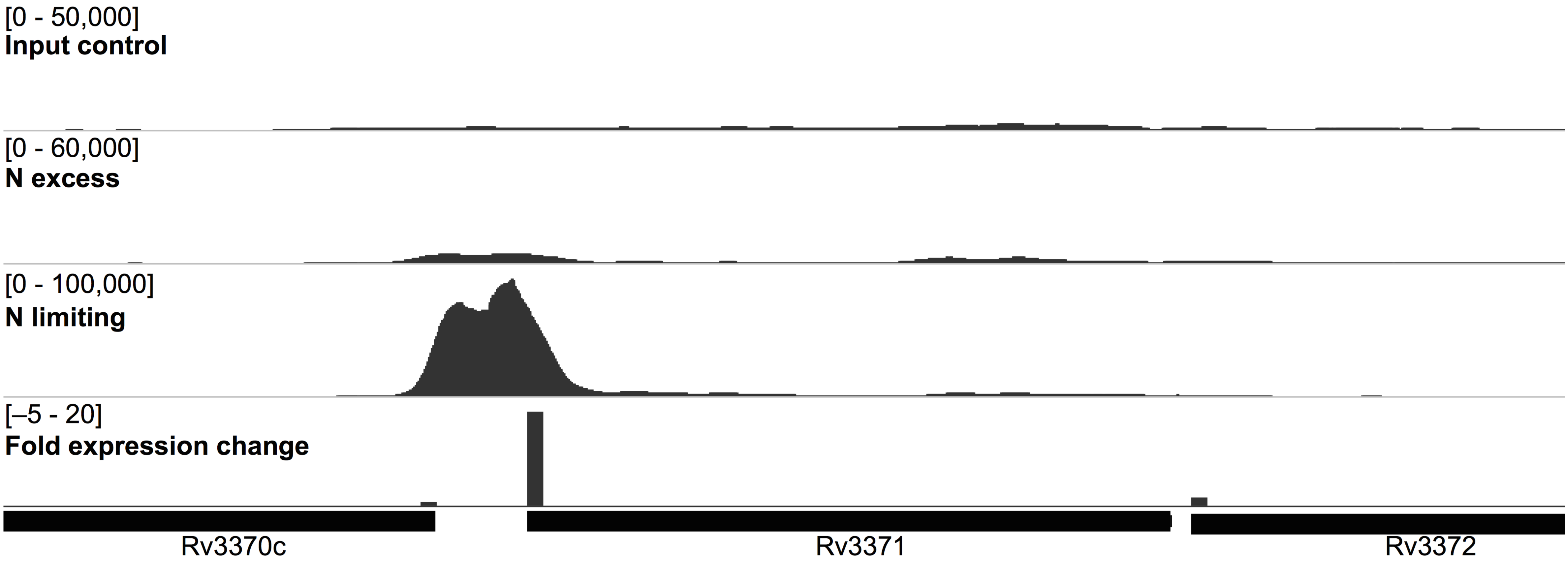
[0 - 50,000]
Input control
[0 - 60,000]
N excess
[0 - 100,000]
N limiting
[–5 - 20]
Fold expression change
Rv3370c
Rv3371
Rv3372

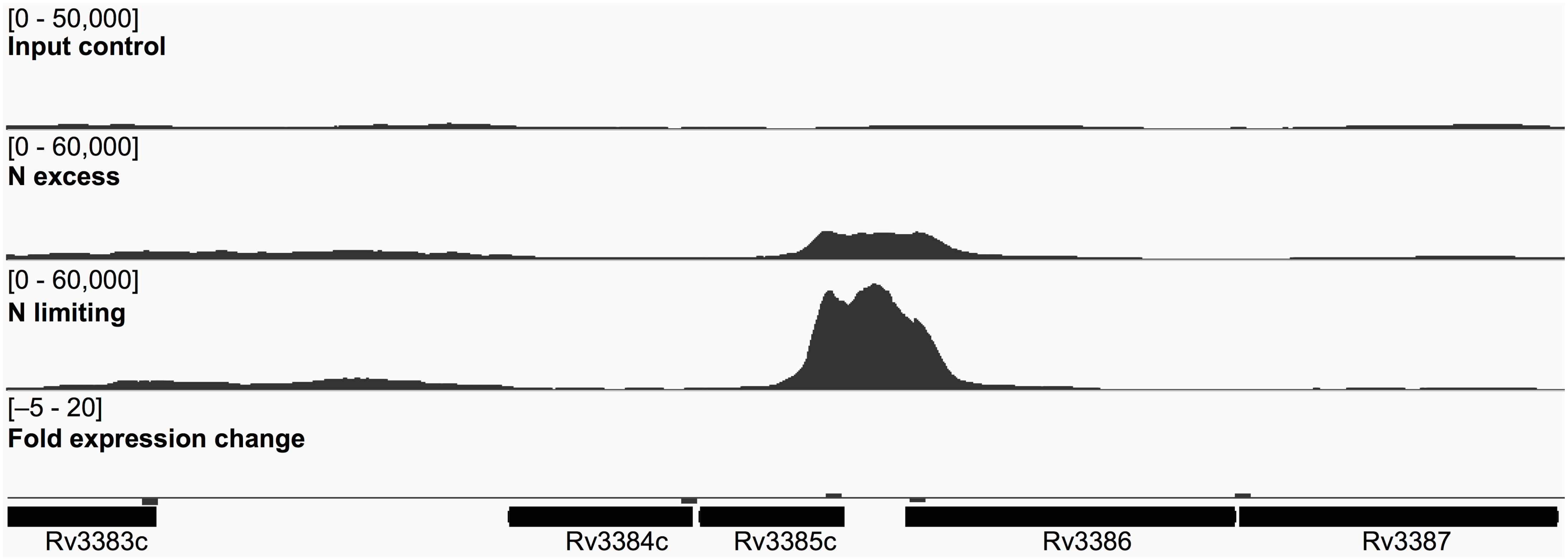
[0 - 50,000]
Input control
[0 - 60,000]
N excess
[0 - 60,000]
N limiting
[–5 - 20]
Fold expression change
Rv3383c
Rv3384c
Rv3385c
Rv3386
Rv3387

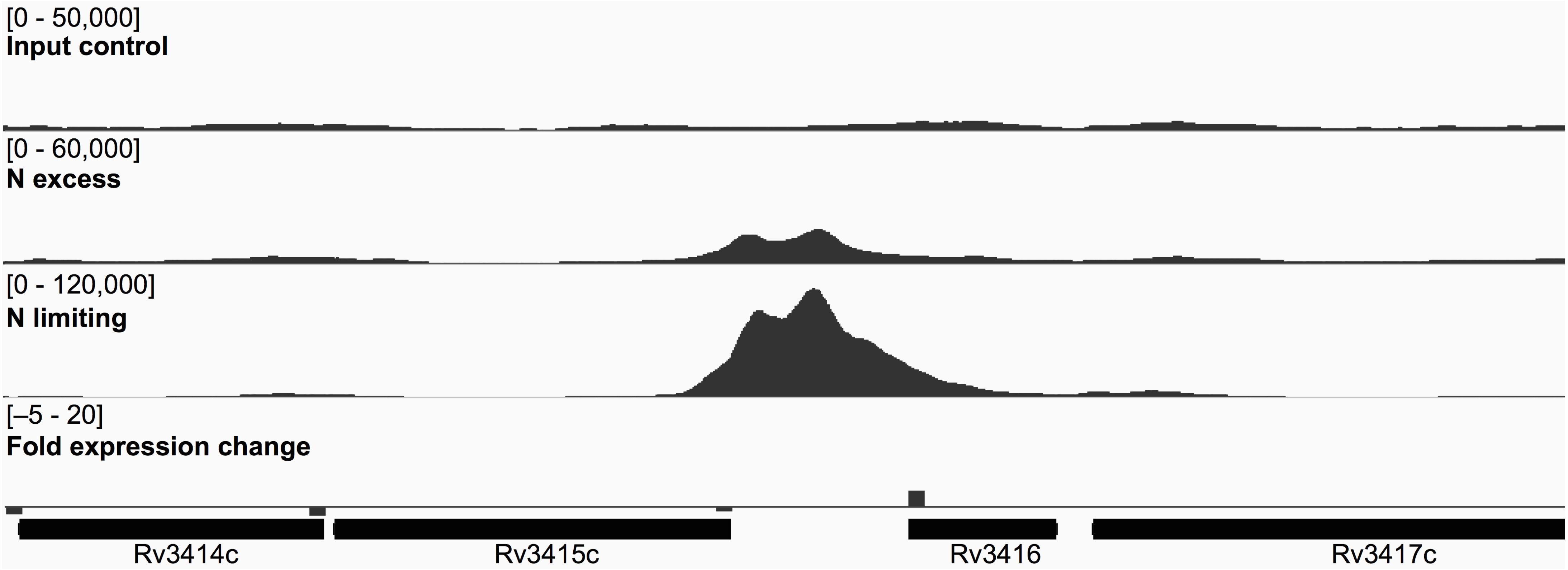
[0 - 50,000]
Input control
[0 - 60,000]
N excess
[0 - 120,000]
N limiting
[–5 - 20]
Fold expression change
Rv3414c
Rv3415c
Rv3416
Rv3417c

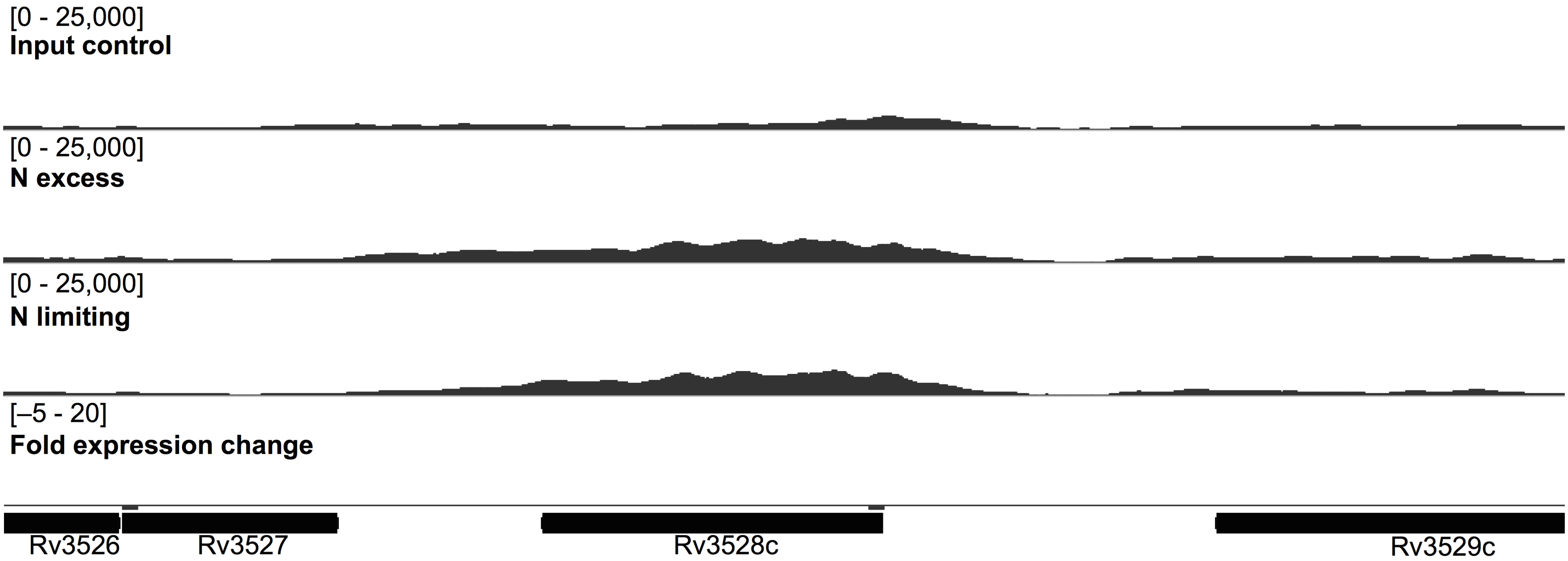
[0 - 25,000]
Input control
[0 - 25,000]
N excess
[0 - 25,000]
N limiting
[–5 - 20]
Fold expression change
Rv3526
Rv3527
Rv3528c
Rv3529c

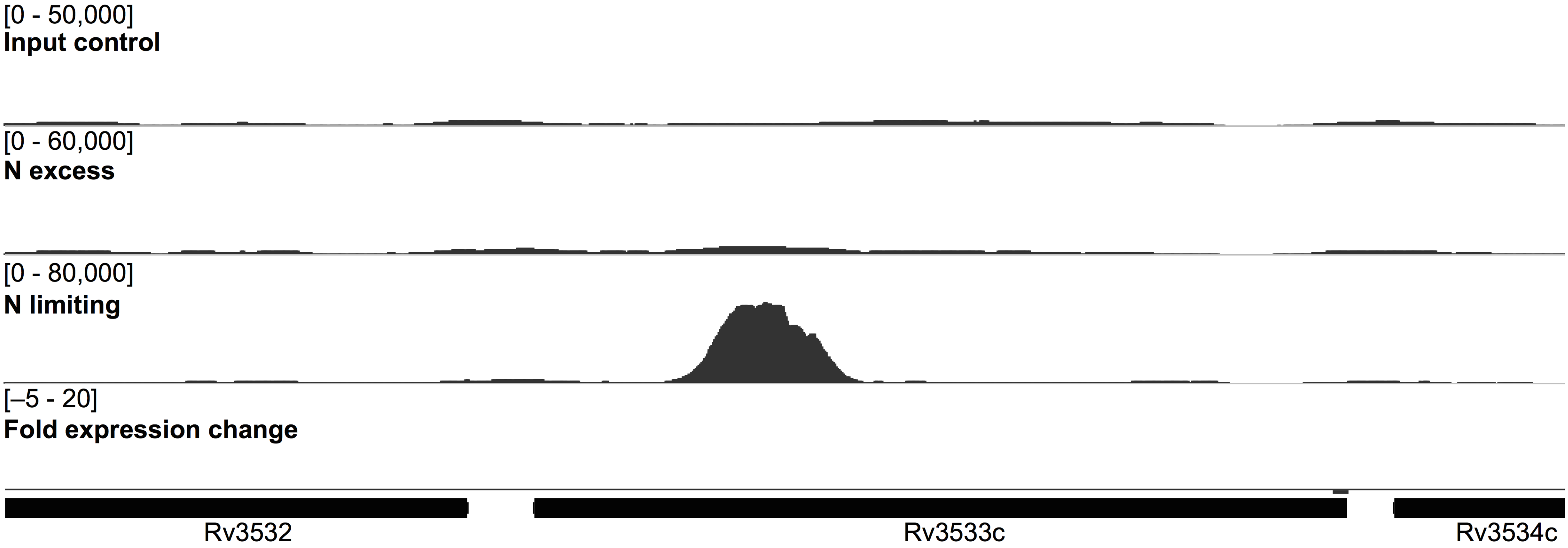

[0 - 50,000]
Input control
[0 - 60,000]
N excess
[0 - 80,000]
N limiting
[–5 - 20]
Fold expression change
Rv3532
Rv3533c
Rv3534c

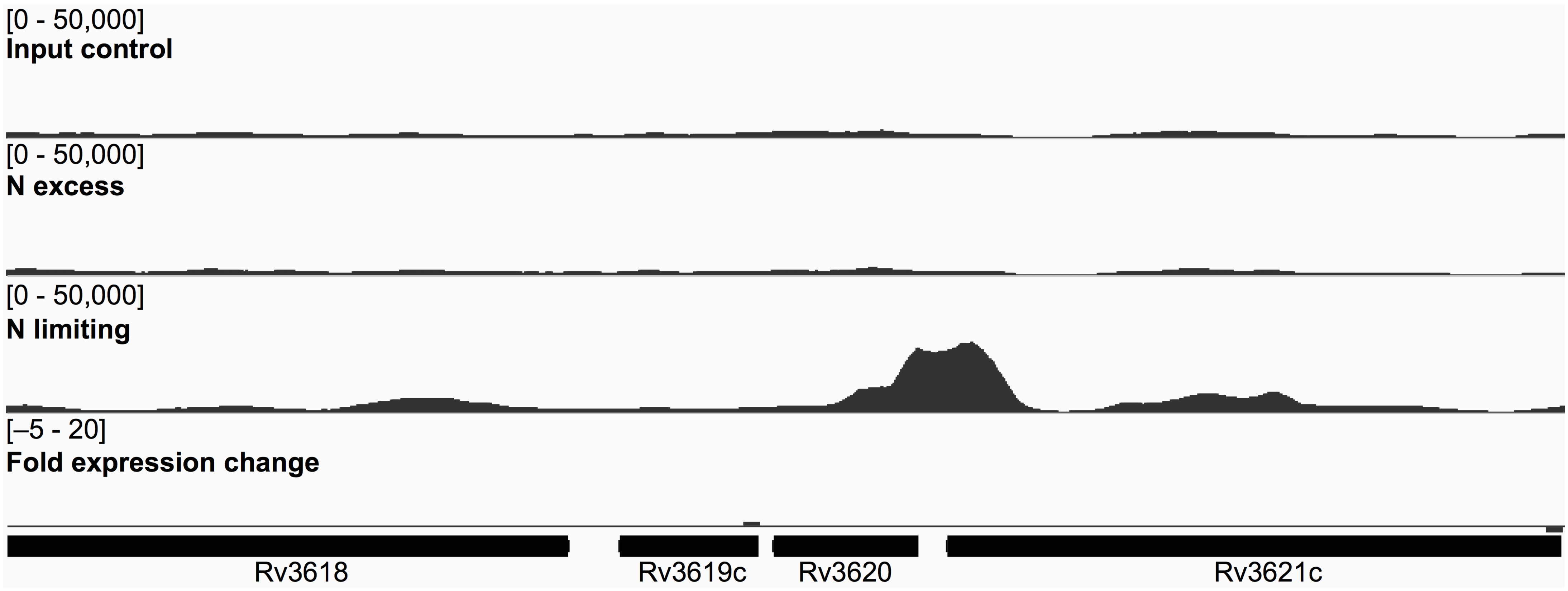

[0 - 50,000]
Input control
[0 - 50,000]
N excess
[0 - 50,000]
N limiting
[–5 - 20]
Fold expression change
Rv3618
Rv3619c
Rv3620
Rv3621c

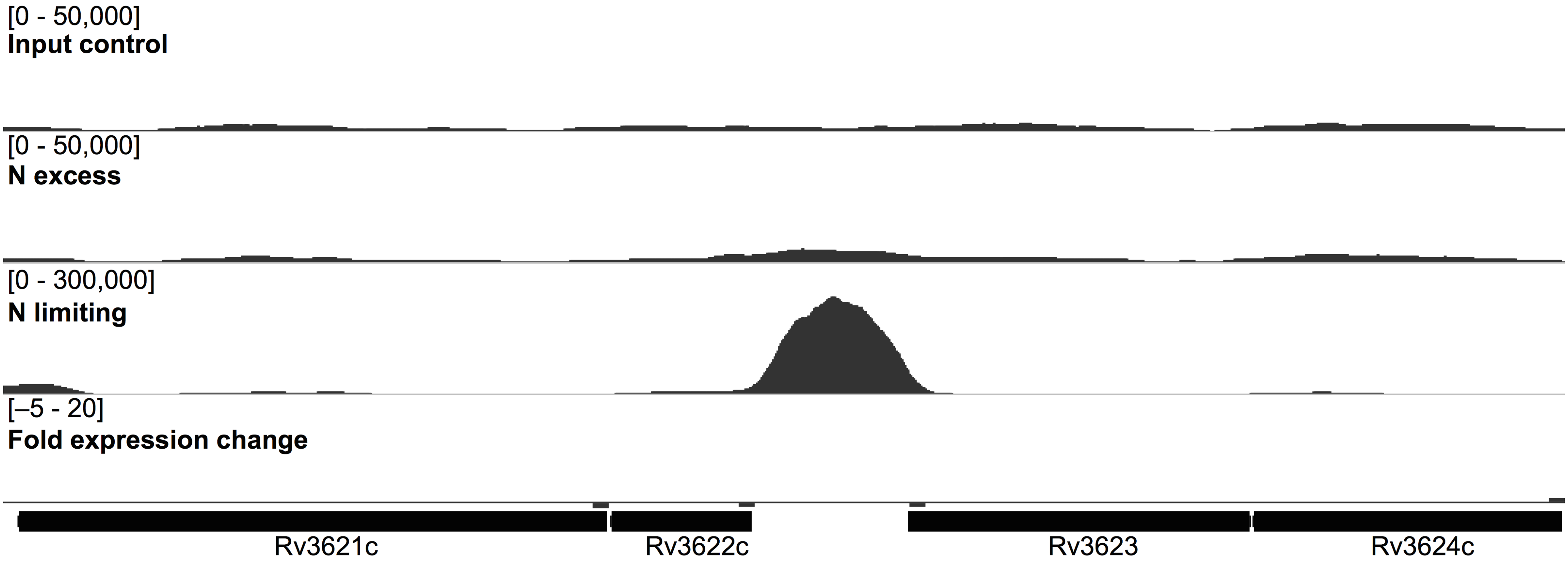
[0 - 50,000]
Input control
[0 - 50,000]
N excess
[0 - 300,000]
N limiting
[–5 - 20]
Fold expression change
Rv3621c
Rv3622c
Rv3623
Rv3624c
